# Calciprotein Particles Induce Cellular Compartment-Specific Proteome Alterations in Human Arterial Endothelial Cells

**DOI:** 10.3390/jcdd11010005

**Published:** 2023-12-22

**Authors:** Daria Shishkova, Arseniy Lobov, Egor Repkin, Victoria Markova, Yulia Markova, Anna Sinitskaya, Maxim Sinitsky, Egor Kondratiev, Evgenia Torgunakova, Anton Kutikhin

**Affiliations:** 1 Department of Experimental Medicine, Research Institute for Complex Issues of Cardiovascular Diseases, 6 Sosnovy Boulevard, 650002 Kemerovo, Russia; shidk@kemcardio.ru (D.S.); markve@kemcardio.ru (V.M.); markya@kemcardio.ru (Y.M.); cepoav@kemcardio.ru (A.S.); sinimy@kemcardio.ru (M.S.); kondea@kemcardio.ru (E.K.); torgea@kemcardio.ru (E.T.); 2 Laboratory of Regenerative Biomedicine, Institute of Cytology of the RAS, 4 Tikhoretskiy Prospekt, 194064 St. Petersburg, Russia; lobov@incras.ru; 3 Centre for Molecular and Cell Technologies, St. Petersburg State University, Universitetskaya Embankment, 7/9, 199034 St. Petersburg, Russia; st049553@student.spbu.ru

**Keywords:** calciprotein particles, mineral stress, endothelial cells, proteomic profiling, cytosol, nuclei, mitochondria, lysosomes, endoplasmic reticulum, molecular signatures

## Abstract

Calciprotein particles (CPPs) are indispensable scavengers of excessive Ca^2+^ and PO_4_^3−^ ions in blood, being internalised and recycled by liver and spleen macrophages, monocytes, and endothelial cells (ECs). Here, we performed a pathway enrichment analysis of cellular compartment-specific proteomes in primary human coronary artery ECs (HCAEC) and human internal thoracic artery ECs (HITAEC) treated with primary (amorphous) or secondary (crystalline) CPPs (CPP-P and CPPs, respectively). Exposure to CPP-P and CPP-S induced notable upregulation of: (1) cytokine- and chemokine-mediated signaling, Ca^2+^-dependent events, and apoptosis in cytosolic and nuclear proteomes; (2) H^+^ and Ca^2+^ transmembrane transport, generation of reactive oxygen species, mitochondrial outer membrane permeabilisation, and intrinsic apoptosis in the mitochondrial proteome; (3) oxidative, calcium, and endoplasmic reticulum (ER) stress, unfolded protein binding, and apoptosis in the ER proteome. In contrast, transcription, post-transcriptional regulation, translation, cell cycle, and cell–cell adhesion pathways were underrepresented in cytosol and nuclear compartments, whilst biosynthesis of amino acids, mitochondrial translation, fatty acid oxidation, pyruvate dehydrogenase activity, and energy generation were downregulated in the mitochondrial proteome of CPP-treated ECs. Differentially expressed organelle-specific pathways were coherent in HCAEC and HITAEC and between ECs treated with CPP-P or CPP-S. Proteomic analysis of mitochondrial and nuclear lysates from CPP-treated ECs confirmed bioinformatic filtration findings.

## 1. Introduction

Calciprotein particles (CPPs) represent a mineral buffer system that controls the concentration of Ca^2+^ ions in the human blood through the reaction between Ca^2+^, PO_4_^3−^, and acidic serum proteins termed as mineral chaperones, among which the most potent are fetuin-A and albumin [1,2,3,4,5,6,7,8,9,10]. As such, CPPs are amorphous mineralo-organic particles scavenging the excessive Ca^2+^ and PO_4_^3−^ ions from the bloodstream and being recycled by resident macrophages of the liver and spleen [11,12,13], monocytes [14], and endothelial cells (ECs) [14,15,16,17,18,19]. Collectively, CPPs (i.e., protein-covered calcium phosphate aggregates), calciprotein monomers (i.e., small calcium phosphate clusters bound to fetuin-A or albumin), and mineral chaperones (i.e., acidic proteins binding free Ca^2+^ ions) comprise an efficient mineral buffering system which participates in the regulation of mineral homeostasis and prevents extraskeletal calcification [10,20]. However, depletion in mineral chaperones (e.g., observable at hypoproteinemia in patients with pre-dialysis or end-stage kidney disease) or overwhelming of their buffering capability by an uncurbed release of Ca^2+^ ions into the blood (e.g., occurring in patients with osteopenia/osteoporosis) leads to the blood supersaturation with Ca^2+^ and PO_4_^3−^ ions. This, in turn, results in the conversion of amorphous and spherical primary CPPs (CPP-P) into hazardous crystalline and spindle-shaped secondary CPPs (CPP-S), ultimately provoking endothelial dysfunction [14,15,16,17,18,19] and ectopic calcification [21,22]. Biochemical indicators of calcium overload such as ionised calcium (Ca^2+^) level measured by colorimetry or potentiometry, T_50_ value defining the rate of amorphous-to-crystalline transition of CPPs (transformation of CPP-P into CPP-S) [23,24,25,26,27,28,29], optical density (OD_650_) increment after ex vivo supersaturation of serum with Ca^2+^ and PO_4_^3−^ ions [18], and OsteoSense fluorescent-labeled bisphosphonate probe binding to CPPs and calciprotein monomers [30,31,32,33,34,35,36] have been consistently associated with major adverse cardiovascular events [18,24,26,37,38]. Taken together, these observations show the pathophysiological relevance of CPP generation in the human blood, underscoring the need to counteract its deregulation.

Previously, our group demonstrated a pro-inflammatory response in monocytes [14] and ECs [14,17,18] treated with CPPs in laminar flow conditions, as well as in rats which received intravenous injections of CPPs [14]. This response pattern corresponded to the scenario of chronic low-grade age-associated inflammation frequently mentioned as inflammaging [39,40,41]. The molecular basis of endothelial activation upon CPP internalisation includes partial or complete dissolution of CPPs in lysosomes, a massive influx of Ca^2+^ ions into the cytosol, inflammasome activation, mitochondrial outer membrane permeabilisation, oxidative stress, and caspase-mediated cell death if calcium stress is uncurbed [7,8,9,10,11]. Proteomic profiling of primary human coronary artery ECs (HCAEC) and human internal thoracic artery ECs (HITAEC) revealed significant differences in molecular portfolio between CPP-P- or CPP-S-treated and control ECs [7]. Yet, specific cytosolic, nuclear, mitochondrial, lysosomal, and endoplasmic reticulum (ER) molecular signatures of ECs which internalised excessive amounts of CPPs have not been investigated hitherto.

Here we performed a pathway enrichment analysis of label-free proteomic profiling data of CPP-P- and CPP-S-treated ECs [14] in relation to putative mitochondrial, lysosomal, and ER dysfunction and accompanying molecular alterations in nuclear and cytosolic protein composition. We found that upregulated pathways in mitochondria, lysosomes, and ER proteomes mediated the stress response after the internalisation of CPP-P or CPP-S by the ECs (i.e., H^+^ and Ca^2+^ translocation, generation of reactive oxygen species, unfolded protein response, mitochondrial outer membrane permeabilisation, and intrinsic apoptosis). Cytosolic and nuclear response was primarily focused on downregulation of cellular homeostasis pathways (i.e., transcription, RNA metabolism, translation, and cell cycle). However, the molecular signatures of cytokine- and chemokine-mediated signaling, Ca^2+^-dependent events, and regulated cell death were notable in cytosolic and nuclear proteomes as well. Contrariwise, lysosomal proteome response to CPP internalisation in the ECs was relatively mild, suggesting that it exploits existing protein machinery rather than relying on transcriptional, post-transcriptional, or translational mechanisms. Upregulated or downregulated cellular compartment-specific pathways corresponded between distinct EC lines (i.e., HCAEC and HITAEC) and CPP types (i.e., CPP-P or CPP-S). To confirm the key findings, we conducted proteomic analysis of pre-fractionated mitochondrial and nuclear lysates from CPP-treated HCAEC. The experimental data supported bioinformatic filtration findings, as cellular senescence induced by oxidative and telomere stress, senescence-associated secretory phenotype, Ca^2+^ binding, and programmed cell death were upregulated in mitochondrial lysate along with the downregulation of protein folding, cell redox homeostasis, and energy generation. Similarly, analysis of ECs nuclear lysates revealed upregulation of calcium stress response, oxidative- and telomere stress-induced senescence, unfolded protein response and apoptosis in conjunction with downregulation of transcription, post-transcriptional regulation, DNA repair, and cell cycle pathways. Therefore, our study uncovered the molecular basis of CPP-triggered EC response through the unbiased, high-throughput, and holistic proteomic approach.

## 2. Materials and Methods

### 2.1. Artificial Synthesis and Quantification of CPPs

Artificial synthesis of CPP-P and CPP-S was carried out as in [14]. To synthesise CPP-P and CPP-S, stock solutions of CaCl_2_ (21115, Sigma-Aldrich, Saint Louis, MO, USA) and Na_2_HPO_4_ (94046, Sigma-Aldrich, Saint Louis, MO, USA) were diluted to equal concentrations of 3 (CPP-P) or 7.5 (CPP-S) mmol/L in Dulbecco’s modified Eagle’s medium (DMEM, 31330038, Thermo Fisher Scientific, Waltham, MA, USA) supplemented with 10% (CPP-P) or 1% fetal bovine serum (CPP-S). The reagents were added into DMEM in the following order: (1) FBS; (2) CaCl_2_; (3) Na_2_HPO_4_, with a vortexing between the added reagents. Following incubation for 24 h in cell culture conditions, the medium was centrifuged at 200,000× *g* for 1 h (Optima MAX-XP, Beckman Coulter, Brea, CA, USA), and the particle sediment was resuspended in the sterile phosphate-buffered saline (PBS, pH = 7.4, 2.1.1, BioLot, St. Petersburg, Russia).

Quantification of CPP-P and CPP-S was performed as in [18]. Briefly, the concentration of CPP-P and CPP-S was ≈1.2 × 10^3^ particles per µL suspension. Each CPP aliquote was examined by scanning electron microscopy (Hitachi S-3400N, Hitachi, Tokyo, Japan) and transmission electron microscopy (JEM-4000 EX, JEOL, Tokyo, Japan) through diluting 5 µL of the abovementioned CPP solution with 495 µL sterile-filtered double distilled water (1:100 dilution) the day before the respective experiments to verify CPP appearance and control potential maturation of CPP-P to CPP-S in the solution. The appearance of CPP-P and CPP-S was similar to that previously shown [18] (Supplementary Figure S1).

### 2.2. Cell Culture

Primary HCAEC (300K-05a, Cell Applications, San Diego, CA, USA) and human coronary artery vascular smooth muscle cells (VSMCs; here we used an HCASMC line, 350K-05a, Cell Applications, San Diego, CA, USA) were grown in T-75 flasks (90076, Techno Plastic Products, Trasadingen, Switzerland) according to the manufacturer’s protocol, using MesoEndo Growth Medium (for HCAEC: 212-500, Cell Applications, San Diego, CA, USA) or human SMC Growth Medium (for HCASMC: 311-500, Cell Applications, San Diego, CA, USA) and subculture reagent kit (090K, Cell Applications, San Diego, CA, USA). Immediately before the experiments, we replaced MesoEndo Growth Medium and human SMC Growth Medium with MesoEndo Growth Medium without FBS (212F-500, Cell Applications, San Diego, CA, USA) and human SMC Basal Medium (310-500, Cell Applications, San Diego, CA, USA), respectively. During such replacement, we washed cells twice with warm (≈37 °C) PBS to remove the residual serum components which could affect further proteomic profiling.

### 2.3. Treatment of ECs and VSMCs with Calciprotein Particles

HCAEC and HCASMC were cultured in 6-well plates (92406, Techno Plastic Products, Trasadingen, Switzerland) to ≈90% confluence (≈0.5 × 10^6^ cells per well) and were then exposed to 100 µL CPP-P, CPP-S (0.6 × 10^5^ particles per mL or 25 µg/mL calcium), or PBS (*n* = 3 wells per group) in a serum-free medium (212F-500, Cell Applications, San Diego, CA, USA) for 24 h. As we have shown earlier [14], such a dose of CPP-P and CPP-S corresponded to a 15–25% increase above physiological CPP serum level (2.5 × 10^5^ particles per mL). Such an increase has been previously documented in patients with end-stage renal disease [30]. The rationale behind using a 24 h time point was the need to detect all possible changes in the biochemical pathways, as we investigated protein (but not mRNA) response, and alterations in the proteomic signatures generally follow shifts occurring in transcriptional programs and post-transcriptional regulation. In addition, different organelles might have distinct time-resolved patterns of molecular response to CPP treatment. Employing the mentioned time point (i.e., 24 h), we were able to encompass delayed endothelial response to CPP-P and CPP-S during the proteomic profiling. For instance, inflammasome assembly occurs only ≈8 h after CPP-S exposure [12,13]. Cell cultures were washed with ice-cold (4 °C) PBS (pH = 7.4, 2.1.1, BioLot, St. Petersburg, Russia) and lysed in either RIPA buffer (89901, Thermo Fisher Scientific, Waltham, MA, USA) supplemented with protease and phosphatase inhibitors (78444, Thermo Fisher Scientific, Waltham, MA, USA) to extract total protein (HCAEC), or in ExtractRNA reagent (BC032, Evrogen, Moscow, Russia) to extract RNA (HCASMC), according to the manufacturer’s protocols.

### 2.4. Bioinformatic Analysis of Cellular Compartment-Specific Proteomes in HCAEC and HITAEC Treated with CPPs

To explore differential expression patterns within specific cellular compartments, providing a more refined view of the proteomic landscape, we re-analysed shotgun proteomics data (the dataset identifier PXD038017), described by us to a lesser extent before [14]. Since the coronary artery is atheroprone and the internal thoracic artery is atheroresistant [42,43], as previously, here we also compared two EC lines (HCAEC and HITAEC) [14].

To perform bioinformatic analysis, we used label-free quantification by peak area under the curve, deposited to the ProteomeXchange Consortium via the PRIDE [44] repository [14], for further analysis in R (version 3.6.1; R Core Team, 2019) [45]. The proteins with missed values in ≥20% of samples were removed and the imputation of missed values by the k-nearest neighbours was performed by the “impute” package [46]. To focus specifically on various compartments, we separated the dataset into five groups based on the protein subcellular location data from UniProt: cytoplasm, lysosome, mitochondria, endoplasmic reticulum (ER), and nucleus. As soon as many proteins have more than one main subcellular localisation, proteins might be presented in more than one group. Then, all these five datasets were analysed in the same way separately.

We performed the log-transformation and quantile normalisation with further analysis of differential expression by the “limma” package [47]. Then, we carried out clusterisation of samples by principal component analysis (PCA) and sparse partial least squares discriminant analysis (sPLS-DA) in the “MixOmics” package [48]. “ggplot2” [49] and “EnhancedVolcano” [50] packages were used for visualisation. Differentially expressed proteins (DEPs) were defined as those with logarithmic fold change ≥ 1 and BH-corrected *p* value ≤ 0.05.

Bioinformatic analysis was performed using the Gene Ontology [51,52], Reactome [53,54], UniProtKB Keywords [55], and Kyoto encyclopaedia of genes and genomes (KEGG) databases [56,57] screened employing the Database for Annotation, Visualization and Integrated Discovery (DAVID) [58,59]. Bioinformatic filtration was conducted in October 2023, considering the constant updates of the mentioned bioinformatic databases, including organellar and sub-organellar protein localisation annotations [60,61]. For the filtration of bioinformatic pathways, we applied an Expression Analysis Systematic Explorer (EASE) score, a conservative adjustment to the Fisher exact probability, which is calculated by removing one gene within the given category from the list and calculating the resulting Fisher exact probability for that category [58,59,62]. EASE score is a measure automatically calculated by the DAVID database for pathway enrichment purposes [58,59]. In this study, we used an EASE score of 0.05 as a statistical significance threshold for maximum enrichment with pathways having a low number of proteins, although the false discovery rate has also been calculated for convenience.

### 2.5. Shotgun Proteomics Analysis of HCAEC Nuclear and Mitochondrial Fraction

HCAECs were cultured in T-150 flasks (90552, Techno Plastic Products, Trasadingen, Switzerland) to ≈90% confluence (≈7.5 × 10^6^ cells per flask) and were then exposed to 1500 µL CPP-P, CPP-S (0.6 × 10^5^ particles per mL or 25 µg/mL calcium), or PBS in a serum-free medium (212F-500, Cell Applications, San Diego, CA, USA) for 24 h. Cell cultures were washed with ice-cold (4 °C) PBS (pH = 7.4, 2.1.1, BioLot, St. Petersburg, Russia), and cells were trypsinised (090K, Cell Applications, San Diego, CA, USA), collected, fractionated into mitochondrial and nuclear compartments, and then lysed using the respective cell fractionation kit (ab109719, Abcam, Cambridge, UK) according to the manufacturer’s protocol. Quantification of total protein was conducted using the BCA Protein Assay Kit (23227, Thermo Fisher Scientific, Waltham, MA, USA) and Multiskan Sky microplate spectrophotometer (Thermo Fisher Scientific, Waltham, MA, USA) in accordance with the manufacturer’s protocol.

To prepare the samples for the tryptic digestion, we removed the RIPA buffer or fractionation lysis buffers by acetone precipitation (650501, Sigma-Aldrich, Saint Louis, MO, USA) and resuspended protein pellets in 8 mol/L urea (U5128, Sigma-Aldrich, Saint Louis, MO, USA) diluted in 50 mmol/L ammonium bicarbonate (09830, Sigma-Aldrich, Saint Louis, MO, USA). The protein concentration was measured by a Qubit 4 fluorometer (Q33238, Thermo Fisher Scientific, Waltham, MA, USA) with a QuDye Protein Quantification Kit (25102, Lumiprobe, Cockeysville, MD, USA), according to the manufacturer’s protocol. Protein samples (15 µg) were then incubated in 5 mmol/L dithiothreitol (D0632, Sigma-Aldrich, Saint Louis, MO, USA) for 1 h at 37 °C with the subsequent incubation in 15 mmol/L iodoacetamide for 30 min in the dark at room temperature (I1149, Sigma-Aldrich, Saint Louis, MO, USA). Next, the samples were diluted with 7 volumes of 50 mmol/L ammonium bicarbonate and incubated for 16 h at 37 °C with 200 ng of trypsin (1:50 trypsin:protein ratio; VA9000, Promega, Madison, WI, USA). The peptides were then frozen at −80 °C for 1 h and desalted with stage tips (Tips-RPS-M.T2.200.96, Affinisep, Le Houlme, France), according to the manufacturer’s protocol using methanol (1880092500, Sigma-Aldrich, Saint Louis, MO, USA), acetonitrile (1000291000, Sigma-Aldrich, Saint Louis, MO, USA), and 0.1% formic acid (33015, Sigma-Aldrich, Saint Louis, MO, USA). Desalted peptides were dried in a centrifuge concentrator (Concentrator plus, Eppendorf, Hamburg, Germany) for 3 h and finally dissolved in 20 µL 0.1% formic acid for further shotgun proteomics analysis.

Shotgun proteomics analysis was performed by ultra-high performance liquid chromatography-tandem mass spectrometry (UHPLC-MS/MS) with ion mobility in a TimsToF Pro mass spectrometer with the nanoElute UHPLC system (Bruker Daltonics, Billerica, MA, USA) using ≈500 ng of peptides. UHPLC was performed in the one-column separation mode with an Aurora Series separation column with nanoZero technology (C18, 25 cm × 75 μm ID, 1.6 μm C18; IonOpticks, Melbourne, Australia) in a gradient mode with 400 nL/min flow rate and 55 °C. Phase A was water/0.1% formic acid, and phase B was acetonitrile/0.1% formic acid (1000291000, Sigma-Aldrich, Saint Louis, MO, USA). The gradient was from 2% to 37% phase B for 50 min with subsequent washing with 85% phase B for 10 min. Before each sample, the separation columns were equilibrated with 4 column volumes. CaptiveSpray ion source was used for electrospray ionisation with 1600 V of capillary voltage, 3 L/min N_2_ flow, and 180 °C source temperature. The mass spectrometry acquisition was performed in DDA-PASEF mode with a 1.1 s cycle in positive polarity with the fragmentation of ions with at least two charges in an *m*/*z* range from 100 to 1700 and ion mobility range from 0.60 to 1.60 1/K0.

Protein identification was performed in FragPipe software (version 18.0) using MSFragger (version 3.5) and Philosopher (version 4.4.0) in Windows 10 OS with Java v. 11.0.9.1. The search was performed according to default LFQ-MBR DDA-PASEF workflow using human reference proteome UP000005640 (uploaded 05.04.2022).

The search parameters were as follows: parent and fragment mass error tolerance 20 and 10 ppm, respectively, protein and peptide false discovery rate less than 1%, protease rule-trypsin (cleave after KR), and 2 possible missed cleavage sites. Cysteine carbamidomethylation was set as a fixed modification. Methionine oxidation and acetylation of protein N-term were set as variable modifications.

The mass spectrometry proteomics data have been deposited to the ProteomeXchange Consortium via the PRIDE [44] partner repository with the dataset identifier PXD047581.

To support the key findings, we performed proteomic analysis of pre-fractionated mitochondrial and nuclear lysates from CPP-treated HCAEC. Bioinformatics analysis was performed separately for mitochondrial and nuclear fractions, similar as described above (Section 2.4), but without separation of the dataset to subcellular compartments.

### 2.6. Gene Expression Analysis

Gene expression analysis in CPP-P- and CPP-S-treated HCASMC has been performed by reverse transcription-polymerase chain reaction (RT-qPCR) as in [63]. Briefly, a High-Capacity cDNA Reverse Transcription Kit (4368814, Thermo Fisher Scientific, Waltham, MA, USA) was used for the reverse transcription, and RT-qPCR was carried out employing customised primers (500 nmol/L each, Evrogen, Moscow, Russia, Supplementary Table S1), cDNA (20 ng), and PowerUp SYBR Green Master Mix (A25778, Thermo Fisher Scientific, Waltham, MA, USA) according to the manufacturer’s protocol for T_m_ ≥ 60 °C (fast cycling mode). Technical replicates (*n* = 3 per each sample) were performed in all qPCR experiments. Quantification of the mRNA levels was performed by calculation of ΔCt and by using the 2^−ΔΔCt^ method. Relative transcript levels were expressed as a value relative to the average of three housekeeping genes (*GAPDH*, *ACTB*, and *B2M*) and to the PBS-treated group (2^−ΔΔCt^).

### 2.7. Western Blotting

Verification of successful fractionation in HCAEC was carried out by chemiluminescent Western blotting as in [17]. MagicMark XP Western protein standard (LC5602, Thermo Fisher Scientific, Waltham, MA, USA) was loaded as a molecular weight marker. Protein separation and transfer were conducted by sodium dodecyl sulfate–polyacrylamide gel electrophoresis (SDS-PAGE) and a dry blotting system (iBlot 2 Gel Transfer Device, Thermo Fisher Scientific, Waltham, MA, USA), using polyvinylidene difluoride transfer stacks (IB24001, Thermo Fisher Scientific, Waltham, MA, USA) as previously described [17]. Blocking of non-specific binding was carried out by incubation of polyvinylidene difluoride membranes in iBind Flex Solution (SLF2020, Solution Kit Thermo Fisher Scientific, Waltham, MA, USA) for 1 h. The blots were probed with (1) mouse monoclonal antibodies to proliferating cell nuclear antigen (PCNA, 1:1000 dilution, ab280088, Abcam, Cambridge, UK); (2) mouse monoclonal antibodies to TATA-box-binding proteins (TBP, 1:1000, ab300656, Abcam, Cambridge, UK); (3) rabbit monoclonal antibodies to voltage-dependent anion-selective channel 1 (VDAC1)/porin (1:1000, ab306581, Abcam, Cambridge, UK). Horseradish peroxidase-conjugated goat anti-rabbit (7074, Cell Signaling Technology, Danvers, MA, USA) or goat anti-mouse (AP130P, Sigma-Aldrich, Saint Louis, MO, USA) secondary antibodies were used at 1:200 and 1:1000 dilution, respectively. Incubation with the antibodies was performed as previously described [17]. Chemiluminescent detection was performed using SuperSignal West Pico PLUS chemiluminescent substrate (34580, Thermo Fisher Scientific, Waltham, MA, USA) and a C-DiGit blot scanner (LI-COR Biosciences, Linkoln, NE, USA) in a high-sensitivity mode (12 min scanning).

Target verification of proteomic profiling results in HCAEC was performed by fluorescent Western blotting as in [63]. Chameleon Duo Pre-Stained Protein Ladder (928–60,000, LI-COR Biosciences, Lincoln, NE, USA) was used as a molecular weight marker. Protein separation and transfer were conducted by sodium dodecyl sulfate–polyacrylamide gel electrophoresis (SDS-PAGE) and a dry blotting system (iBlot 2 Gel Transfer Device, Thermo Fisher Scientific, Waltham, MA, USA), using nitrocellulose transfer stacks (IB23001, Thermo Fisher Scientific, Waltham, MA, USA) as previously described [63]. Blocking of non-specific binding was carried out by incubation of nitrocellulose membranes in protein-free Block’n’Boost! solution (K-028, Molecular Wings, Kemerovo, Russia) for 1 h. The blots were probed with (1) rabbit antibodies to CD31 (1:2000 dilution, NB100-2284, Novus Biologicals, Centennial, CO, USA) and mouse antibodies to caspase 3 (1:250, ab208161, Abcam, Cambridge, UK); (2) rabbit antibodies to ERG transcription factor (1:500, ab92513, Abcam, Cambridge, UK); (3) mouse antibodies to endothelial nitric oxide synthase (1:500, SLM-33176M, Sunlong Biotech, Hangzhou, China); (4) mouse antibodies to glyceraldehyde 3-phosphate dehydrogenase (GAPDH, 1:500, SLM-33033M, Sunlong Biotech, Hangzhou, China). IRDye 680RD-conjugated goat anti-rabbit (926-68071, LI-COR Biosciences, Lincoln, NE, USA) and IRDye 800CW-conjugated goat anti-mouse (926-32210, LI-COR Biosciences, Lincoln, NE, USA), or IRDye 680RD-conjugated goat anti-mouse (926-68070, LI-COR Biosciences, Lincoln, NE, USA) IgG secondary antibodies were used at a 1:1000 dilution. Incubation with the antibodies and fluorescent detection was performed using an Odyssey XF imaging system (LI-COR Biosciences, Lincoln, NE, USA) at a 700 nm channel (685 nm excitation and 730 nm emission) and 800 nm channel (785 nm excitation and 830 nm emission).

To screen the activity of biochemical pathways, 300 µg protein lysate of PBS-, CPP-P-, and CPP-S-treated HCAECs were profiled for the phosphokinase activity using the respective dot blotting kit (150 µg protein lysate per antibody array A and B, ARY003B, R&D Systems, Minneapolis, MN, USA) according to the manufacturer’s protocol. Chemiluminescence detection of dot blotting results was performed using an Odyssey XF imaging system (LI-COR Biosciences, Lincoln, NE, USA).

## 3. Results

To investigate the differences in the protein expression in distinct cellular compartments upon CPP internalisation by HCAEC and HITAEC, we carried out a bioinformatic filtration in relation to cytosol, nuclei, mitochondria, lysosomes, and ER of shotgun proteomics data, described by us to a lesser extent before [14]. Despite certain limitations (presence of the same protein in several compartments and context- and cell-specificity of protein localisation), this approach seems to be helpful to underlay general tendencies within various compartments. The rationale behind applying such bioinformatic filtration included: (1) need to perform synchronous and objective analysis of compartment-specific alterations in all organelles accumulating excessive Ca^2+^ that is released upon dissolution of CPPs in lysosomes (i.e., cytosol, nuclei, mitochondria, lysosome, and ER); (2) employment of unbiased high-throughput approach such as UHPLC-MS/MS with ion mobility that identified 1188 (HCAEC) and 1264 (HITAEC) cytosolic proteins, 834 (HCAEC) and 896 (HITAEC) nuclear proteins, 379 (HCAEC) and 388 (HITAEC) mitochondrial proteins, 79 (HCAEC) and 86 (HITAEC) lysosomal proteins, and 305 (HCAEC) and 306 (HITAEC) ER proteins, and therefore ensured analysis robustness to possible inconsistencies of protein intracellular localisation between cell types, post-translational modifications, and treatment conditions; (3) subsequent Western blotting verification of bioinformatic filtration results. Although there is a number of commercially available kits for cell fractionation, they are capable of separating either mitochondria, lysosomes, or ER, but not all these organelles simultaneously. In this paper, we aimed to conduct unbiased and synchronous screening for the molecular signatures of cellular compartment-specific endothelial response to CPPs.

In total, we have identified 3671 proteins in HCAEC and 3593 proteins in HITAEC. As expected, we observed a significant overlap of all groups with the cytoplasmic compartment and a considerably smaller overlap with other compartments: 534 (HCAEC) and 571 (HITAEC) proteins unique for cytosol, 245 (HCAEC) and 272 (HITAEC) proteins unique for nucleus, 259 (HCAEC) and 263 (HITAEC) proteins unique for mitochondria, 51 (HCAEC) and 56 (HITAEC) proteins unique for lysosomes, and 194 proteins (both for HCAEC and HITAEC) unique for ER. Respective Venn diagrams are presented in Supplementary Figure S2 (for HCAEC) and Supplementary Figure S3 (for HITAEC).

**Figure 1:**
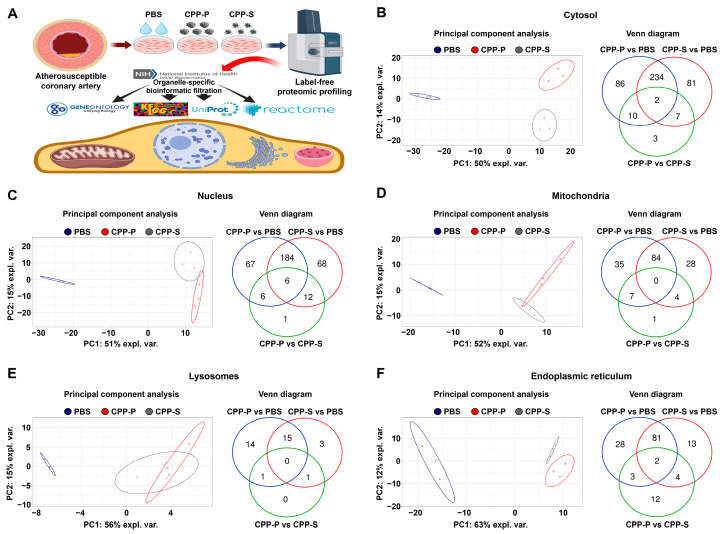
Experimental pipeline (**A**) and bioinformatic analysis of (**B**) cytosolic, (**C**) nuclear, (**D**) mitochondrial, (**E**) lysosomal, and (**F**) endoplasmic reticulum (ER) proteins in CPP-P- or CPP-S-treated human coronary artery endothelial cells (HCAEC) as compared to control (PBS-treated) cells. In each letter: principal component analysis (**left**) demonstrating the relative distance between PBS (sham, blue dots), CPP-P (red triangles), and CPP-S (gray crosses)-treated cells in relation to the proteome of each indicated compartment; Venn diagram (**right**) showing the number of differentially expressed as well as common proteins in CPP-P vs. PBS, CPP-S vs. PBS, and CPP-P vs. CPP-S comparisons for each of the organelles.

During the bioinformatic analysis of HCAEC (Figure 1A), principal component analysis showed a significant distance between PBS (sham)- and CPP-treated cells, whereas the clusters of CPP-P and CPP-S-treated cells were closely located (Figure 1B–F). Likewise, the number of differentially expressed proteins (DEPs) in CPP-P versus PBS and CPP-S versus PBS comparisons considerably exceeded the number of DEPs in CPP-P vs. CPP-S comparisons for each organelle (Figure 1B–F and Supplementary Figures S4–S13). In HCAEC, the total number of overexpressed and underexpressed DEPs upon CPP-P treatment was 97 and 235 (cytosol), 78 and 185 (nuclei), 62 and 64 (mitochondria), 20 and 10 (lysosomes), and 91 and 23 (ER), whilst at CPP-S treatment, it was 107 and 217 (cytosol), 76 and 194 (nuclei), 54 and 62 (mitochondria), 11 and 8 (lysosomes), and 75 and 24 (ER) (Table 1 and Figure 1B–F). As compared with CPP-P, the number of upregulated and downregulated DEPs upon CPP-S treatment was 14 and 8 (cytosol), 10 and 15 (nuclei), 8 and 4 (mitochondria), 0 and 2 (lysosomes), and 9 and 12 (ER) (Table 1 and Figure 1B–F).

**Figure 2:**
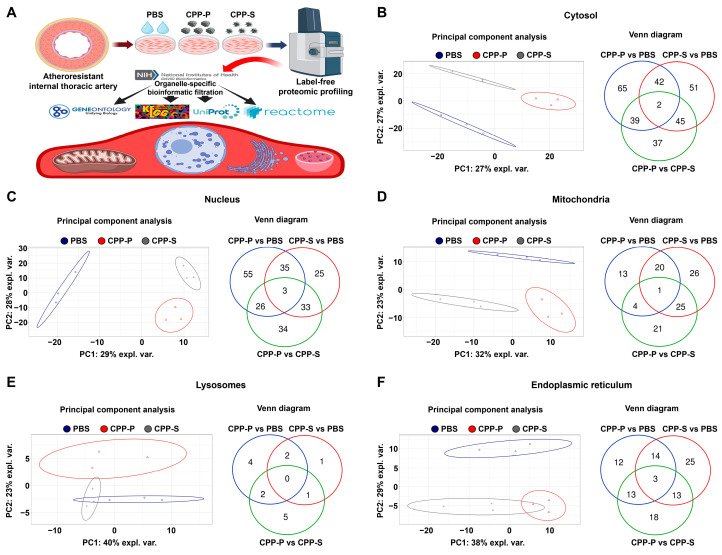
Experimental pipeline (**A**) and bioinformatic analysis of (**B**) cytosolic, (**C**) nuclear, (**D**) mitochondrial, (**E**) lysosomal, and (**F**) endoplasmic reticulum (ER) proteins in CPP-P- or CPP-S-treated human internal thoracic artery endothelial cells (HITAEC) as compared to control (PBS-treated) cells. In each letter: principal component analysis (**left**) demonstrating the relative distance between PBS (sham, blue dots), CPP-P (red triangles), and CPP-S (gray crosses)-treated cells in relation to the proteome of each indicated compartment; Venn diagram (**right**) showing the number of differentially expressed as well as common proteins in CPP-P vs. PBS, CPP-S vs. PBS, and CPP-P vs. CPP-S comparisons for each of the organelles.

In accord with the indicated findings, bioinformatic analysis of HITAEC (Figure 2A) showed clusterisation of PBS- and CPP-treated cells in terms of their cytosolic, nuclear, mitochondrial, and ER protein expression patterns, although lysosomal proteomic signatures of PBS- and CPP-treated cells were not identified (Figure 2B–F). The number of DEPs in CPP-P versus PBS and CPP-S versus PBS comparisons exceeded the number of DEPs in CPP-P versus CPP-S comparisons in relation to each organelle, similar to HCAEC (Figure 2B–F and Supplementary Figures S14–S23). In HITAEC, the total number of overexpressed and underexpressed DEPs upon CPP-P treatment was 39 and 109 (cytosol), 35 and 84 (nuclei), 20 and 18 (mitochondria), 7 and 1 (lysosomes), and 38 and 4 (ER), whereas at CPP-S treatment, it was 47 and 93 (cytosol), 30 and 66 (nuclei), 44 and 28 (mitochondria), 3 and 1 (lysosomes), and 35 and 20 (ER) (Table 1 and Figure 2B–F). As compared with CPP-P, the number of upregulated and downregulated DEPs upon CPP-S treatment was 66 and 57 (cytosol), 44 and 52 (nuclei), 33 and 18 (mitochondria), 2 and 6 (lysosomes), and 7 and 40 (ER) (Table 1 and Figure 2B–F). Hence, the differences between CPP-P- and CPP-S-treated HITAEC were remarkably higher than in HCAEC.

Further, we noted the differences in the ratio of proteins which have been upregulated to those which were downregulated in CPP-P and CPP-S-treated cells from cytosol and nuclei (from 0.39 to 0.49 in HCAEC and from 0.36 to 0.50 in HITAEC) through mitochondria (from 0.87 to 0.97 in HCAEC and from 1.11 to 1.57 in HITAEC) to lysosomes (from 1.37 to 2.00 in HCAEC and from 3.00 to 7.00 in HITAEC) and ER (from 3.12 to 3.85 in HCAEC and from 1.75 and 9.50 in HITAEC) (Table 1). Therefore, we suggested mitochondria, lysosomes, and ER as primary cellular compartments mediating the stress response after CPP-P or CPP-S internalisation, whilst cytosolic and nuclear response was largely similar and has been mainly focused on downregulation of biochemical pathways accountable for the maintenance of cellular homeostasis. Notwithstanding, both of these pathological patterns made a critical contribution to the development of endothelial dysfunction including pro-inflammatory activation, cytokine response, and apoptosis.

We then investigated cytosolic and nuclear pathways which become enriched upon the treatment of HCAEC and HITAEC with CPP-P or CPP-S. Both CPP-P and CPP-S triggered the upregulation of ubiquitination, cytokine- and chemokine-mediated signaling, angiogenesis, apoptosis, and other Ca^2+^-dependent events (Supplementary Tables S2–S11), and CPP-S specifically initiated the activation of TLR4 signaling and response to oxidative stress in atherosusceptible HCAEC (Supplementary Tables S3 and S8). Concurrently, we observed downregulation of RNA metabolism, transcription, translation, cell cycle, and cell–cell adhesion (Supplementary Tables S2–S11). Apoptotic pathways were overrepresented in CPP-S- as compared with CPP-P-treated HCAEC and HITAEC (Supplementary Table S6). Upregulated and downregulated pathways were concordant between HCAEC and HITAEC and between CPP-P and CPP-S groups (Supplementary Tables S2–S11). Differentially expressed cytosolic and nuclear pathways were coherent in HCAEC (Supplementary Tables S2, S3, S7 and S8) but nuclear response to CPP-P and CPP-S in HITAEC (Supplementary Tables S9–S11) was less pronounced than in HCAEC (Supplementary Tables S7 and S8), having been restricted to response to Ca^2+^ and the nuclear factor (NF)-κB transcription factor pathway.

Since internalisation of CPPs induces lysosome-dependent cell death which involves the release of free Ca^2+^ ions into the lysosomes upon the dissolution of CPPs, followed by translocation of excessive Ca^2+^ ions from the lysosomes into the cytosol, osmotic imbalance, cytoplasm acidification, uncurbed Ca^2+^ entry into the mitochondria, mitochondrial outer membrane permeabilisation, release of Ca^2+^ and pro-apoptotic proteins (such as cytochrome c, SMAC/DIABLO, and HtrA2/Omi) from the mitochondria to cytosol, apoptosome assembly, and initiation of intrinsic apoptosis pathways [64], we then focused on mitochondrial and lysosomal biochemical pathways upregulated upon CPP-P and CPP-S treatment (Table 2, Table 3, Table 4 and Table 5). The mitochondrial proteome of CPP-treated HCAEC and HITAEC has been enriched with molecular terms related to H^+^ and Ca^2+^ transmembrane transport, generation of reactive oxygen species, Ca^2+^ uptake, mitochondrial outer membrane permeabilisation, and regulated cell death (Table 2, Table 3 and Table 4). Whereas HCAEC showed equal response to CPP-P and CPP-S (Table 2 and Table 3), HITAEC vaguely reacted to CPP-P (Supplementary Table S12) but demonstrated exaggerated response to CPP-S, as apoptotic and particularly cytochrome c and SMAC/DIABLO-related pathways including apoptosome formation were significantly overrepresented in mitochondria-related proteins in CPP-S-treated cells (Table 4 and Table 5). Among the downregulated pathways were mitochondrial translation, biosynthesis of amino acids, fatty acid oxidation, pyruvate dehydrogenase activity, and energy generation systems such as citric acid cycle (Table 2, Table 3, Table 4 and Table 5).

In contrast to mitochondrial-related bioinformatic categories, differentially expressed lysosome-specific molecular terms upon the CPP-P or CPP-S treatment were scarce and were limited to hydrolase and glycosidase activity in HCAEC (Supplementary Tables S13 and S14) and to lysosomal or vacuolar acidification and H^+^ transmembrane transport in CPP-P-treated HITAEC (Table 6). This suggests immediate lysosomal response to CPP internalisation, which does not require alterations in transcriptional or translational programs and relies on existing protein machinery, also implying delayed cytosolic, mitochondrial, and nuclear response to calcium stress.

Considering significant changes in proteomes of different organelles caused by CPP internalisation, we have also investigated ER-specific biochemical pathways, as ER stress involves unfolded protein response which results in accumulation of unfolded or misfolded proteins in the ER lumen and in programmed cell death if not corrected by halting the translation, misfolded protein degradation, or increased chaperone production [65,66,67]. Both HCAEC and HITAEC treated with CPP-P or CPP-S demonstrated molecular signatures of response to oxidative, calcium, and ER stress, unfolded protein binding, ubiquitination, and apoptosis (Table 7, Table 8, Table 9 and Table 10). In addition, HITAEC also showed protein signatures of endosomal, lysosomal, and vacuolar acidification as well as intracellular pH reduction specifically after CPP-P internalisation (Table 9). In keeping with these findings, response to calcium and ER stress was evident in CPP-P-treated HITAEC at direct comparison with those which were co-incubated with CPP-S (Supplementary Table S15).

**Figure 3:**
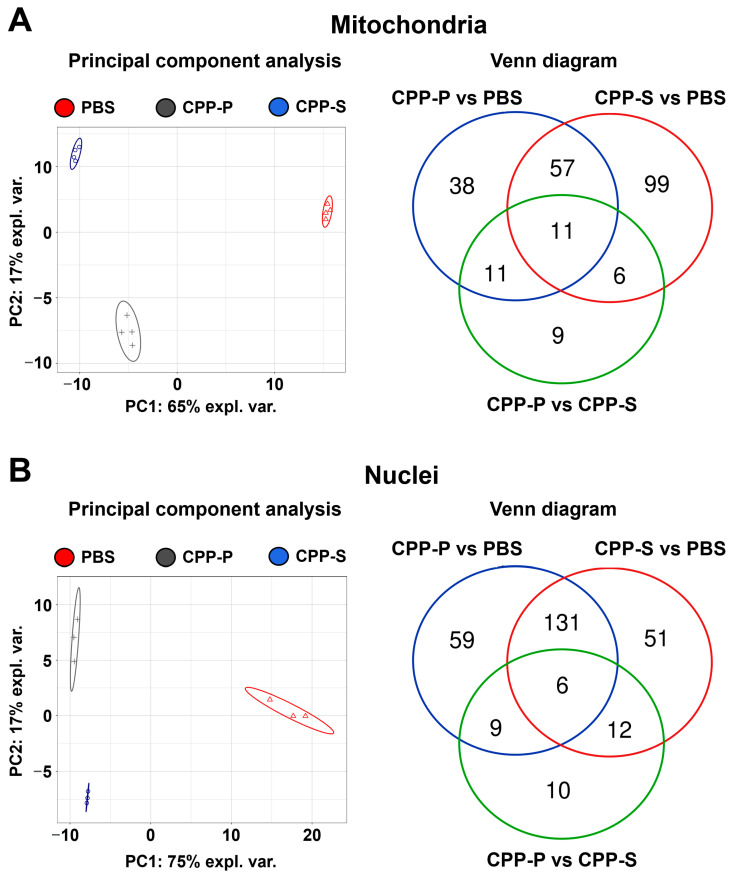
Bioinformatic analysis of mitochondrial (**A**) and nuclear (**B**) lysate in CPP-P- or CPP-S-treated human coronary artery endothelial cells (HCAEC) as compared to control (PBS-treated) cells. In each letter: principal component analysis (**left**) demonstrating the relative distance between PBS (sham, red triangles), CPP-P (gray crosses), and CPP-S (blue dots)-treated cells in relation to the proteome of each indicated compartment; Venn diagram (**right**) showing the number of differentially expressed as well as common proteins in CPP-P vs. PBS, CPP-S vs. PBS, and CPP-P vs. CPP-S comparisons for each of the organelles.

To verify the results of bioinformatic filtration of the organelle-specific proteome, we conducted a fractionation of PBS-, CPP-P, or CPP-S-treated HCAEC by selective lysis of mitochondrial and nuclear compartments accompanied by the extraction of compartment-specific proteins. The fractionation procedure has been verified by Western blotting for nuclear loading controls (proliferating cell nuclear antigen and TATA-box binding protein, Supplementary Figure S24A,B) and mitochondrial loading control (voltage-dependent anion-selective channel 1/porin, Supplementary Figure S24C). In total, UHPLC-MS/MS analysis identified 1942 mitochondrial proteins and 764 nuclear proteins. In keeping with the abovementioned results, clusters of PBS-, CPP-P, and CPP-S-treated cells were separated from each other with regards to both mitochondrial and nuclear proteomic profiles (Figure 3A,B). The number of upregulated and downregulated DEPs upon CPP-P treatment was 68 and 49 in mitochondria and 160 and 45 in nuclei, whereas after CPP-S treatment, these numbers reached 52 and 123 in mitochondria and 144 and 56 in nuclei (Figure 3A,B, Supplementary Figures S25 and S26). In comparison with CPP-P, the number of upregulated and downregulated DEPs upon CPP-S treatment was 3 and 34 in mitochondria and 9 and 28 in nuclei (Figure 3A,B, Supplementary Figures S27 and S28).

Pathway enrichment analysis found upregulation of cellular response to stress, cellular senescence induced by oxidative and telomere stress, senescence-associated secretory phenotype, Ca^2+^ binding, and programmed cell death in mitochondria upon CPP-P exposure (Table 11). Correspondingly, cellular response to chemical stress, NF-κB signaling pathway, and Ca^2+^ binding have been overexpressed in mitochondria after CPP-S treatment concurrently with downregulation of protein folding, cell redox homeostasis, and energy generation (Table 12). Comparison of mitochondrial biochemical pathways in CPP-P- and CPP-S-treated HCAEC revealed that similar pathways are overrepresented when comparing pathological effects of CPP-P either on PBS or CPP-S background (Supplementary Table S16). Similar bioinformatic analysis of nuclear lysate showed upregulation of unfolded protein response and oxidative stress-induced cellular senescence after CPP-P treatment, whilst transcription, post-transcriptional regulation, DNA repair, and cell cycle were downregulated (Supplementary Table S17). Nuclear response to CPP-S treatment included upregulation of calcium stress response, unfolded protein response, and apoptosis along with downregulation of similar pathways as after CPP-P exposure (Supplementary Table S18). In line with mitochondrial reaction pattern, nuclear response to CPP-P was stronger than to CPP-S and included upregulation of oxidative- and telomere stress-induced senescence, senescence-associated secretory phenotype, and apoptosis, although the expression of DNA repair pathways was also pronounced (Supplementary Table S19).

**Figure 4:**
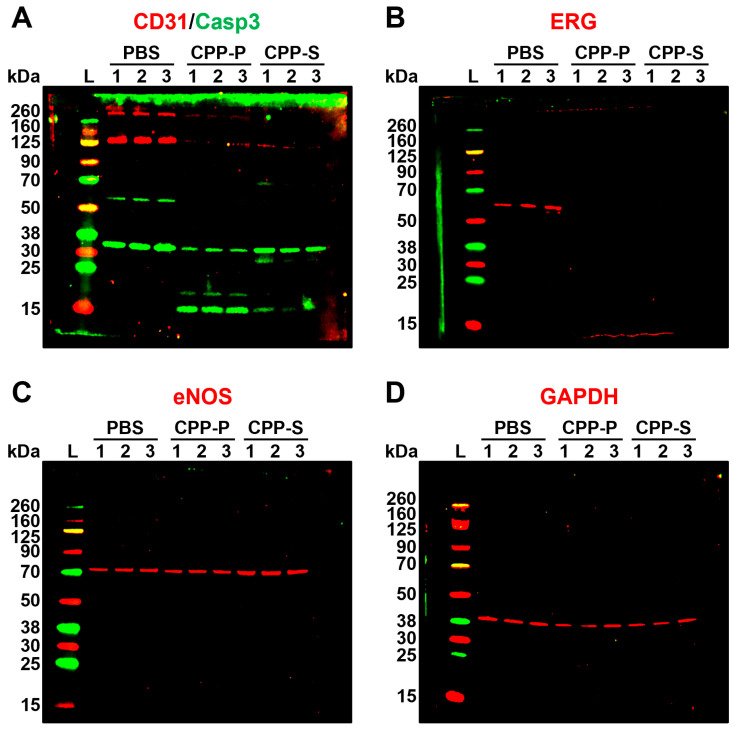
Western blotting for apoptosis and endothelial differentiation markers in PBS (sham)-, primary CPP (CPP-P), or secondary CPP (CPP-S)-treated primary human coronary artery endothelial cells (HCAEC). (**A**) CD31 (endothelial-specific transmembrane glycoprotein, red bands at ≈130 kDa), total and cleaved caspase-3 (an executioner caspase mediating apoptosis, green bands at ≈35 kDa for total caspase-3 and at ≈15 kDa for cleaved caspase-3); (**B**) ERG (endothelial-specific transcription factor, red bands at ≈60 kDa); (**C**) endothelial nitric oxide synthase (eNOS, an endothelial-specific enzyme catalysing the synthesis of nitric oxide, red bands at ≈73 kDa; (**D**) glyceraldehyde 3-phosphate dehydrogenase (GAPDH), a loading control, red bands at ≈40 kDa. Three samples per PBS, CPP-P, or CPP-S group have been measured. Note the significant fraction of cleaved caspase-3 in CPP-P and CPP-S groups, loss of CD31 glycoprotein receptor and ERG transcription factor in CPP-P- and CPP-S treated HCAEC, and equal eNOS synthesis. Shown are uncropped blots.

To verify the proteomic profiling results in regards to key endothelial signaling pathways, we performed Western blotting for the cleaved caspase-3 (a marker of apoptosis) and proteins indicating endothelial-to-mesenchymal transition, a common consequence of calcium, oxidative, and ER stress [17,68,69,70]. Evidently, cleaved caspase-3 was significantly increased in CPP-P- and CPP-S-treated in comparison with PBS-treated HCAEC, indicative of regulated cell death upon CPP treatment (Figure 4A). Specific endothelial transmembrane glycoprotein CD31 and pan-endothelial transcription factor ERG were downregulated after the incubation with CPP-P or CPP-S, showing the loss of canonical endothelial phenotype (Figure 4A,B). Expression of endothelial nitric oxide synthase (eNOS, Figure 4C) and a housekeeping metabolic enzyme glyceraldehyde 3-phosphate dehydrogenase (GAPDH, Figure 4D) was similar between all samples that confirmed partially retained endothelial function and equal protein loading.

**Figure 5:**
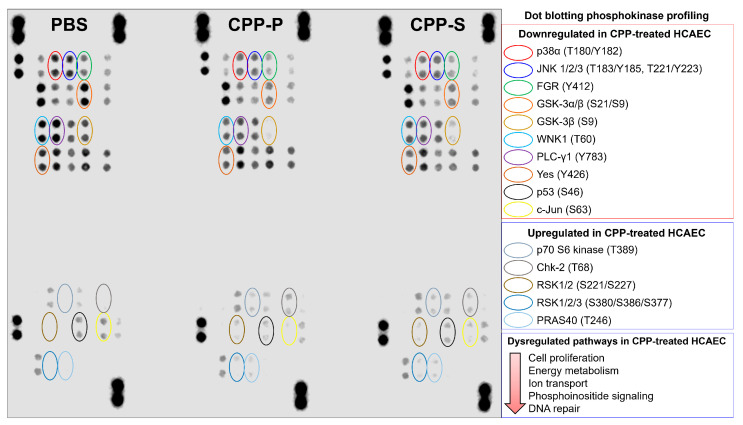
Dot blotting phosphokinase profiling in PBS (sham)-, primary CPP (CPP-P), or secondary CPP (CPP-S)-treated primary human coronary artery endothelial cells (HCAEC). Hypophosphorylated kinases: red: p38α (phosphorylated at T180/Y182 residues), dark blue: c-Jun N-terminal kinases (JNK) 1/2/3 (phosphorylated at T183/Y185 and/or T221/Y223 residues), green: Src-related FGR kinase (phosphorylated at Y412 residue), orange: glycogen synthase kinase (GSK)-3α/β (phosphorylated at S21/S9 residues), light gold: GSK-3β (phosphorylated at S9 residue), aquamarine: lysine deficient protein kinase (WNK) 1 (phosphorylated at T60 residue), violet: phospholipase C gamma 1 (PLC-γ1, phosphorylated at Y783 residue), brown: Yes kinase (phosphorylated at Y426 residue), black: p53 kinase (phosphorylated at S46 residue), yellow: c-Jun kinase (phosphorylated at S63 residue). Hyperphosphorylated kinases: light gray: p70 S6 kinase (phosphorylated at T389 residue), dark gray: Chk-2 kinase (phosphorylated at T68 residue), dark gold: ribosomal S6 kinases (RSK) 1/2 (phosphorylated at S221/S227 residues), light blue: RSK1/2/3 (phosphorylated at S380/S386/S377 residues), fade blue: proline-rich Akt substrate of 40 kDa (PRAS40) kinase (phosphorylated at T246 residue).

Further, we carried out a dot blotting profiling to evaluate the activity of central biochemical pathways, represented by 37 phosphokinases. In keeping with the proteomic profiling findings, the majority of phosphokinases were downregulated at CPP-P- or CPP-S treatment (Figure 5). Among the hypophosphorylated kinases were p38α mitogen-activated protein kinase (MAPK14) and c-Jun N-terminal kinases (JNKs) belonging to the MAPK family [71,72,73,74,75], Src-related kinases FGR and Yes, which are responsible for cell proliferation and regulation of endothelial junctional plasticity [76,77,78], glycogen synthase kinase (GSK)-3α/β, a central enzyme involved in carbohydrate metabolism [79,80], lysine deficient protein kinase 1 (“With-no-lysine” kinase 1, WNK1) participating in ion transport and proliferation [81,82], phospholipase C gamma 1 (PLC-γ1) orchestrating phosphoinositide signaling and promoting proliferation [83,84], and p53 kinase, which is responsible for DNA damage repair [85,86] (Figure 5). Upregulated kinases included anti-apoptotic and pro-survival p70 S6 kinase and pro-inflammatory proline-rich Akt substrate of 40 kDa (PRAS40) kinase belonging to the mTOR pathway [87,88,89], mitogen-activated ribosomal S6 kinases (RSK) 1/2/3 [90,91,92], and Chk-2 kinase, which increases cell susceptibility to DNA damage upon being phosphorylated at DNA strand breaks [93,94,95] (Figure 5).

As vascular calcification is largely driven by the osteogenic transition of vascular smooth muscle cells (VSMCs), which have remarkable phenotypic plasticity, and co-incubation of VSMCs with CPPs has been reported to induce their pro-inflammatory activation and osteochondrogenic dedifferentiation [96,97], we have evaluated whether CPPs affect the gene expression profile in primary human coronary artery SMCs (HCASMC). In our study, treatment with CPP-P or CPP-S did not promote the production of pro-inflammatory cytokines or upregulate the expression of the corresponding receptors (Table 13). However, *ACTA2* and *SMTN*, two genes encoding major contractile proteins indicative of quiescent VSMC phenotype, have been significantly downregulated in CPP-P- and CPP-S-treated HCASMC (Table 13). Concurrently, *COL1A1* and *COL1A2* genes encoding pro-alpha1(I) and pro-alpha2(I) chains of type 1 collagen were upregulated in HCASMC upon CPP-P or CPP-S treatment (Table 13). Taken together, these findings suggested that CPP-S contributed to the phenotypic shifting of HCASMC but did not trigger their pro-inflammatory activation.

## 4. Discussion

A healthy endothelium copes with the calcium burden caused by internalisation of circulating CPPs, as most of these particles are recycled by monocytes and liver or spleen macrophages. Yet, as soon as cardiovascular risk factors diminish endothelial resilience [98,99,100], the endothelium becomes vulnerable to mineral stress which develops upon digestion of CPPs in the lysosomes due to the massive Ca^2+^ overload in cytosol and mitochondria [14,15,17,18,20]. The consequences of such calcium burden include oxidative stress, pro-inflammatory activation of ECs, and programmed cell death if disruption of cellular homeostasis becomes irreversible [14,15,17,18,20]. However, organelle-specific response to CPPs, in particular in the context of differential pathological effects between CPP-P- and CPP-S, has not been investigated hitherto. Here, we focused on CPP-related proteomic signatures in cytosol, nuclei, mitochondria, lysosomes, and ER. To fulfill this task, we have used the data obtained by a label-free proteomic profiling of CPP-P- and CPP-S-treated HCAEC (i.e., an EC line from an atherosusceptible artery) and HITAEC (i.e., an EC line from an atheroresistant artery) [14].

We carried out a bioinformatic filtration of organelle-specific proteomes and then performed a pathway enrichment analysis of observed-versus-expected differences (i.e., between the number of differentially expressed proteins identified in the experiment or their actual proportion in any specific molecular term and the corresponding theoretical number or proportion that would be expected in case if null hypothesis is true). While recognising the general limitations associated with the context-specific nature of protein localisation, we believe that our approach provides valuable insights into the pathological consequences of CPP internalisation. Since many proteins exhibit a restricted number of localisations, we assumed that our approach might be ambiguous for some proteins, yet still capable of discovering trends associated with changes in the expression of proteins from the same compartment. By analysing each sub-dataset separately, we aimed to capture nuanced changes associated with specific cellular compartments for further target experimental studies. Hence, our intent was not to predict absolute protein localisation but rather to explore differential expression patterns within specific cellular compartments, providing a more refined view of the proteomic landscape after CPP internalisation. To confirm the data from the bioinformatic enrichment, we next conducted fractionation of HCAEC-treated cells into the mitochondrial and nuclear lysates with their subsequent proteomic profiling.

Having employed such a workflow, we found the following cellular compartment-specific molecular signatures: (1) upregulation of H^+^ and Ca^2+^ transmembrane translocation, Ca^2+^ stress, generation of reactive oxygen species and oxidative stress, unfolded protein response, mitochondrial outer membrane permeabilisation, and intrinsic apoptosis pathways in mitochondria and ER proteomes; (2) upregulation of Ca^2+^-dependent events, oxidative and telomere stress, cytokine- and chemokine-mediated signaling, and programmed cell death pathways in cytosol and nuclear proteomes; (3) downregulation of mitochondrial translation, biosynthesis of amino acids, fatty acid oxidation, pyruvate dehydrogenase activity, redox homeostasis, and energy generation in mitochondrial proteome; (4) downregulation of transcription, post-transcriptional regulation, translation, DNA repair, and cell cycle in cytosolic and nuclear proteomes. Intriguingly, lysosomal response was limited to a few molecular terms, suggesting that it likely exploits pre-existing protein machinery, whilst cytosolic, nuclear, mitochondrial, and ER response to CPP-induced mineral stress probably follows primary lysosomal dysfunction. The results were concordant between different EC lines (i.e., HCAEC and HITAEC), between CPP-P and CPP-S treatment groups, and between the proteomes enriched through either bioinformatic filtration or experimental fractionation.

The lack of considerable differences in molecular response of HCAEC and HITAEC to CPPs can be explained by their relatively low extent of heterogeneity (250–300 differentially expressed proteins and absence of any specific protein markers) and synergistic interactions between differentially expressed protein groups [63]. These results corroborate our previous findings where HCAEC and HITAEC also demonstrated similar molecular response patterns to CPP-P and CPP-S [16,17], although HCAECs were more susceptible to CPP treatment in some gene expression profiling experiments [14,18]. Although the coronary artery is atherosusceptible and the internal thoracic artery is atheroresistant [42,43], both of them are muscular arteries having equivalent blood flow pattern and therefore function in a similar haemodynamic environment which largely defines the protein expression pattern [101,102,103]. We suggest that whilst EC response to CPPs might be donor-dependent, limited molecular heterogeneity between HCAEC and HITAEC provides a molecular basis for their similar response patterns to CPP treatment.

Although it has been hypothesised that CPP-P are relatively innocuous and CPP-S exhibit significantly more severe pathological effects [21,96], our studies [14,16,17,18] and others [12,13] did not confirm this hypothesis with regards to the pro-inflammatory effects of these particles. CPP-P and CPP-S have different distributions in the human body, as CPP-P are primarily internalised by liver sinusoidal endothelial cells [12,13] and CPP-S are mainly recycled by liver and spleen macrophages [11,12,13]. Although CPP-P and CPP-S induce distinct patterns of inflammatory response [12,13,14], both of these particle types provoke a notable release of pro-inflammatory cytokines into the milieu, accompanied by the respective changes in gene expression [14,16,17,18]. However, CPP-S showed significantly higher ability to induce calcification than CPP-P [21,96], therefore being considered as a possible culprit of vascular calcification [10,22]. Here, organelle-specific response to CPPs demonstrated relatively mild differences in regards to the particle type, although treatment of HCAEC with CPP-S activated TLR4 signaling and was specifically associated with oxidative stress in cytosol and nuclei. Further, the cytosolic proteome of HCAEC and HITAEC, as well as the mitochondrial proteome of HITAEC exposed to CPP-S, was enriched with apoptotic signatures, whilst the lysosomal and ER proteome of HITAEC showed molecular signatures of H^+^ transmembrane transport, endosomal, lysosomal and vacuolar acidification, and intracellular pH reduction after the treatment with CPP-P. Collectively, these data indicate that CPP-S have stronger apoptotic effects to HCAEC and HITAEC, whereas CPP-P have profound effects on intracellular pH that adhere to our previous results [14,16,17,18] and support the hypothesis on higher dissolution of CPP-P and higher toxicity of CPP-S [12,13].

The compartment-specific molecular signatures revealed in our study correspond to the previous studies, which have identified lysosomes and mitochondria as potential target organelles upon cell exposure to CPPs. Treatment of monocytes or macrophages with CPPs led to their internalisation via macropinocytosis through the upregulation of the calcium-sensing receptor (CaSR), and enhanced lysosomal activity, triggered the activation of NLRP3 inflammasome, and stimulated interleukin (IL)-1β release [104,105]. Here, we have also found upregulation of the NF-κB signaling pathway in mitochondrial fractions of HCAEC treated with CPP-S and in cytosolic and nuclear fractions of HITAEC upon CPP-P stimulation. Signaling by interleukins has been also upregulated in cytosol and nuclei of HCAEC treated with CPP-P and CPP-S, suggesting compartment-specific involvement of the NLRP3-NF-κB-interleukin axis. Another study showed that internalisation of CPPs by renal tubular cells disrupted lysosomal homeostasis, increased their susceptibility to oxidative stress, and altered plasma membrane composition by reducing cholesterol content [106]. Our studies showed the osmotic translocation of excessive Ca^2+^ ions from the lysosomes to cytosol during the dissolution of CPPs in the acidic lysosomal environment and subsequent caspase-mediated intrinsic apoptosis of ECs, together suggesting lysosome-dependent cell death as a typical cell death subroutine for the scenario where the cell is unable to cope with CPP-induced Ca^2+^ stress [14,15,18]. Further, it was demonstrated that CPP internalisation augmented mitochondria-derived superoxide generation, increased hydrogen peroxide synthesis, and promoted the formation of 3-nitrotyrosine protein adduct, pointing at both oxidative and nitrosative stress occurring after exorbitant production of reactive oxygen species and reactive nitrogen species in the ECs and vascular smooth muscle cells [19,96,97]. Likewise, internalisation of CPPs by macrophages led to an increase in inducible nitric oxide synthase (iNOS) and the production of intracellular 8-iso-PGF2α, an oxidative stress marker [107]. Yet, molecular signatures of ER, cytosolic, and nuclear response to CPPs have not been interrogated to date, substantiating the novelty of our study. Collectively, functional experiments performed in other studies [14,15,18,19,96,97,104,105,106,107] and proteomic profiling conducted in our investigation indicate lysosomes, mitochondria, and ER as primary organelles mediating the molecular response to CPP internalisation. Among the most significant consequences of CPP internalisation is oxidative stress, which is executed by reactive oxygen species which are generated by various sources including mitochondria, ER, peroxisomes, cytosol, and plasma membrane [108,109,110,111]. Here, we have detected the molecular signatures of oxidative stress in mitochondrial and ER proteomes, suggesting that these organelles generate the majority of reactive oxygen species after the exposure of HCAEC and HITAEC to CPPs.

Concurrently, we revealed the downregulation of multiple kinases promoting cell proliferation, such as mitogen-activated protein kinases MAPK14 (p38α) and JNKs or Src-related kinases FGR and Yes, upon the treatment of HCAEC with CPP-P and CPP-S. Further, metabolic kinases GSK-3α/β, which is responsible for energy generation, WNK1, regulating ion transport, and PLC-γ1, which is accountable for the phosphoinositide signaling, were also hypophosphorylated at CPP-P and CPP-S exposure, and it has been reported that GSK-3α/β, WNK1, and PLC-γ1 also enhance cell proliferation [79,82,83]. In addition, we found a notable dysregulation of DNA repair after incubation of HCAEC with CPPs, since p53 kinase was downregulated and Chk-2 kinase was hyperphoshorylated (which typically occurs at DNA strand breaks). Simultaneous upregulation of mitogen-activated RSK1/2/3 kinases and mTOR pathway kinases p70 S6 and PRAS40 might indicate a compensatory response which defines the level of endothelial resilience [98,99,100] or correspond to CPP-initiated inflammation, since the activation of the mTOR pathway is associated with numerous pro-inflammatory effects [112,113]. The findings of phosphokinase profiling were in concordance with proteomic profiling results where energy generation, translation, cell cycle, and DNA repair have been downregulated in nuclei and cytosol, whereas pro-inflammatory signaling categories were overrepresented or overexpressed.

Further, we have examined whether treatment with CPPs is able to affect the gene expression profile of VSMCs, as their osteogenic transition represents a leading mechanism in neointimal calcification observed in around 70% of atherosclerotic plaques [114,115,116]. Earlier, it was shown that CPPs promote the release of pro-inflammatory cytokines such as tumor necrosis factor alpha (TNF-α) by VSMCs [96] and induce their osteochondrogenic dedifferentiation [22,96,117,118]. The inhibition of CPP formation [118] or the removal of CPPs from the milieu [22] reduced or prevented osteochondrogenic dedifferentiation in VSMCs, although it remains debatable whether such a scenario occurs in vivo, as intravascular mineral deposition and microvasculopathy in fetuin-A-deficient mice have not been associated with osteochondrogenic dedifferentiation of VSMCs, rather being driven by thrombosis and fibrosis [119]. Yet, the addition of CPPs isolated from the serum of patients with end-stage renal disease to the serum of healthy blood donors triggered the osteogenic transition of intact VSMCs [22]. Here, we revealed that 24 h incubation of HCASMC with CPP-P or CPP-S diminished the expression of contractile markers (*ACTA2* and *SMTN*, which encode alpha smooth muscle actin and smoothelin, respectively) along with augmenting the expression of genes responsible for collagen chain synthesis (*COL1A1* and *COL1A2*). However, neither pro-inflammatory cytokines nor osteogenic transcription factors have been upregulated in CPP-P- or CPP-S-treated VSMCs, indicating distinct patterns of molecular response to CPPs in ECs and VSMCs. Moreover, no mechanisms whereby VSMCs can reach VSMCs in vivo have been proposed, and the pathological effects of CPPs on VSMCs are probably mediated by the pro-inflammatory activation of ECs and subsequent paracrine stimulation. In a previous study [120], we showed that CPPs might be internalised by microvascular ECs lining the *vasa vasorum* but did not find any electron microscopy evidence that CPPs can enter the blood vessel wall. Rather than bypassing the EC monolayer, CPPs are at least partially dissolved in arterial or microvascular ECs, thereby causing endothelial dysfunction manifested as a pro-inflammatory shift in cytokine release. Excessive release of pro-inflammatory cytokines by ECs (i.e., IL-6, IL-8, and monocyte chemoattractant protein 1/C-C motif ligand 2) might then induce pathological changes in underlying VSMCs or pericytes, provoking their phenotypic switch. Yet, it remains arguable whether VSMCs are capable of internalising CPPs in vivo.

We suggest that this proteomics-empowered study expands our understanding of the cellular compartment-specific response to CPPs and summarises previous investigations which highlighted the mechanisms of lysosomal and mitochondrial dysfunction upon CPP internalisation. Further, we underscore the role of ER stress in CPP-induced cellular pathology, as it evidently accompanies disruption of lysosomal and mitochondrial homeostasis. Whilst lysosomes, mitochondria, and ER primarily react by an upregulation of their specific stress-related molecular terms, an enrichment of cytosolic and nuclear proteomes with molecular signatures of downregulated housekeeping signaling pathways indicates the concurrent shutdown of the cellular homeostasis mechanism, eventually nullifying endothelial resilience and contributing to surpassing the “point of no return” on route to regulated cell death.

The advantages of our study include: (1) the application of an unbiased, high-throughput, and pathophysiologically relevant proteomic approach which is capable of uncovering molecular signatures of organelle-specific dysfunction; (2) experimental verification of the bioinformatic filtration findings from the conventional proteomic profiling; (3) extensive coverage of cytosolic, nuclear, mitochondrial, lysosomal, and ER proteomes; (4) involvement of two distinct EC lines (i.e., HCAEC from the atherosusceptible coronary artery and HITAEC from the atheroresistant internal thoracic artery) and two CPP types with ascending maturation and different shape (amorphous and spherical CPP-P and crystalline and spindle-shaped CPP-S). Here, we focused on applying the holistic approach rather than using reductionist techniques such as Western blotting and reverse transcription-quantitative polymerase chain reaction (RT-qPCR) because the latter have been widely employed in our previous papers [14,15,16,17,18,19,20]. Although this might be considered as a study shortcoming, we believe that while the mechanisms of CPP-mediated mineral stress in mitochondria and lysosomes have been described in detail [14,15,16,17,18,19,20,104,105,106,107], the pathway-oriented, cellular compartment-specific molecular basis of EC response to CPPs needed investigation and clarification by unbiased proteomic analysis. Physicochemical properties and features of artificial synthesis of CPPs have also been described elsewhere and therefore were out of scope of this work [15,18,121].

Further studies might be focused on the proteomic profiling of EC secretome after the CPP treatment and on analysing the proteome of ECs incubated with conditioned medium from CPP-treated ECs. Such conditioned medium does not include CPPs but is enriched with pro-inflammatory cytokines (i.e., interleukin-6, interleukin-8, and monocyte chemoattractant protein 1) that might activate the intact ECs, thus inducing endothelial activation and mimicking the paracrine effects of CPP internalisation. In addition, the investigation of the interactome between CPP-treated ECs and intact vascular smooth muscle cells is a promising research direction in light of the recent discoveries on EC-guided, context-specific mesenchymal cell differentiation [122,123,124]. Dysfunctional ECs might indirectly promote the development of vascular or valvular calcification (as CPPs are incapable of penetrating elastic laminae which delimitate vascular smooth muscle cell layers). In addition, the mechanisms of CPP-triggered ER stress warrant further investigation in light of the importance of the unfolded protein response for the development of endothelial dysfunction, a mandatory trigger of atherosclerosis [125,126,127]. The development of EC-specific gene panels to detect organelle-specific dysfunction might be among the next aims after designing the general tool for defining endothelial dysfunction in vitro [128,129], which has been successfully applied in our recent studies [14,18,130].

## 5. Conclusions

The upregulation of H^+^ and Ca^2+^ translocation, Ca^2+^ stress, generation of reactive oxygen species and oxidative stress, unfolded protein response, mitochondrial outer membrane permeabilisation, and intrinsic apoptosis pathways in mitochondria and ER proteomes, as well as overrepresentation of Ca^2+^-dependent events, oxidative and telomere stress, cytokine- and chemokine-mediated signaling, and regulated cell death molecular signatures in cytosol and nuclear proteomes provide a framework for organelle-specific response after the internalisation of CPP-P or CPP-S by the ECs. Concurrently, the downregulation of transcription, RNA metabolism, translation, and cell cycle in cytosolic and nuclear proteomes, as well as the shutdown of mitochondrial translation, biosynthesis of amino acids, fatty acid oxidation, pyruvate dehydrogenase activity, redox homeostasis, and energy generation in the mitochondrial proteome exert another critical contribution to EC response to CPP-mediated mineral stress.

## Figures and Tables

**Table 1:** Count of upregulated and downregulated differentially expressed proteins (DEPs) in PBS (sham)-, primary CPP (CPP-P), or secondary CPP (CPP-S)-treated primary human coronary artery endothelial cells (HCAEC) and human internal thoracic artery endothelial cells (HITAEC).

Cellular Compartment	Comparison	Upregulated or Downregulated upon CPP Treatment	Number of DEPs
HCAEC			
Cytosol	CPP-P vs. PBS	Upregulated	97
		Downregulated	235
		Ratio	0.41
	CPP-S vs. PBS	Upregulated	107
		Downregulated	217
		Ratio	0.49
	CPP-S vs. CPP-P	Upregulated	14
		Downregulated	8
		Ratio	1.75
Nuclei	CPP-P vs. PBS	Upregulated	78
		Downregulated	185
		Ratio	0.42
	CPP-S vs. PBS	Upregulated	76
		Downregulated	194
		Ratio	0.39
	CPP-S vs. CPP-P	Upregulated	10
		Downregulated	15
		Ratio	0.67
Mitochondria	CPP-P vs. PBS	Upregulated	62
		Downregulated	64
		Ratio	0.97
	CPP-S vs. PBS	Upregulated	54
		Downregulated	62
		Ratio	0.87
	CPP-S vs. CPP-P	Upregulated	8
		Downregulated	4
		Ratio	2.00
Lysosomes	CPP-P vs. PBS	Upregulated	20
		Downregulated	10
		Ratio	2.00
	CPP-S vs. PBS	Upregulated	11
		Downregulated	8
		Ratio	1.37
	CPP-S vs. CPP-P	Upregulated	0
		Downregulated	2
		Ratio	0.00
Endoplasmic reticulum	CPP-P vs. PBS	Upregulated	91
		Downregulated	23
		Ratio	3.96
	CPP-S vs. PBS	Upregulated	75
		Downregulated	24
		Ratio	3.12
	CPP-S vs. CPP-P	Upregulated	9
		Downregulated	12
		Ratio	0.75
HITAEC			
Cytosol	CPP-P vs. PBS	Upregulated	39
		Downregulated	109
		Ratio	0.36
	CPP-S vs. PBS	Upregulated	47
		Downregulated	93
			0.50
	CPP-S vs. CPP-P	Upregulated	66
		Downregulated	57
		Ratio	1.16
Nuclei	CPP-P vs. PBS	Upregulated	35
		Downregulated	84
		Ratio	0.42
	CPP-S vs. PBS	Upregulated	30
		Downregulated	66
		Ratio	0.45
	CPP-S vs. CPP-P	Upregulated	44
		Downregulated	52
		Ratio	0.85
Mitochondria	CPP-P vs. PBS	Upregulated	20
		Downregulated	18
		Ratio	1.11
	CPP-S vs. PBS	Upregulated	44
		Downregulated	28
		Ratio	1.57
	CPP-S vs. CPP-P	Upregulated	33
		Downregulated	18
		Ratio	1.83
Lysosomes	CPP-P vs. PBS	Upregulated	7
		Downregulated	1
		Ratio	7.00
	CPP-S vs. PBS	Upregulated	3
		Downregulated	1
		Ratio	3.00
	CPP-S vs. CPP-P	Upregulated	2
		Downregulated	6
		Ratio	0.33
Endoplasmic reticulum	CPP-P vs. PBS	Upregulated	38
		Downregulated	4
		Ratio	9.50
	CPP-S vs. PBS	Upregulated	35
		Downregulated	20
		Ratio	1.75
	CPP-S vs. CPP-P	Upregulated	7
		Downregulated	40
		Ratio	0.17

**Table 2:** Fold enrichment (observed-versus-expected) analysis of differentially expressed proteins in mitochondria of human coronary artery endothelial cells (HCAEC) treated with either primary CPPs (CPP-P) or control phosphate-buffer saline (PBS).

Molecular Term	Total Proteins	Percent from Differentially Expressed Proteins	Fold Enrichment	Adjusted *p* Value
Upregulated after CPP-P treatment				
Hydrogen ion transmembrane transport (GO BP)	11	17.5	24.3	1.0 × 10^−8^
Chemical carcinogenesis— reactive oxygen species (KEGG)	10	15.9	11.2	2.4 × 10^−6^
Calcium signaling pathway (KEGG)	5	7.9	4.9	1.3 × 10^−1^
Hydrogen ion transmembrane transporter activity (GO MF)	4	6.3	36.7	1.4 × 10^−2^
Metalloendopeptidase activity (GO MF)	4	6.3	11.5	1.7 × 10^−1^
Cellular senescence (KEGG)	4	6.3	6.4	1.6 × 10^−1^
Necroptosis (KEGG)	4	6.3	6.3	1.6 × 10^−1^
Vpr-mediated induction of apoptosis by mitochondrial outer membrane permeabilisation (Reactome)	3	4.8	243.5	1.3 × 10^−3^
Mitochondrial calcium ion transport (GO BP)	3	4.8	58.7	4.0 × 10^−2^
Metallopeptidase activity (GO MF)	3	4.8	13.4	2.1 × 10^−1^
Positive regulation of hydrogen peroxide biosynthetic process (GO BP)	2	3.2	104.4	3.0 × 10^−1^
Mitochondrial calcium uptake (GO BP)	2	3.2	62.6	3.9 × 10^−1^
Positive regulation of necrotic cell death (GO BP)	2	3.2	56.9	4.1 × 10^−1^
Regulation of mitochondrial membrane permeability (GO BP)	2	3.2	52.2	4.3 × 10^−1^
Mitochondrial calcium ion homeostasis(GO BP)	2	3.2	39.1	5.1 × 10^−1^
Downregulated after CPP-P treatment				
Mitochondrial translation elongation (Reactome)	25	39.1	57.2	1.6 × 10^−35^
Mitochondrial translation (Reactome)	25	39.1	53.6	5.0 × 10^−35^
Translation (Reactome)	25	39.1	17.1	9.4 × 10^−23^
Metabolism of proteins (Reactome)	25	39.1	2.5	1.1 × 10^−4^
Mitochondrial translation termination (Reactome)	23	35.9	52.7	6.3 × 10^−32^
Mitochondrial translation initiation (Reactome)	23	35.9	52.7	6.3 × 10^−32^
Oxidoreductase (UniProtKB Keywords)	11	17.2	4.2	1.2 × 10^−3^
The citric acid (TCA) cycle and respiratory electron transport (Reactome)	5	7.8	5.6	9.0 × 10^−2^
Biosynthesis of amino acids (KEGG)	4	6.2	13.7	5.8 × 10^−2^
NAD binding (GO MF)	3	4.7	26.2	1.5 × 10^−1^
Glutathione metabolic process (GO BP)	3	4.7	19.9	3.3 × 10^−1^
Detoxification of reactive oxygen species (Reactome)	3	4.7	16.2	1.1 × 10^−1^
Fatty acid metabolism (UniProtKB Keywords)	3	4.7	8.4	4.5 × 10^−1^
Acyl-CoA dehydrogenase activity (GO MF)	2	3.1	55.6	5.8 × 10^−1^
Protein import into mitochondrial outer membrane (GO BP)	2	3.1	45.5	7.1 × 10^−1^

**Table 3:** Fold enrichment (observed-versus-expected) analysis of differentially expressed proteins in mitochondria of human coronary artery endothelial cells (HCAEC) treated with either secondary CPPs (CPP-S) or control phosphate-buffer saline (PBS).

Molecular Term	Total Proteins	Percent from Differentially Expressed Proteins	Fold Enrichment	Adjusted *p* Value
Upregulated after CPP-S treatment				
Chemical carcinogenesis— reactive oxygen species (KEGG)	9	16.4	10.4	3.8 × 10^−5^
Hydrogen ion transmembrane transport (GO BP)	8	14.5	19.9	3.0 × 10^−5^
Calcium signaling pathway (KEGG)	5	9.1	5.1	1.2 × 10^−1^
Cellular senescence (KEGG)	4	7.3	6.6	1.5 × 10^−1^
Necroptosis (KEGG)	4	7.3	6.5	1.5 × 10^−1^
Vpr-mediated induction of apoptosis by mitochondrial outer membrane permeabilisation (Reactome)	3	5.5	254.8	1.3 × 10^−3^
Mitochondrial calcium ion transport (GO BP)	3	5.5	66.2	3.0 × 10^−2^
Hydrogen ion transmembrane transporter activity (GO BP)	3	5.5	30.6	1.5 × 10^−1^
Metalloendopeptidase activity (GO MF)	3	5.5	9.6	2.6 × 10^−1^
Positive regulation of hydrogen peroxide biosynthetic process (GO BP)	2	3.6	117.7	2.6 × 10^−1^
Mitochondrial calcium uptake (GO BP)	2	3.6	70.6	3.5 × 10^−1^
Positive regulation of necrotic cell death (GO BP)	2	3.6	64.2	3.5 × 10^−1^
Regulation of mitochondrial membrane permeability (GO BP)	2	3.6	58.8	3.7 × 10^−1^
Mitochondrial calcium ion homeostasis(GO BP)	2	3.6	44.1	4.4 × 10^−1^
Apoptotic mitochondrial changes (GO BP)	2	3.6	41.5	4.6 × 10^−1^
Downregulated after CPP-S treatment				
Metabolic pathways (KEGG)	18	29.0	3.1	1.6 × 10^−4^
Metabolism of proteins (Reactome)	18	29.0	1.8	1.1 × 10^−1^
Mitochondrial translation elongation (Reactome)	17	27.4	38.2	1.7 × 10^−19^
Mitochondrial translation termination (Reactome)	15	24.2	33.7	1.4 × 10^−16^
Mitochondrial translation initiation (Reactome)	15	24.2	33.7	1.4 × 10^−16^
The citric acid (TCA) cycle and respiratory electron transport (Reactome)	12	19.4	13.2	2.1 × 10^−8^
Oxidoreductase (UniProtKB Keywords)	10	16.1	3.9	6.8 × 10^−3^
Metabolism of amino acids and derivatives (Reactome)	7	11.3	3.6	9.3 × 10^−2^
Biosynthesis of amino acids (KEGG)	5	8.1	17.6	2.5 × 10^−3^
Pyruvate metabolism (KEGG)	3	4.8	16.9	1.3 × 10^−1^

**Table 4:** Fold enrichment (observed-versus-expected) analysis of differentially expressed proteins in mitochondria of human internal thoracic artery endothelial cells (HITAEC) treated with either secondary CPPs (CPP-S) or control phosphate-buffer saline (PBS).

Molecular Term	Total Proteins	Percent from Differentially Expressed Proteins	Fold Enrichment	Adjusted *p* Value
Upregulated after CPP-S treatment				
Chemical carcinogenesis—reactive oxygen species (KEGG)	10	22.7	14.1	2.2 × 10^−7^
Apoptosis (UniProtKB Keywords)	7	15.9	5.4	8.1 × 10^−3^
Cellular response to chemical stress (Reactome)	5	11.4	7.9	7.7 × 10^−3^
Positive regulation of apoptotic process (GO BP)	5	11.4	7.1	1.8 × 10^−1^
Intrinsic apoptotic signaling pathway (GO BP)	4	9.1	51.4	8.6 × 10^−3^
Detoxification of reactive oxygen species (Reactome)	3	6.8	25.4	1.3 × 10^−1^
Activation of cysteine-type endopeptidase activity involved in apoptotic process by cytochrome c (GO BP)	2	4.5	132.1	4.6 × 10^−1^
Release of apoptotic factors from the mitochondria (Reactome)	2	4.5	89.4	3.2 × 10^−1^
SMAC (DIABLO) binds to IAPs (Reactome)	2	4.5	89.4	3.2 × 10^−1^
SMAC(DIABLO)- mediated dissociation of IAP:caspase complexes (Reactome)	2	4.5	89.4	3.2 × 10^−1^
SMAC, XIAP-regulated apoptotic response (Reactome)	2	4.5	78.3	3.4 × 10^−1^
Formation of apoptosome (Reactome)	2	4.5	56.9	3.7 × 10^−1^
Regulation of the apoptosome activity (Reactome)	2	4.5	56.9	3.7 × 10^−1^
Intrinsic apoptotic signaling pathway in response to oxidative stress (GO BP)	2	4.5	54.4	7.3 × 10^−1^
Cytochrome c- mediated apoptotic response (Reactome)	2	4.5	48.2	4.2 × 10^−1^
Release of cytochrome c from mitochondria (GO BP)	2	4.5	40.2	8.0 × 10^−1^
Downregulated after CPP-S treatment				
Metabolism (Reactome)	17	60.7	3.4	1.9 × 10^−4^
Oxidoreductase (UniProtKB Keywords)	10	35.7	8.8	7.8 × 10^−6^
The citric acid (TCA) cycle and respiratory electron transport (Reactome)	6	21.4	14.2	3.3 × 10^−3^
Metabolism of amino acids and derivatives (Reactome)	5	17.9	5.6	1.6 × 10^−1^
Biosynthesis of amino acids (KEGG)	4	14.3	21.5	4.0 × 10^−2^
Mitochondrial translation elongation (Reactome)	4	14.3	19.4	3.5 × 10^−2^
Mitochondrial translation (Reactome)	4	14.3	18.1	3.5 × 10^−2^
Glucose metabolic process (GO BP)	3	10.7	32.5	1.1 × 10^−1^
Mitochondrial translation initiation (Reactome)	3	10.7	14.5	2.2 × 10^−1^
Mitochondrial translation termination (Reactome)	3	10.7	14.5	2.2 × 10^−1^
Respiratory electron transport (Reactome)	3	10.7	12.3	2.8 × 10^−1^
Pyruvate dehydrogenase (NAD+) activity (GO MF)	2	7.1	225.5	1.7 × 10^−1^
Acetyl-CoA biosynthetic process from pyruvate (GO BP)	2	7.1	198.1	1.8 × 10^−1^
Acyl-CoA dehydrogenase activity (GO MF)	2	7.1	123.0	2.6 × 10^−1^
Regulation of pyruvate dehydrogenase (PDH) complex (Reactome)	2	7.1	52.7	3.6 × 10^−1^

**Table 5:** Fold enrichment (observed-versus-expected) analysis of differentially expressed proteins in mitochondria of human internal thoracic artery endothelial cells (HITAEC) treated with either primary CPPs (CPP-P) or secondary CPPs (CPP-S).

Molecular Term	Total Proteins	Percent from Differentially Expressed Proteins	Fold Enrichment	Adjusted *p* Value
Upregulated after CPP-P treatment				
Metabolism (Reactome)	9	50.0	3.3	1.4 × 10^−1^
Respiratory electron transport, ATP synthesis by chemiosmotic coupling, and heat production by uncoupling proteins (Reactome)	3	16.7	18.5	7.0 × 10^−1^
The citric acid (TCA) cycle and respiratory electron transport (Reactome)	3	16.7	13.2	8.9 × 10^−1^
Calcium signaling pathway (KEGG)	3	16.7	10.0	1.0 × 10^−1^
ATP synthesis coupled proton transport (GO BP)	2	11.1	84.6	1.0 × 10^−1^
Upregulated after CPP-S treatment				
Apoptosis (UniProtKB Keywords)	5	15.2	5.8	3.9 × 10^−2^
Detoxification of reactive oxygen species (Reactome)	4	12.1	43.9	2.4 × 10^−3^
Cellular response to chemical stress (Reactome)	4	12.1	8.2	6.9 × 10^−2^
Chemical carcinogenesis—reactive oxygen species (KEGG)	4	12.1	6.9	1.4 × 10^−1^
Intrinsic apoptotic signaling pathway (GO BP)	3	9.1	53.9	8.2 × 10^−2^
Apoptosis (KEGG)	3	9.1	8.5	2.6 × 10^−1^
Activation of cysteine-type endopeptidase activity involved in apoptotic process by cytochrome c (GO BP)	2	6.1	184.9	2.8 × 10^−1^
SMAC (DIABLO) binds to IAPs (Reactome)	2	6.1	115.9	8.1 × 10^−2^
SMAC(DIABLO)- mediated dissociation of IAP:caspase complexes (Reactome)	2	6.1	115.9	8.1 × 10^−2^
Release of apoptotic factors from the mitochondria (Reactome)	2	6.1	115.9	8.1 × 10^−2^
SMAC, XIAP-regulated apoptotic response (Reactome)	2	6.1	101.5	8.8 × 10^−2^
Intrinsic apoptotic signaling pathway in response to oxidative stress (GO BP)	2	6.1	76.1	3.6 × 10^−1^
Regulation of the apoptosome activity (Reactome)	2	6.1	73.8	1.0 × 10^−1^
Formation of apoptosome (Reactome)	2	6.1	73.8	1.1 × 10^−1^
Cytochrome c- mediated apoptotic response (Reactome)	2	6.1	62.4	1.2 × 10^−1^

**Table 6:** Fold enrichment (observed-versus-expected) analysis of differentially expressed proteins in lysosomes of human internal thoracic artery endothelial cells (HITAEC) treated with either primary CPPs (CPP-P) or control phosphate-buffer saline (PBS).

Molecular Term	Total Proteins	Percent from Differentially Expressed Proteins	Fold Enrichment	Adjusted *p* Value
Upregulated after CPP-P treatment				
Innate immune system (Reactome)	4	57.1	8.2	6.2 × 10^−2^
Lysosomal lumen acidification (GO BP)	2	28.6	252.1	2.6 × 10^−1^
Vacuolar acidification (GO BP)	2	28.6	241.2	2.6 × 10^−1^
Catalytic activity (GO MF)	2	28.6	45.8	3.6 × 10^−1^
Hydrogen ion transmembrane transport (GO BP)	2	28.6	39.1	1.0 × 10^−1^
Downregulated after CPP-P treatment				
None				

**Table 7:** Fold enrichment (observed-versus-expected) analysis of differentially expressed proteins in endoplasmic reticulum of human coronary artery endothelial cells (HCAEC) treated with either primary CPPs (CPP-P) or control phosphate-buffer saline (PBS).

Molecular Term	Total Proteins	Percent from Differentially Expressed Proteins	Fold Enrichment	Adjusted *p* Value
Upregulated after CPP-P treatment				
Metabolism of proteins (Reactome)	20	22.0	1.7	2.8 × 10^−1^
Hydrolase (UniProtKB Keywords)	16	17.6	1.7	2.5 × 10^−1^
Protein processing in endoplasmic reticulum (KEGG)	15	16.5	13.3	1.8 × 10^−10^
ER to Golgi vesicle-mediated transport (GO BP)	8	8.8	12.6	1.4 × 10^−3^
Apoptosis (UniProtKB Keywords)	7	7.7	2.6	2.0 × 10^−1^
Response to endoplasmic reticulum stress (GO BP)	6	6.6	15.2	9.6 × 10^−3^
Unfolded protein binding (GO MF)	6	6.6	9.9	2.6 × 10^−2^
Peptidase activity (GO MF)	5	5.5	10.3	6.5 × 10^−2^
Phagosome (KEGG)	5	5.5	5.1	1.4 × 10^−1^
Endocytosis (GO BP)	5	5.5	4.9	5.0 × 10^−1^
Ubiquitin-transport endoplasmic-reticulum-associated protein degradation pathway (GO BP)	4	4.4	10.5	2.7 × 10^−1^
Response to oxidative stress (GO BP)	4	4.4	7.0	5.0 × 10^−1^
Response to elevated platelet cytosolic Ca^2+^ (Reactome)	4	4.4	5.0	5.0 × 10^−1^
Metalloexopeptidase activity (GO MF)	3	3.3	32.3	1.1 × 10^−1^
Cellular oxidant detoxification (GO BP)	3	3.3	8.6	6.8 × 10^−1^
Downregulated after CPP-P treatment				
Nuclear events mediated by NFE2L2 (Reactome)	3	13.0	23.1	8.3 × 10^−1^
KEAP1-NFE2L2 pathway (Reactome)	3	13.0	17.1	9.5 × 10^−1^
Glycerophospholipid biosynthesis (Reactome)	3	13.0	14.2	9.5 × 10^−1^
Phospholipid metabolism (Reactome)	3	13.0	8.6	1.0 × 10^−1^
Phospholipid transport (GO BP)	2	8.7	45.3	1.0 × 10^−1^

**Table 8:** Fold enrichment (observed-versus-expected) analysis of differentially expressed proteins in endoplasmic reticulum of human coronary artery endothelial cells (HCAEC) treated with either secondary CPPs (CPP-S) or control phosphate-buffer saline (PBS).

Molecular Term	Total Proteins	Percent from Differentially Expressed Proteins	Fold Enrichment	Adjusted *p* Value
Upregulated after CPP-S treatment				
Hydrolase (UniProtKB Keywords)	16	21.1	1.9	6.5 × 10^−2^
Protein processing in endoplasmic reticulum (KEGG)	11	14.5	12.7	3.7 × 10^−7^
ER to Golgi vesicle-mediated transport (GO BP)	8	10.5	15.1	5.3 × 10^−4^
Apoptosis (UniProtKB Keywords)	8	10.5	3.6	3.4 × 10^−2^
Unfolded protein binding (GO MF)	7	9.2	14.0	2.1 × 10^−3^
Intracellular protein transport (GO BP)	6	7.9	4.7	3.9 × 10^−1^
Response to endoplasmic reticulum stress (GO BP)	5	6.6	15.2	6.2 × 10^−2^
Peptidase activity (GO MF)	4	5.3	9.9	1.4 × 10^−1^
Response to oxidative stress (GO BP)	4	5.3	8.4	3.9 × 10^−1^
Response to elevated platelet cytosolic Ca^2+^ (Reactome)	4	5.3	6.5	5.8 × 10^−1^
Metalloexopeptidase activity (GO MF)	3	3.9	38.9	7.7 × 10^−2^
Retrograde vesicle-mediated transport, Golgi to ER (GO BP)	3	3.9	15.0	4.9 × 10^−1^
Endopeptidase activity (GO MF)	3	3.9	10.1	4.0 × 10^−1^
Ubiquitin-transport endoplasmic-reticulum-associated protein degradation pathway (GO BP)	3	3.9	9.5	6.5 × 10^−1^
ER-associated misfolded protein catabolic process (GO BP)	2	2.6	42.6	6.7 × 10^−1^
Downregulated after CPP-S treatment				
Metabolism of proteins (Reactome)	11	45.8	2.9	4.4 × 10^−2^
Post-translational protein modification (Reactome)	9	37.5	3.3	7.2 × 10^−2^
ER to Golgi vesicle-mediated transport (GO BP)	5	20.8	31.3	4.1 × 10^−3^
Transport to the Golgi and subsequent modification (Reactome)	5	20.8	14.1	1.5 × 10^−2^
Vesicle fusion with Golgi apparatus (GO BP)	2	8.3	187.6	6.7 × 10^−1^

**Table 9:** Fold enrichment (observed-versus-expected) analysis of differentially expressed proteins in the endoplasmic reticulum of human internal thoracic artery endothelial cells (HITAEC) treated with either primary CPPs (CPP-P) or control phosphate-buffer saline (PBS).

Molecular Term	Total Proteins	Percent from Differentially Expressed Proteins	Fold Enrichment	Adjusted *p* Value
Upregulated after CPP-P treatment				
Innate immune system (Reactome)	9	23.7	3.3	5.6 × 10^−1^
Apoptosis (UniProtKB Keywords)	7	18.4	4.8	8.2 × 10^−2^
Apoptotic process (GO BP)	6	15.8	5.0	1.0 × 10^−1^
Peptidase activity (GO MF)	4	10.5	20.0	1.4 × 10^−1^
Positive regulation of tumor necrosis factor production (GO BP)	3	7.9	14.5	1.0 × 10^−1^
Unfolded protein binding (GO MF)	3	7.9	12.1	8.7 × 10^−1^
Response to elevated platelet cytosolic Ca^2+^ (Reactome)	3	7.9	8.8	7.4 × 10^−1^
Golgi lumen acidification (GO BP)	2	5.3	102.2	1.0 × 10^−1^
Endosomal lumen acidification (GO BP)	2	5.3	73.0	1.0 × 10^−1^
Intracellular pH reduction (GO BP)	2	5.3	73.0	1.0 × 10^−1^
Metalloexopeptidase activity (GO MF)	2	5.3	52.6	8.7 × 10^−1^
Lysosomal lumen acidification (GO BP)	2	5.3	46.4	1.0 × 10^−1^
Vacuolar acidification (GO BP)	2	5.3	44.4	1.0 × 10^−1^
Downregulated after CPP-P treatment				
None				

**Table 10:** Fold enrichment (observed-versus-expected) analysis of differentially expressed proteins in the endoplasmic reticulum of human internal thoracic artery endothelial cells (HITAEC) treated with either secondary CPPs (CPP-S) or control phosphate-buffer saline (PBS).

Molecular Term	Total Proteins	Percent from Differentially Expressed Proteins	Fold Enrichment	Adjusted *p* Value
Upregulated after CPP-S treatment				
Unfolded protein binding (GO MF)	6	17.1	25.7	3.4 × 10^−4^
Calcium ion binding (GO MF)	6	17.1	4.5	1.8 × 10^−1^
Protein processing in endoplasmic reticulum (KEGG)	5	14.3	16.2	5.3 × 10^−3^
Response to elevated platelet cytosolic Ca^2+^ (Reactome)	4	11.4	15.6	1.6 × 10^−1^
Intracellular protein transport (GO BP)	4	11.4	7.4	8.8 × 10^−1^
Endoplasmic- reticulum-associated protein degradation pathway (GO BP)	3	8.6	91.0	2.2 × 10^−1^
Positive regulation of tumor necrosis factor production (GO BP)	3	8.6	17.2	8.8 × 10^−1^
ER to Golgi vesicle- mediated transport (GO BP)	3	8.6	13.5	9.2 × 10^−1^
Disorders oftransmembrane transporters (Reactome)	3	8.6	8.9	1.0 × 10^−1^
Regulation ofbeta-amyloid clearance (GO BP)	2	5.7	303.3	7.5 × 10^−1^
Downregulated after CPP-S treatment				
Lipid metabolism (UniProtKB Keywords)	6	30.0	7.0	1.3 × 10^−2^
Lipid biosynthesis (UniProtKB Keywords)	3	15.0	15.3	1.3 × 10^−1^
Metabolism of steroids (Reactome)	3	15.0	15.2	3.5 × 10^−1^
Cargo concentration in the endoplasmic reticulum (Reactome)	2	10.0	47.4	3.5 × 10^−1^
Steroid biosynthesis (UniProtKB Keywords)	2	10.0	39.4	2.2 × 10^−1^

**Table 11:** Fold enrichment (observed-versus-expected) analysis of differentially expressed proteins in mitochondrial lysate of human coronary artery endothelial cells (HCAEC) treated with either primary CPPs (CPP-P) or control phosphate-buffer saline (PBS).

Molecular Term	Total Proteins	Percent from Differentially Expressed Proteins	Fold Enrichment	Adjusted *p* Value
Upregulated after CPP-P treatment				
Cellular responses to stress (Reactome)	35	38.9	6.5	1.9 × 10^−19^
Cellular senescence (Reactome)	29	32.2	21.6	9.0 × 10^−30^
Diseases of programmed cell death (Reactome)	27	30.0	37.9	4.2 × 10^−34^
Oxidative stress- induced senescence (Reactome)	27	30.0	31.8	4.5 × 10^−32^
Senescence-associated secretory phenotype (Reactome)	19	21.1	35.5	2.4 × 10^−33^
DNA damage/Telomere stress-induced senescence (Reactome)	19	21.1	35.1	8.1 × 10^−23^
Depurination (Reactome)	17	18.9	45.2	3.3 × 10^−22^
Depyrimidination (Reactome)	17	18.9	41.4	1.5 × 10^−21^
Calcium ion binding (GO MF)	11	12.2	3.1	4.6 × 10^−2^
Angiogenesis (GO BP)	5	5.6	4.2	5.5 × 10^−1^
Calcium-dependent protein binding (GO MF)	4	4.4	9.3	1.2 × 10^−1^
Apoptotic execution phase (Reactome)	3	3.3	8.4	1.1 × 10^−1^
Downregulated after CPP-P treatment				
Chaperone (UniProtKB Keywords)	5	10.2	5.9	1.1 × 10^−1^

**Table 12:** Fold enrichment (observed-versus-expected) analysis of differentially expressed proteins in mitochondrial lysate of human coronary artery endothelial cells (HCAEC) treated with either secondary CPPs (CPP-S) or control phosphate-buffer saline (PBS).

Molecular Term	Total Proteins	Percent from Differentially Expressed Proteins	Fold Enrichment	Adjusted *p* Value
Upregulated after CPP-S treatment				
Transport of small molecules (Reactome)	10	19.2	3.8	1.4 × 10^−1^
Calcium ion binding (GO MF)	6	11.5	3.0	6.9 × 10^−1^
Angiogenesis (GO BP)	5	9.6	7.5	2.8 × 10^−1^
Ferroptosis (GO MF)	4	7.7	25.8	4.5 × 10^−2^
Positive regulation of I-κB kinase/NF-κB signaling (GO BP)	4	7.7	7.7	7.8 × 10^−1^
Fluid shear stress and atherosclerosis (KEGG)	4	7.7	7.6	7.2 × 10^−1^
Cellular response to chemical stress (Reactome)	4	7.7	5.5	1.8 × 10^−1^
Positive regulation of cysteine-type endopeptidase activity involved in apoptotic process (GO BP)	3	5.8	22.4	4.6 × 10^−1^
Low-density lipoprotein particle clearance (GO BP)	2	3.8	54.4	9.9 × 10^−1^
Metal ion transmembrane transporter activity (GO MF)	2	3.8	53.1	6.4 × 10^−1^
Downregulated after CPP-S treatment				
Cellular response to hypoxia (Reactome)	8	6.5	11.0	3.4 × 10^−5^
The citric acid (TCA) cycle and respiratory electron transport (Reactome)	7	5.7	4.1	1.7 × 10^−2^
Chaperone (UniProtKB Keywords)	7	5.7	3.7	5.9 × 10^−2^
Chaperone binding (GO MF)	4	3.3	5.7	4.4 × 10^−1^
Chaperone-mediated protein folding (GO BP)	3	2.4	12.3	3.4 × 10^−1^
Cell redox homeostasis (GO BP)	3	2.4	11.2	3.9 × 10^−1^
Negative regulation of cysteine-type endopeptidase activity involved in apoptotic process (GO BP)	3	2.4	8.6	5.8 × 10^−1^

**Table 13:** Gene expression analysis of human coronary artery smooth muscle cells (HCASMC) treated with either primary CPPs (CPP-P) or secondary CPPs (CPP-S). Reverse transcription-quantitative polymerase chain reaction measurements.

Gene		PBS-Treated HCASMC	CPP-P-Treated HCASMC	CPP-S-Treated HCASMC
Pro-inflammatory activation				
*IL1R1*	ΔCt	0.0275	0.0229	0.0317
	Fold change	1	0.83	1.15
*TNFRSF1A*	ΔCt	0.1356	0.1731	0.1791
	Fold change	1	1.28	1.32
*TNFRSF1B*	ΔCt	0.0099	0.0097	0.0124
	Fold change	1	0.98	1.25
*IL1B*	ΔCt	0.7671	0.9526	1.0805
	Fold change	1	1.24	1.41
*IL6*	ΔCt	0.1186	0.0324	0.0234
	Fold change	1	0.27	0.20
*CXCL8*	ΔCt	0.3979	0.2947	0.2249
	Fold change	1	0.74	0.57
*CCL2*	ΔCt	0.4915	0.2971	0.2220
	Fold change	1	0.60	0.45
Phenotypic plasticity markers				
*ACTA2*	ΔCt	0.1188	0.0371	0.0568
	Fold change	1	0.31	0.48
*SMTN*	ΔCt	0.0165	0.0053	0.0083
	Fold change	1	0.32	0.50
*VIM*	ΔCt	4.0615	3.5062	3.7011
	Fold change	1	0.86	0.91
*COL1A1*	ΔCt	0.3687	0.9190	0.6955
	Fold change	1	2.49	1.89
*COL1A2*	ΔCt	2.6047	3.9475	3.7623
	Fold change	1	1.52	1.44
*COL4A1*	ΔCt	0.2430	0.0761	0.1158
	Fold change	1	0.31	0.48
*MMP2*	ΔCt	0.2939	0.2441	0.2764
	Fold change	1	0.83	0.94
*RUNX2*	ΔCt	0.0203	0.0213	0.0196
	Fold change	1	1.05	0.97
*SOX9*	ΔCt	0.0167	0.0068	0.0081
	Fold change	1	0.41	0.49

## Data Availability

The mass spectrometry proteomics data are available from ProteomeXchange Consortium via the PRIDE partner repository with the dataset identifier PXD047581. Other data presented in this study are available in Supplementary Datafiles S1–S32 or on request from the corresponding author.

## Supplementary Materials

The following supporting information can be downloaded at: https://www.mdpi.com/article/10.3390/jcdd11010005/s1, Supplementary Tables S1–S19; Supplementary Figures S1–S28; Supplementary Datafiles S1–S32.
