# Supplementary material for: Calciprotein Particles Induce Cellular Compartment-Specific Proteome Alterations in Human Arterial Endothelial Cells
Source: J Cardiovasc Dev Dis. 2023 Dec 22;11(1):5. doi: 10.3390/jcdd11010005 (PMC10816121; doi:10.3390/jcdd11010005)

Supplementary Figure S1. Electron microscopy visualisation of primary calciprotein particles (CPP-P, left) and secondary calciprotein particles (CPP-S, right). Top: scanning electron microscopy. Bottom: transmission electron microscopy. Note the absence of mature CPPs (i.e., CPP-S) in immature CPP preparations (i.e., CPP-P).

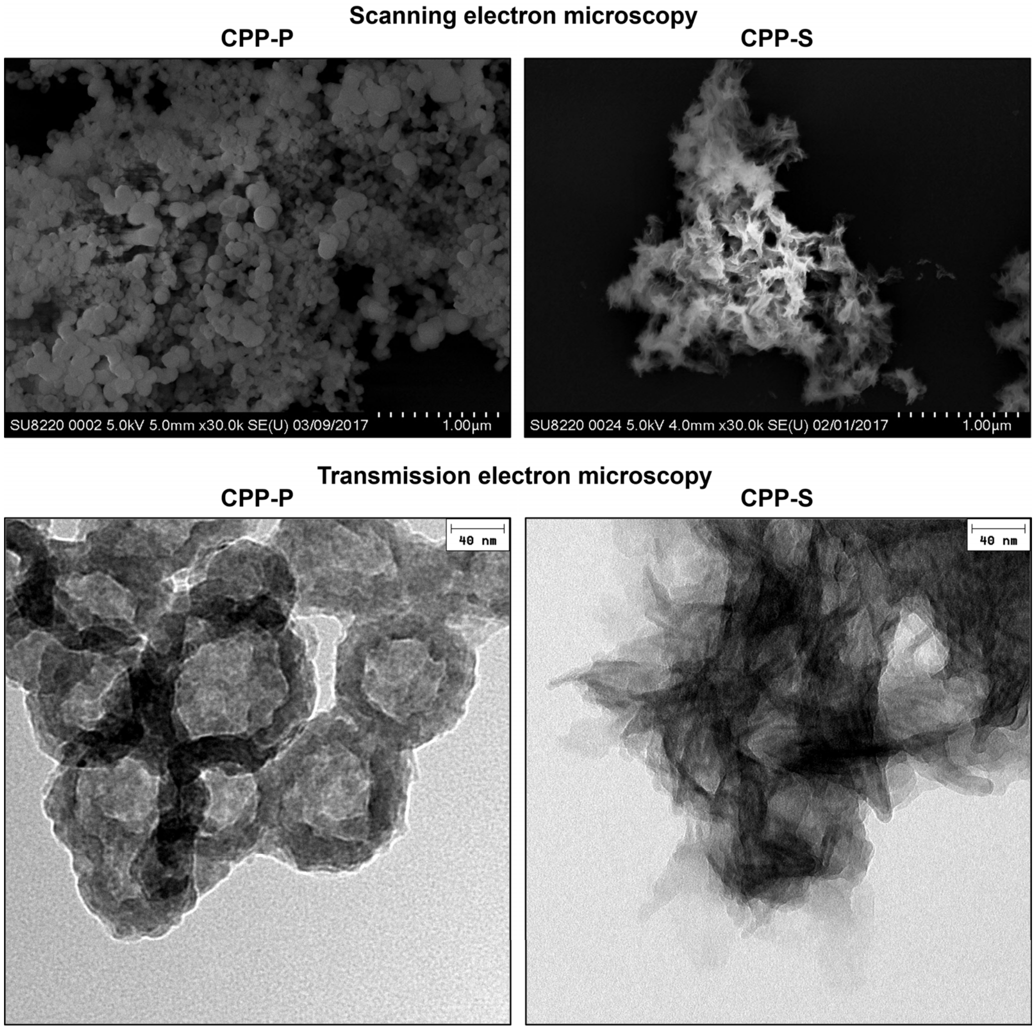

Supplementary Figure S2. Venn diagram indicating an overlap of the indicated proteins between the cellular compartments (cytosol, nucleus, mitochondria, lysosomes, and endoplasmic reticulum) and organelle-specific proteins in human coronary artery endothelial cells (HCAEC) upon the bioinformatic filtration.

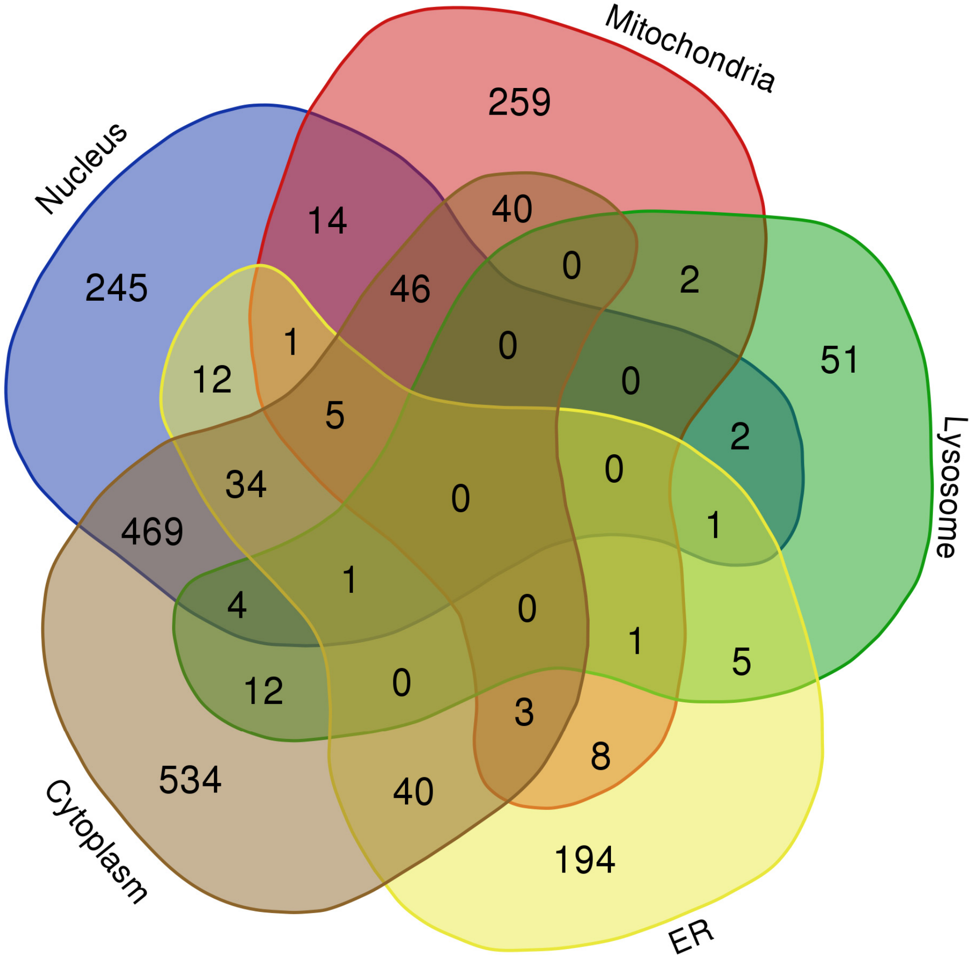

Supplementary Figure S3. Venn diagram indicating an overlap of the indicated proteins between the cellular compartments (cytosol, nucleus, mitochondria, lysosomes, and endoplasmic reticulum) and organelle-specific proteins in human internal thoracic artery endothelial cells (HITAEC) upon the bioinformatic filtration.

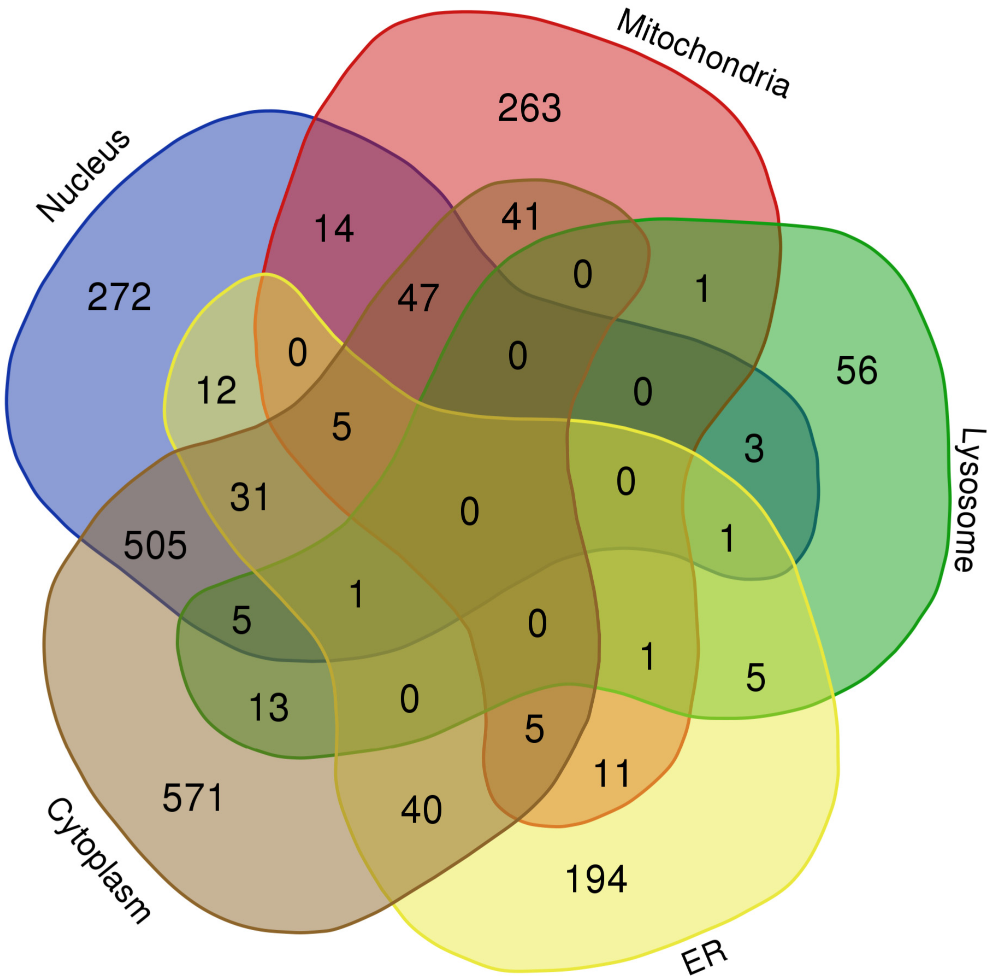

Supplementary Figure S4. Bioinformatic analysis of cytosolic proteins in CPP-P- and CPP-S-treated human coronary artery endothelial cells (HCAEC) as compared to control (PBS-treated) cells, presented as volcano plots. Gray points depict the proteins with $\log_2$ fold change < 1 and FDR-corrected p value > 0.05, green points depict the proteins with $\log_2$ fold change > 1 and FDR-corrected p value > 0.05, blue points depict the proteins with $\log_2$ fold change < 1 and FDR-corrected p value < 0.05, and red points depict the proteins with $\log_2$ fold change > 1 and FDR-corrected p value < 0.05 (termed as differentially expressed proteins) in each of the indicated organelles.

Cytosol

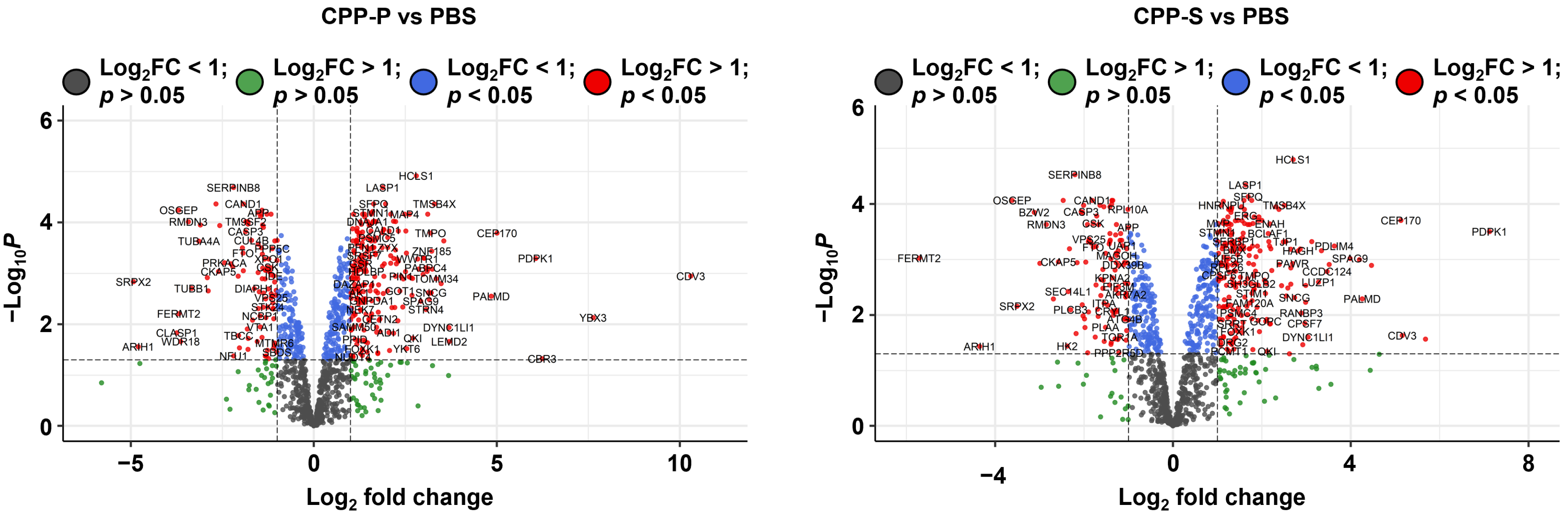

Supplementary Figure S5. Bioinformatic analysis of nuclear proteins in CPP-P- and CPP-S-treated human coronary artery endothelial cells (HCAEC) as compared to control (PBS-treated) cells, presented as volcano plots. Gray points depict the proteins with $\log_2$ fold change < 1 and FDR-corrected p value > 0.05, green points depict the proteins with $\log_2$ fold change > 1 and FDR-corrected p value > 0.05, blue points depict the proteins with $\log_2$ fold change < 1 and FDR-corrected p value < 0.05, and red points depict the proteins with $\log_2$ fold change > 1 and FDR-corrected p value < 0.05 (termed as differentially expressed proteins) in each of the indicated organelles.

Nuclei

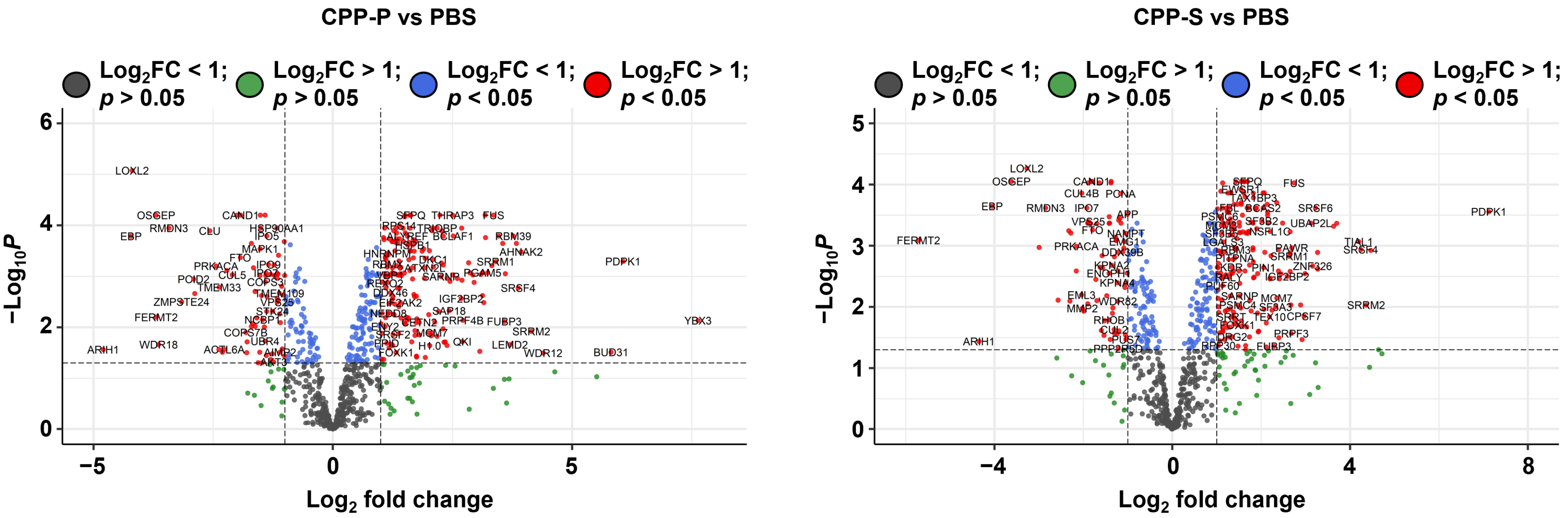

Supplementary Figure S6. Bioinformatic analysis of mitochondrial proteins in CPP-P- and CPP-S-treated human coronary artery endothelial cells (HCAEC) as compared to control (PBS-treated) cells, presented as volcano plots. Gray points depict the proteins with $\log_2$ fold change < 1 and FDR-corrected *p* value > 0.05, green points depict the proteins with $\log_2$ fold change > 1 and FDR-corrected *p* value > 0.05, blue points depict the proteins with $\log_2$ fold change < 1 and FDR-corrected *p* value < 0.05, and red points depict the proteins with $\log_2$ fold change > 1 and FDR-corrected *p* value < 0.05 (termed as differentially expressed proteins) in each of the indicated organelles.

Mitochondria

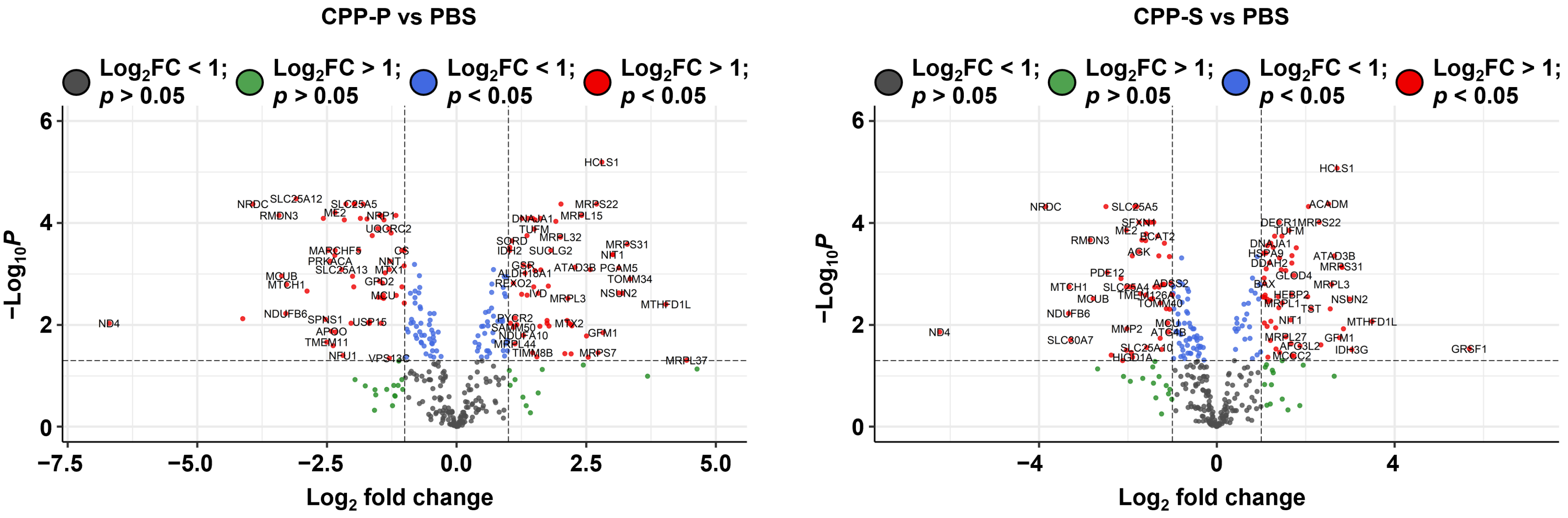

Supplementary Figure S7. Bioinformatic analysis of lysosomal proteins in CPP-P- and CPP-S-treated human coronary artery endothelial cells (HCAEC) as compared to control (PBS-treated) cells, presented as volcano plots. Gray points depict the proteins with $\log_2$ fold change < 1 and FDR-corrected *p* value > 0.05, green points depict the proteins with $\log_2$ fold change > 1 and FDR-corrected *p* value > 0.05, blue points depict the proteins with $\log_2$ fold change < 1 and FDR-corrected *p* value < 0.05, and red points depict the proteins with $\log_2$ fold change > 1 and FDR-corrected *p* value < 0.05 (termed as differentially expressed proteins) in each of the indicated organelles.

Lysosomes

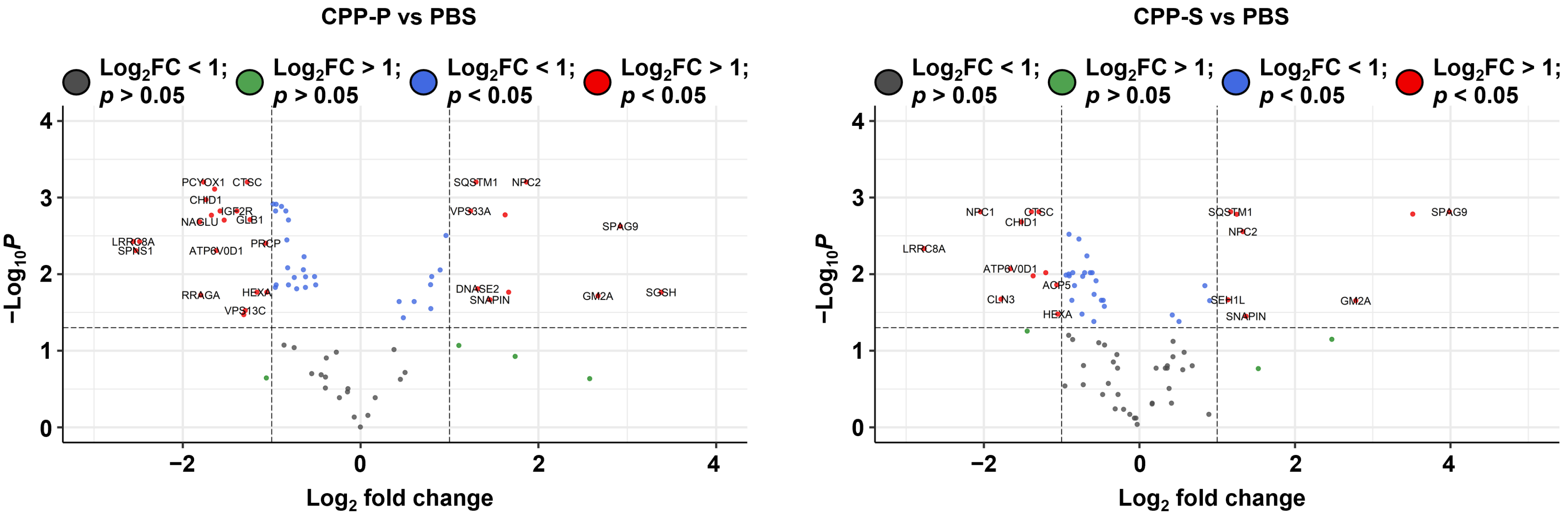

Supplementary Figure S8. Bioinformatic analysis of endoplasmic reticulum proteins in CPP-P- and CPP-S-treated human coronary artery endothelial cells (HCAEC) as compared to control (PBS-treated) cells, presented as volcano plots. Gray points depict the proteins with $\log_2$ fold change < 1 and FDR-corrected *p* value > 0.05, green points depict the proteins with $\log_2$ fold change > 1 and FDR-corrected *p* value > 0.05, blue points depict the proteins with $\log_2$ fold change < 1 and FDR-corrected *p* value < 0.05, and red points depict the proteins with $\log_2$ fold change > 1 and FDR-corrected *p* value < 0.05 (termed as differentially expressed proteins) in each of the indicated organelles.

Endoplasmic reticulum

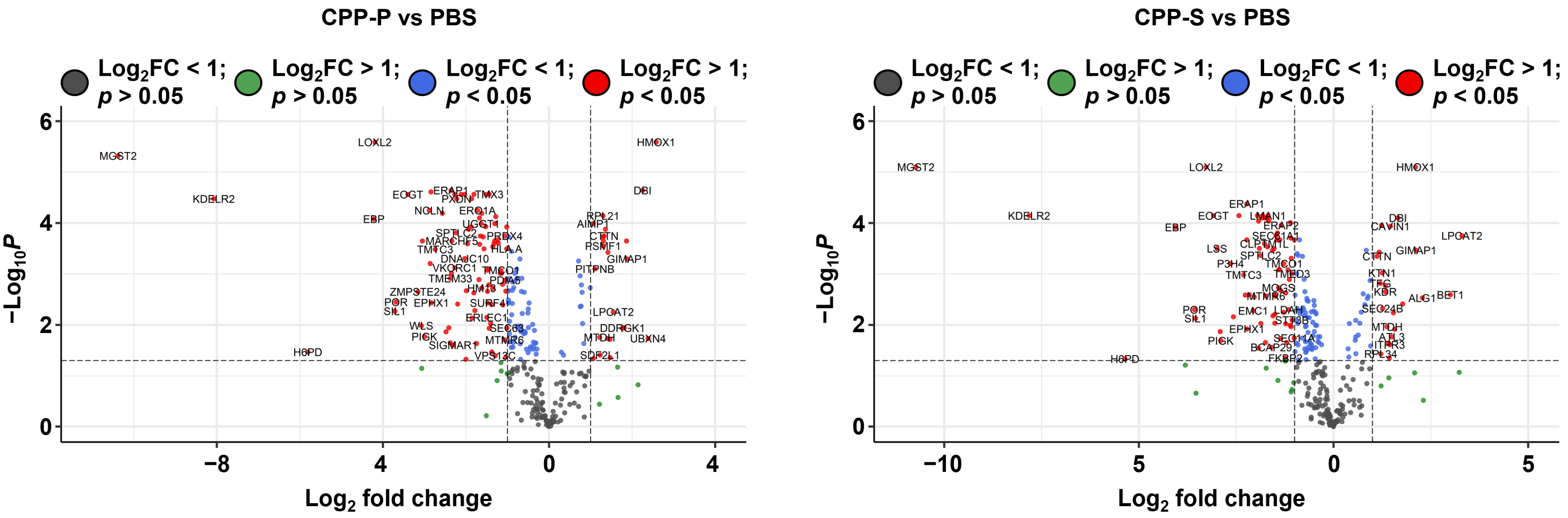

Supplementary Figure S9. Bioinformatic analysis of cytosolic proteins in CPP-P-treated human coronary artery endothelial cells (HCAEC) as compared to CPP-S-treated cells, presented as volcano plots. Gray points depict the proteins with $\log_2$ fold change < 1 and FDR-corrected *p* value > 0.05, green points depict the proteins with $\log_2$ fold change > 1 and FDR-corrected *p* value > 0.05, blue points depict the proteins with $\log_2$ fold change < 1 and FDR-corrected *p* value < 0.05, and red points depict the proteins with $\log_2$ fold change > 1 and FDR-corrected *p* value < 0.05 (termed as differentially expressed proteins) in each of the indicated organelles.

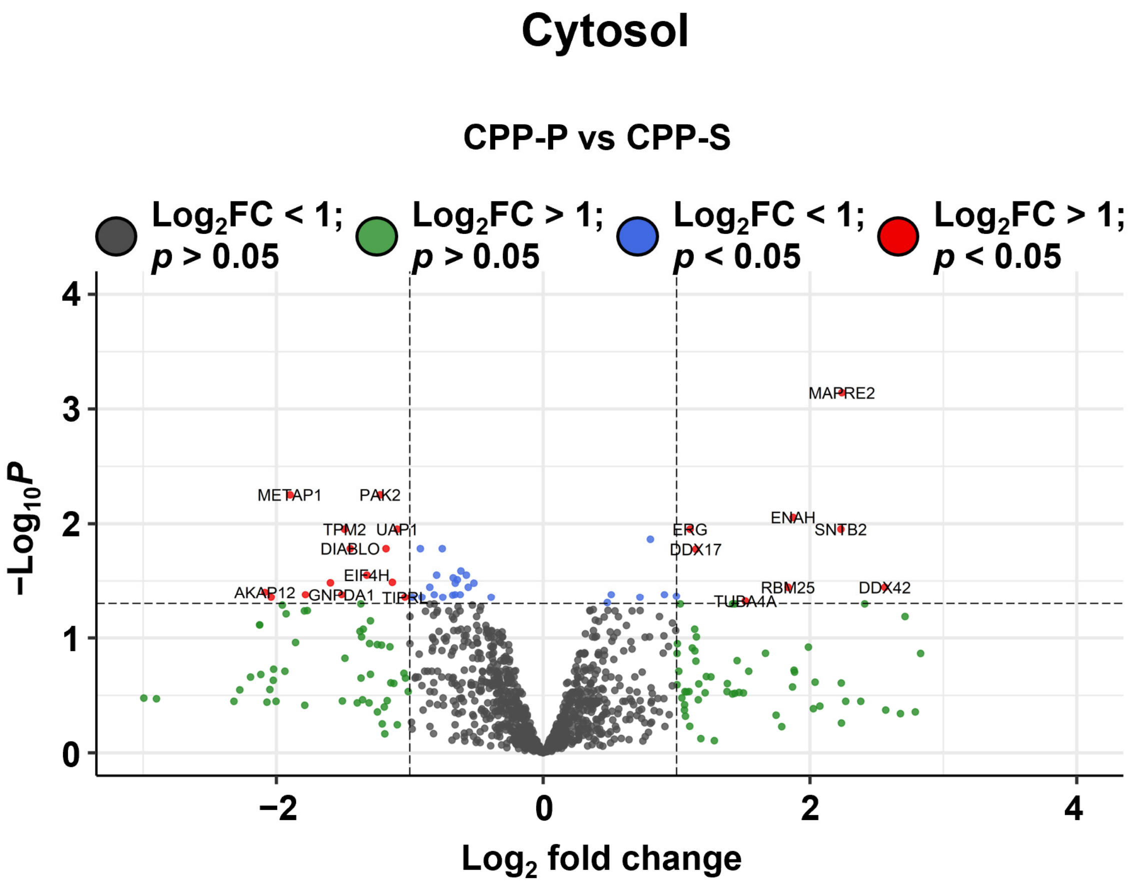

Supplementary Figure S10. Bioinformatic analysis of nuclear proteins in CPP-P-treated human coronary artery endothelial cells (HCAEC) as compared to CPP-S-treated cells, presented as volcano plots. Gray points depict the proteins with log_2 fold change < 1 and FDR-corrected *p* value > 0.05, green points depict the proteins with log_2 fold change > 1 and FDR-corrected *p* value > 0.05, blue points depict the proteins with log_2 fold change < 1 and FDR-corrected *p* value < 0.05, and red points depict the proteins with log_2 fold change > 1 and FDR-corrected *p* value < 0.05 (termed as differentially expressed proteins) in each of the indicated organelles.

Nuclei

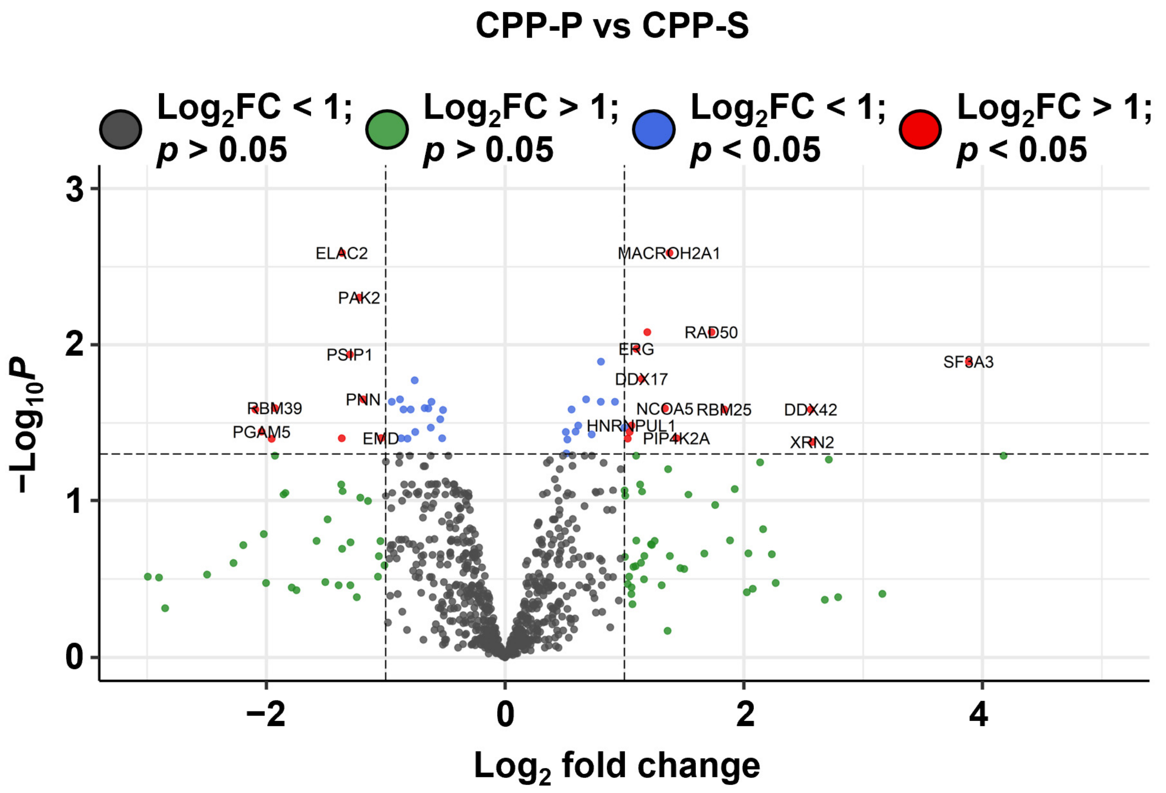

Supplementary Figure S11. Bioinformatic analysis of mitochondrial proteins in CPP-P-treated human coronary artery endothelial cells (HCAEC) as compared to CPP-S-treated cells, presented as volcano plots. Gray points depict the proteins with $\log_2$ fold change < 1 and FDR-corrected p value > 0.05, green points depict the proteins with $\log_2$ fold change > 1 and FDR-corrected p value > 0.05, blue points depict the proteins with $\log_2$ fold change < 1 and FDR-corrected p value < 0.05, and red points depict the proteins with $\log_2$ fold change > 1 and FDR-corrected p value < 0.05 (termed as differentially expressed proteins) in each of the indicated organelles.

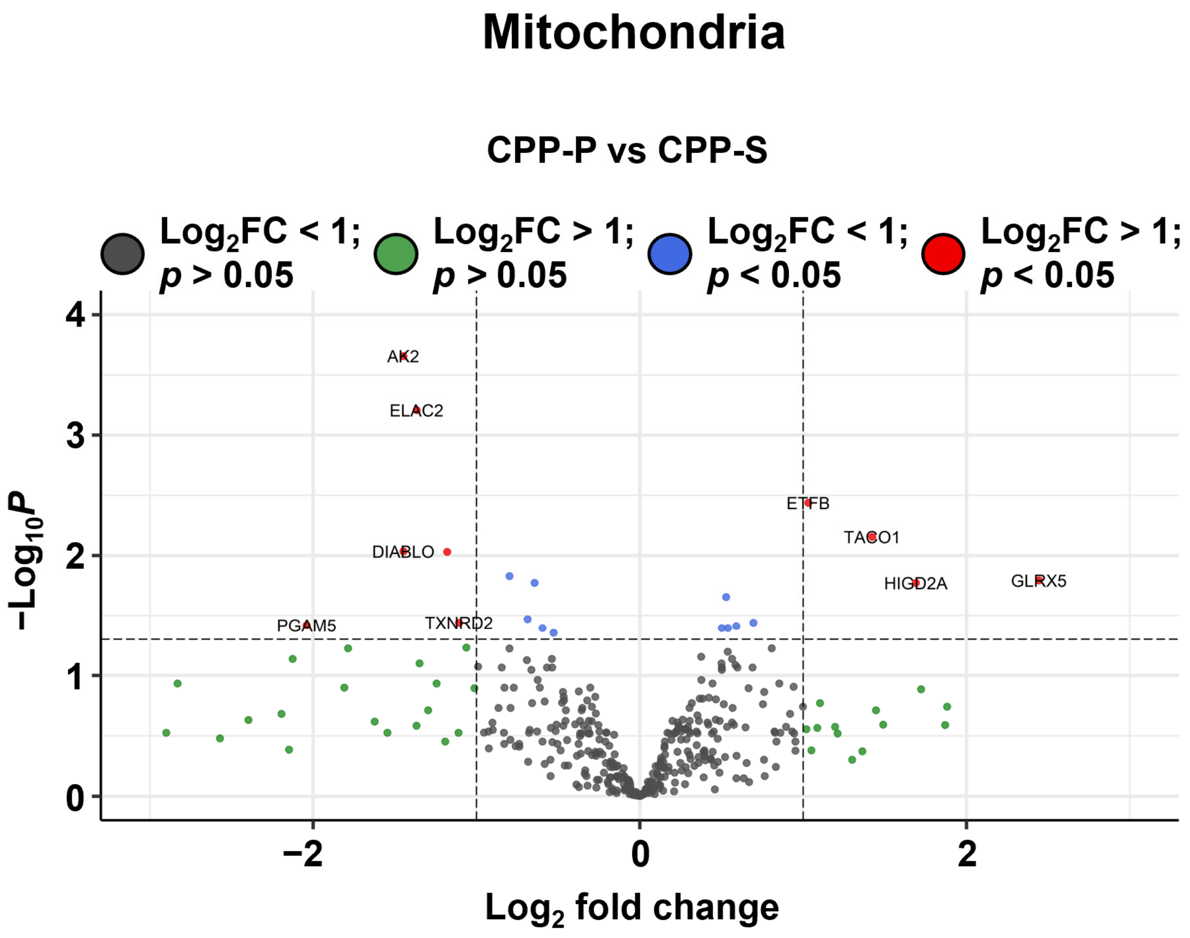

Supplementary Figure S12. Bioinformatic analysis of lysosomal proteins in CPP-P-treated human coronary artery endothelial cells (HCAEC) as compared to CPP-S-treated cells, presented as volcano plots. Gray points depict the proteins with $\log_2$ fold change < 1 and FDR-corrected p value > 0.05, green points depict the proteins with $\log_2$ fold change > 1 and FDR-corrected p value > 0.05, blue points depict the proteins with $\log_2$ fold change < 1 and FDR-corrected p value < 0.05, and red points depict the proteins with $\log_2$ fold change > 1 and FDR-corrected p value < 0.05 (termed as differentially expressed proteins) in each of the indicated organelles.

Lysosomes

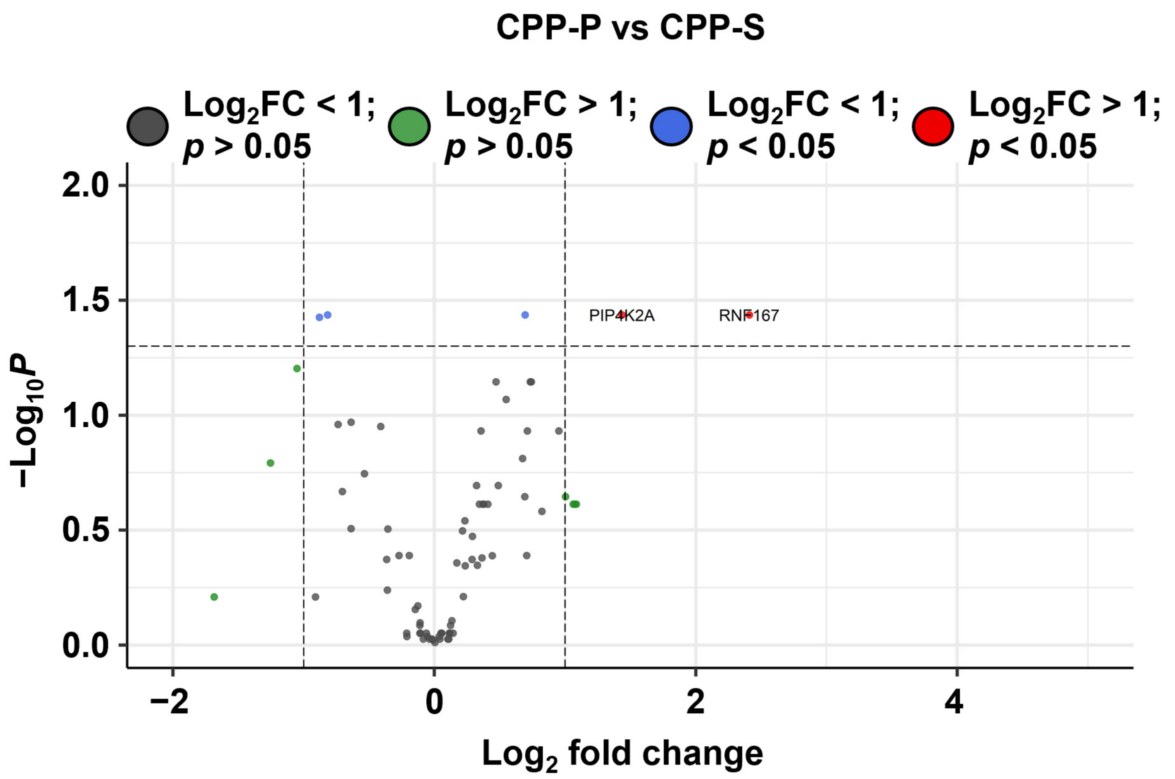

Supplementary Figure S13. Bioinformatic analysis of endoplasmic reticulum proteins in CPP-P-treated human coronary artery endothelial cells (HCAEC) as compared to CPP-S-treated cells, presented as volcano plots. Gray points depict the proteins with $\log_2$ fold change < 1 and FDR-corrected *p* value > 0.05, green points depict the proteins with $\log_2$ fold change > 1 and FDR-corrected *p* value > 0.05, blue points depict the proteins with $\log_2$ fold change < 1 and FDR-corrected *p* value < 0.05, and red points depict the proteins with $\log_2$ fold change > 1 and FDR-corrected *p* value < 0.05 (termed as differentially expressed proteins) in each of the indicated organelles.

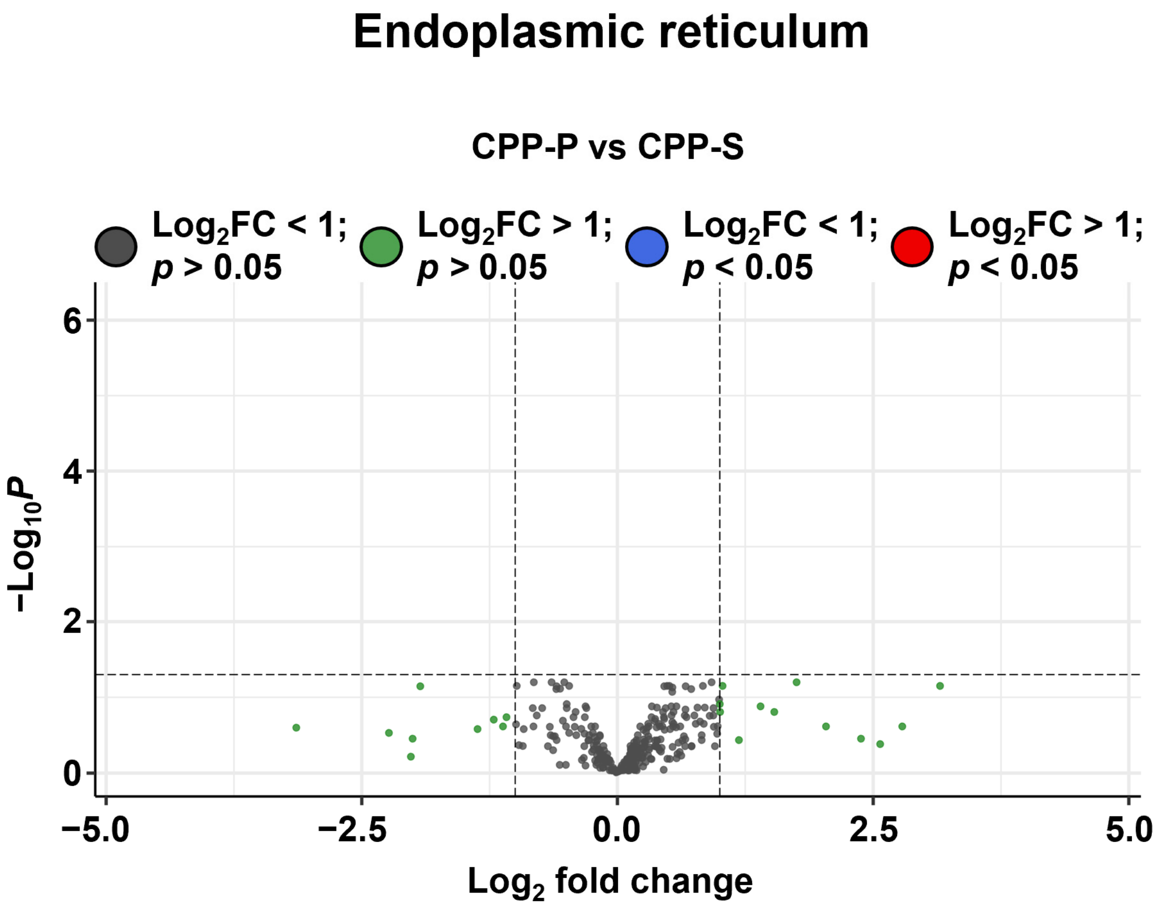

Supplementary Figure S14. Bioinformatic analysis of cytosolic proteins in CPP-P- and CPP-S-treated human internal thoracic artery endothelial cells (HITAEC) as compared to control (PBS-treated) cells, presented as volcano plots. Gray points depict the proteins with $\log_2$ fold change < 1 and FDR-corrected *p* value > 0.05, green points depict the proteins with $\log_2$ fold change > 1 and FDR-corrected *p* value > 0.05, blue points depict the proteins with $\log_2$ fold change < 1 and FDR-corrected *p* value < 0.05, and red points depict the proteins with $\log_2$ fold change > 1 and FDR-corrected *p* value < 0.05 (termed as differentially expressed proteins) in each of the indicated organelles.

Cytosol

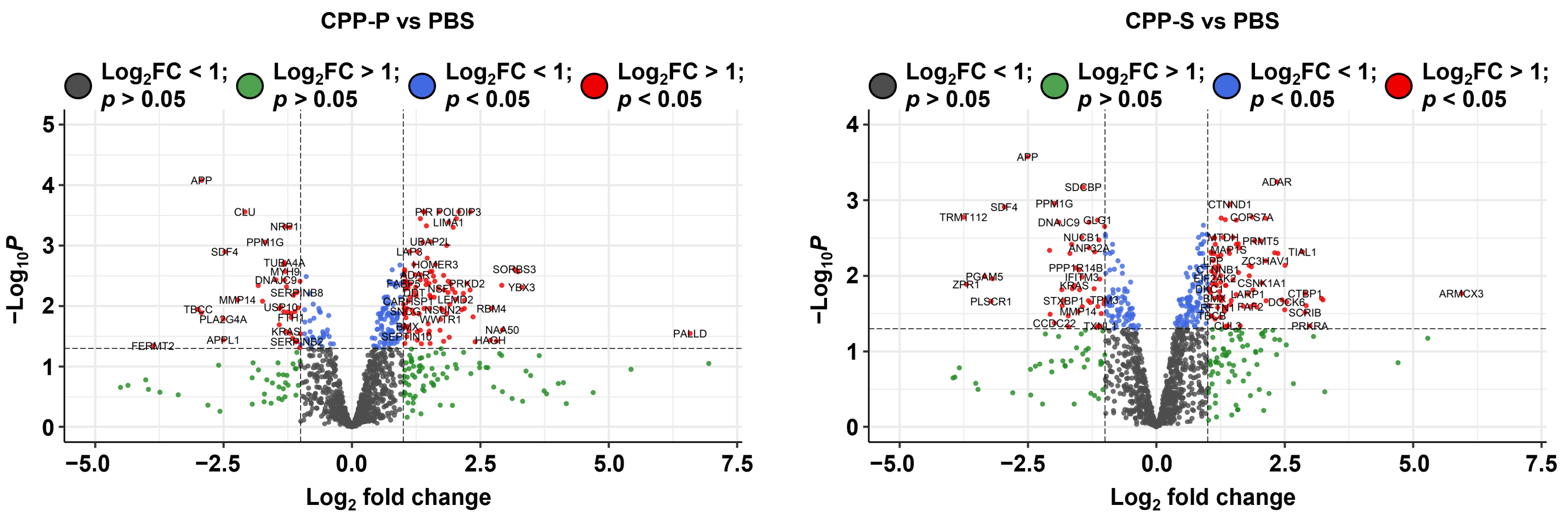

Supplementary Figure S15. Bioinformatic analysis of nuclear proteins in CPP-P- and CPP-S-treated human internal thoracic artery endothelial cells (HITAEC) as compared to control (PBS-treated) cells, presented as volcano plots. Gray points depict the proteins with $\log_2$ fold change < 1 and FDR-corrected *p* value > 0.05, green points depict the proteins with $\log_2$ fold change > 1 and FDR-corrected *p* value > 0.05, blue points depict the proteins with $\log_2$ fold change < 1 and FDR-corrected *p* value < 0.05, and red points depict the proteins with $\log_2$ fold change > 1 and FDR-corrected *p* value < 0.05 (termed as differentially expressed proteins) in each of the indicated organelles.

Nuclei

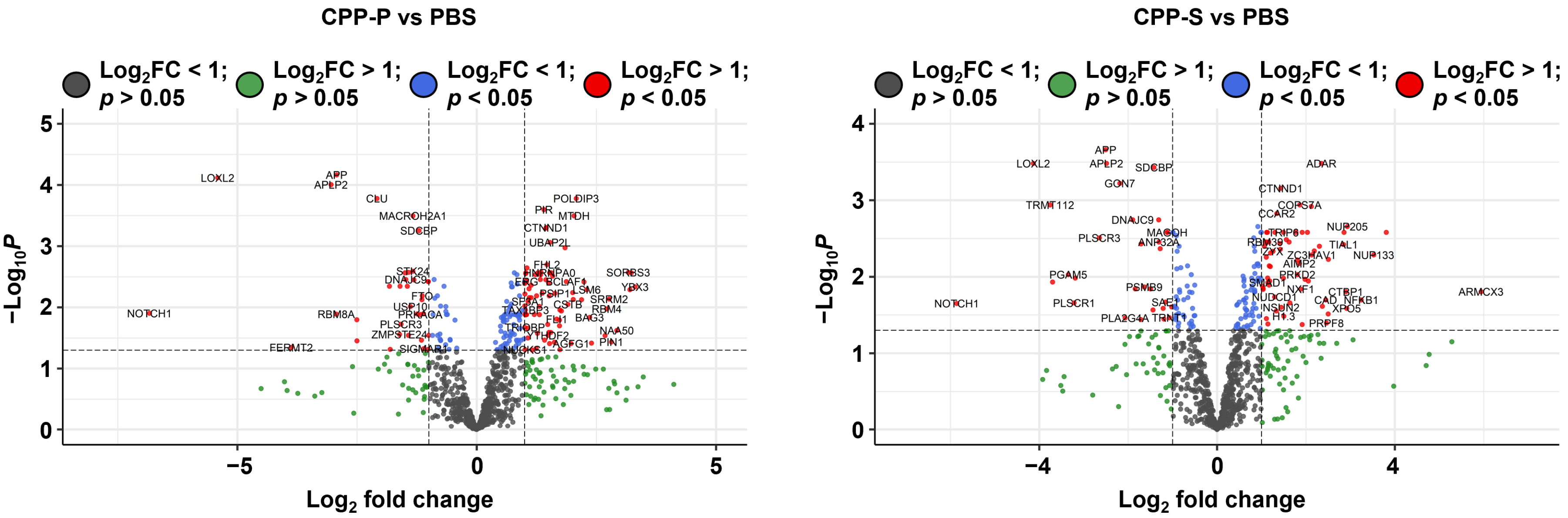

Supplementary Figure S16. Bioinformatic analysis of mitochondrial proteins in CPP-P- and CPP-S-treated human internal thoracic artery endothelial cells (HITAEC) as compared to control (PBS-treated) cells, presented as volcano plots. Gray points depict the proteins with $\log_2$ fold change < 1 and FDR-corrected *p* value > 0.05, green points depict the proteins with $\log_2$ fold change > 1 and FDR-corrected *p* value > 0.05, blue points depict the proteins with $\log_2$ fold change < 1 and FDR-corrected *p* value < 0.05, and red points depict the proteins with $\log_2$ fold change > 1 and FDR-corrected *p* value < 0.05 (termed as differentially expressed proteins) in each of the indicated organelles.

Mitochondria

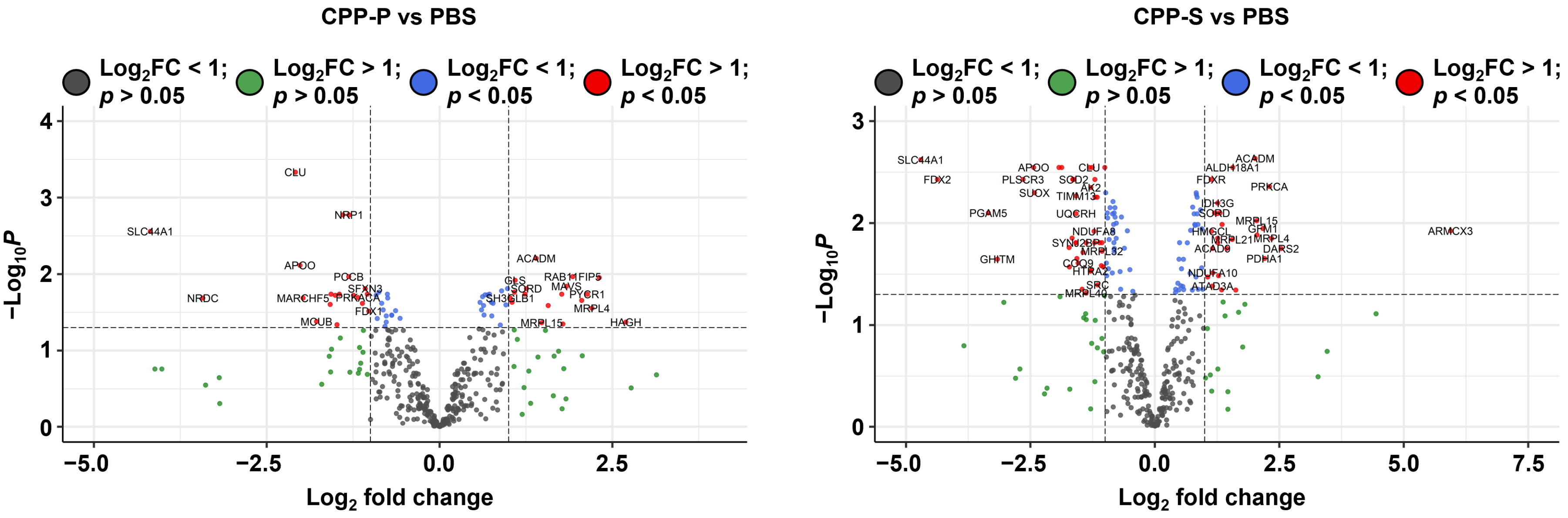

Supplementary Figure S17. Bioinformatic analysis of lysosomal proteins in CPP-P- and CPP-S-treated human internal thoracic artery endothelial cells (HITAEC) as compared to control (PBS-treated) cells, presented as volcano plots. Gray points depict the proteins with $\log_2$ fold change < 1 and FDR-corrected *p* value > 0.05, green points depict the proteins with $\log_2$ fold change > 1 and FDR-corrected *p* value > 0.05, blue points depict the proteins with $\log_2$ fold change < 1 and FDR-corrected *p* value < 0.05, and red points depict the proteins with $\log_2$ fold change > 1 and FDR-corrected *p* value < 0.05 (termed as differentially expressed proteins) in each of the indicated organelles.

Lysosomes

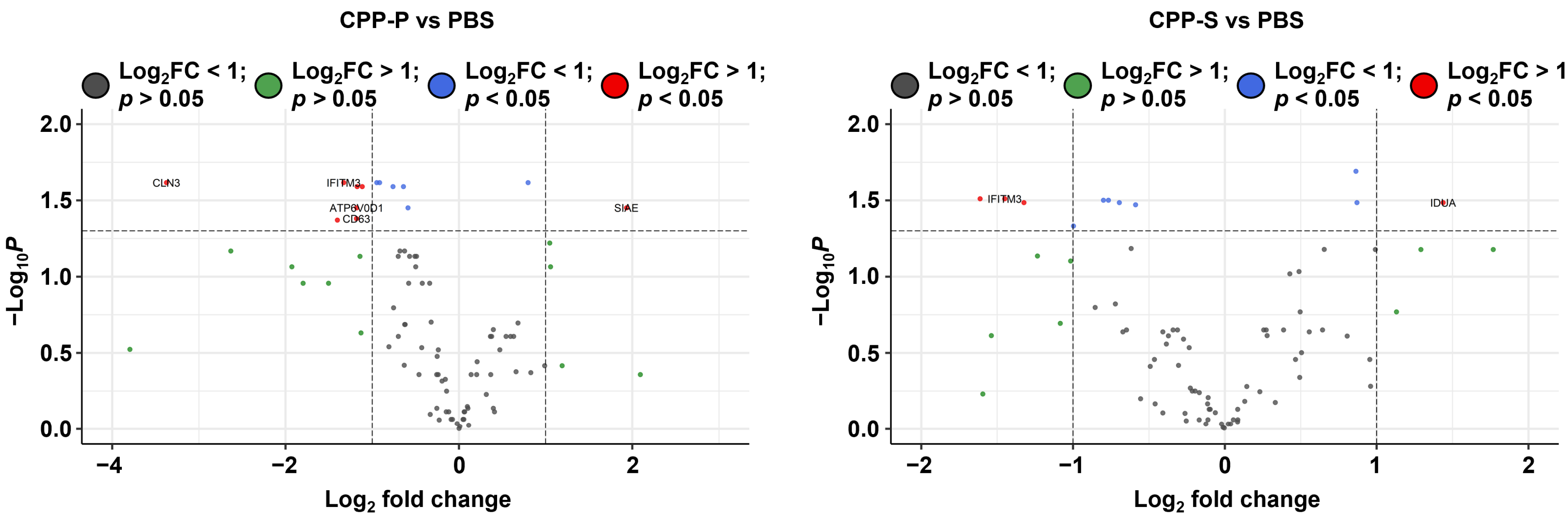

Supplementary Figure S18. Bioinformatic analysis of endoplasmic reticulum proteins in CPP-P- and CPP-S-treated human internal thoracic artery endothelial cells (HITAEC) as compared to control (PBS-treated) cells, presented as volcano plots. Gray points depict the proteins with $\log_2$ fold change < 1 and FDR-corrected *p* value > 0.05, green points depict the proteins with $\log_2$ fold change > 1 and FDR-corrected *p* value > 0.05, blue points depict the proteins with $\log_2$ fold change < 1 and FDR-corrected *p* value < 0.05, and red points depict the proteins with $\log_2$ fold change > 1 and FDR-corrected *p* value < 0.05 (termed as differentially expressed proteins) in each of the indicated organelles.

Endoplasmic reticulum

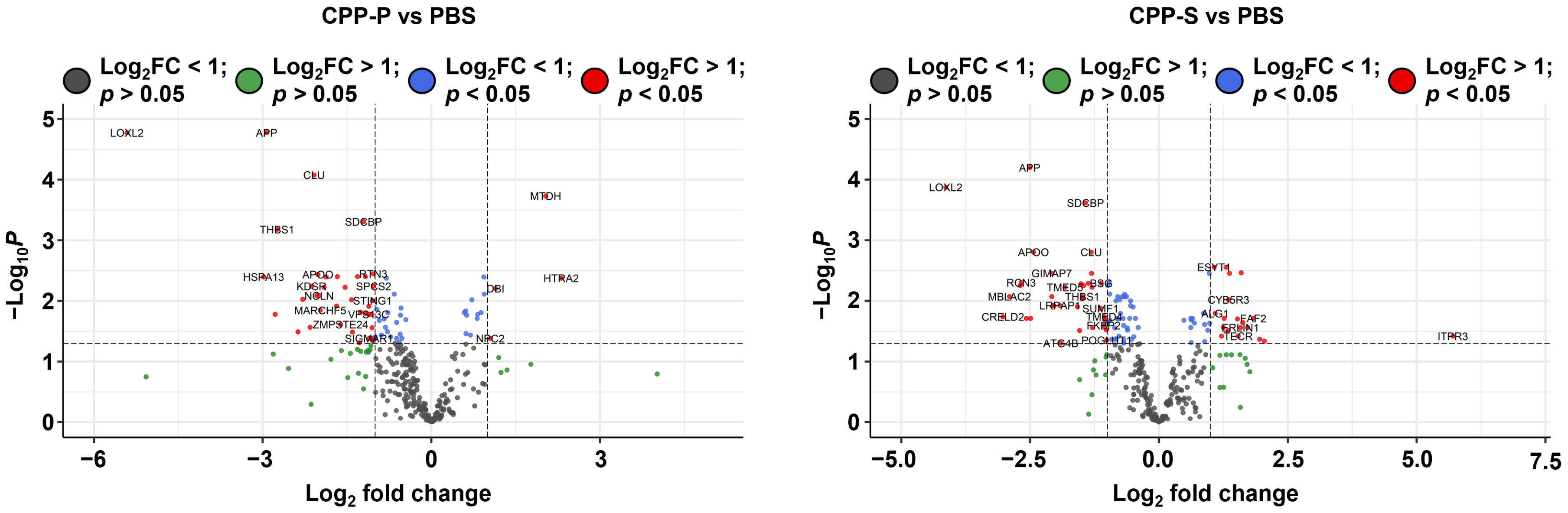

Supplementary Figure S19. Bioinformatic analysis of cytosolic proteins in CPP-P-treated human internal thoracic artery endothelial cells (HITAEC) as compared to CPP-S-treated cells, presented as volcano plots. Gray points depict the proteins with $\log_2$ fold change < 1 and FDR-corrected *p* value > 0.05, green points depict the proteins with $\log_2$ fold change > 1 and FDR-corrected *p* value > 0.05, blue points depict the proteins with $\log_2$ fold change < 1 and FDR-corrected *p* value < 0.05, and red points depict the proteins with $\log_2$ fold change > 1 and FDR-corrected *p* value < 0.05 (termed as differentially expressed proteins) in each of the indicated organelles.

Cytosol

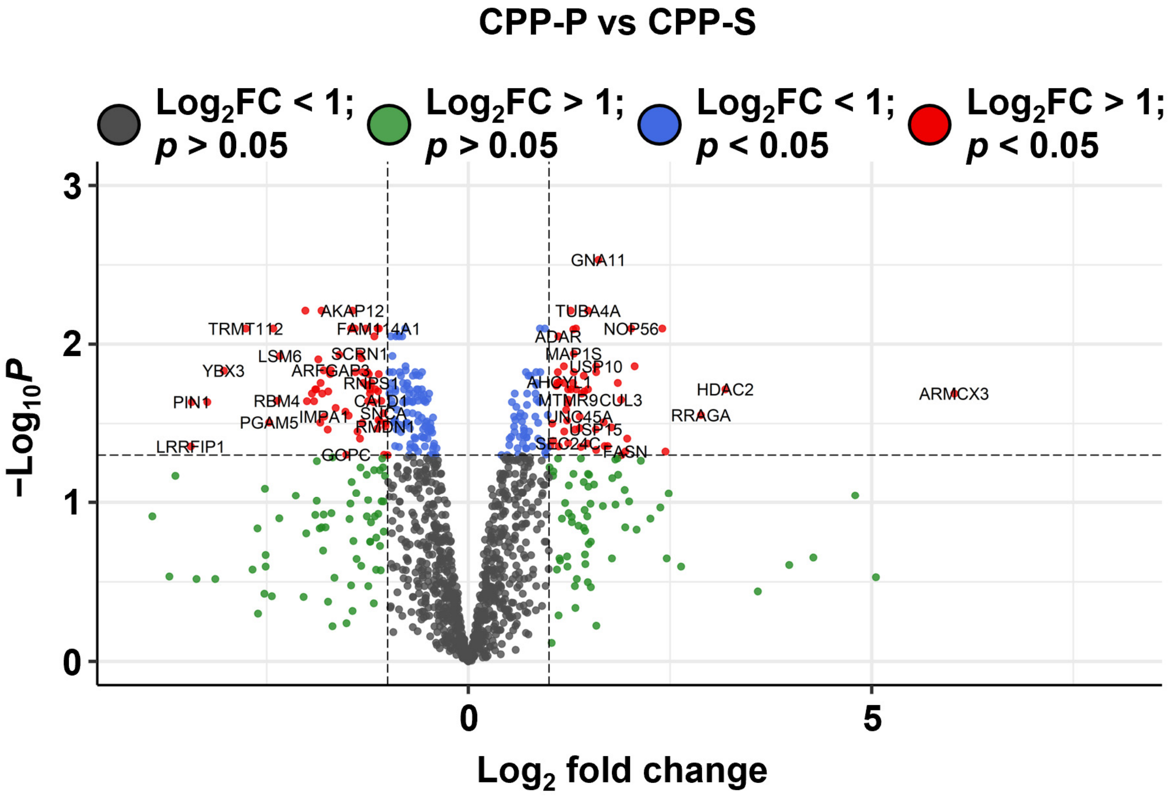

Supplementary Figure S20. Bioinformatic analysis of nuclear proteins in CPP-P-treated human internal thoracic artery endothelial cells (HITAEC) as compared to CPP-S-treated cells, presented as volcano plots. Gray points depict the proteins with $\log_2$ fold change < 1 and FDR-corrected p value > 0.05, green points depict the proteins with $\log_2$ fold change > 1 and FDR-corrected p value > 0.05, blue points depict the proteins with $\log_2$ fold change < 1 and FDR-corrected p value < 0.05, and red points depict the proteins with $\log_2$ fold change > 1 and FDR-corrected p value < 0.05 (termed as differentially expressed proteins) in each of the indicated organelles.

Nuclei

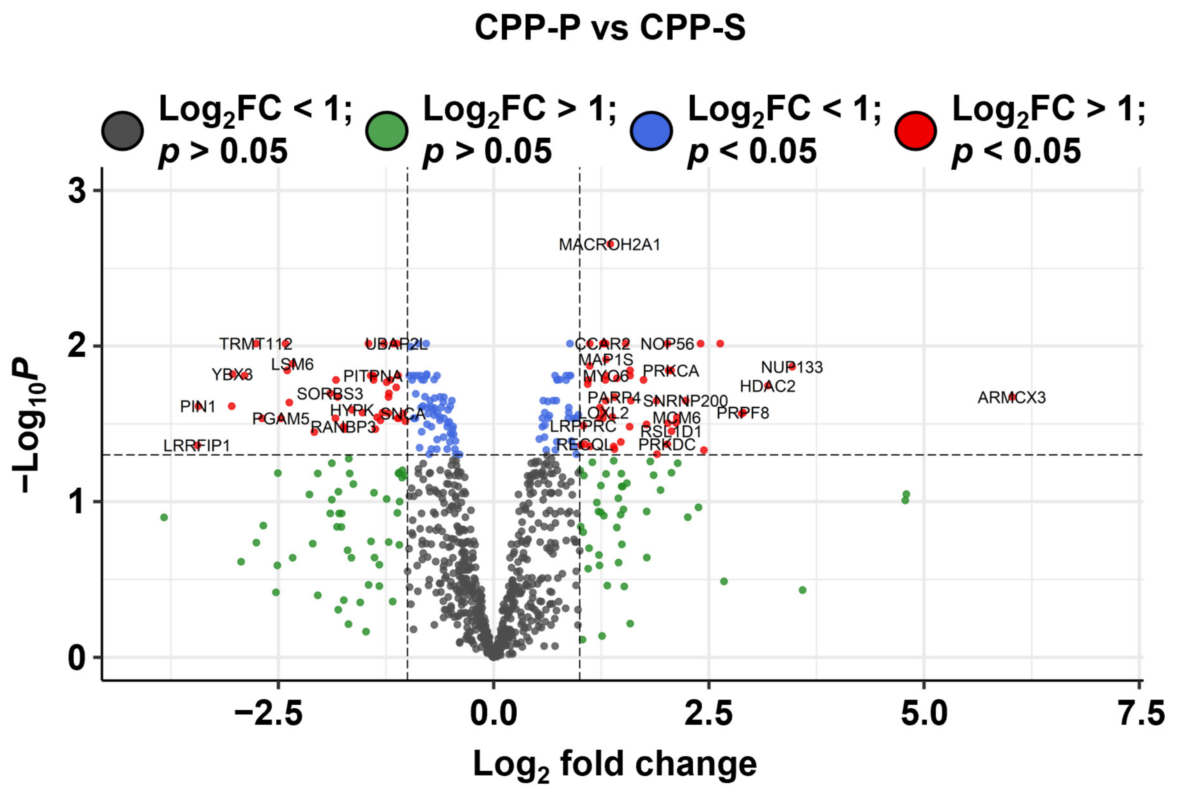

Supplementary Figure S21. Bioinformatic analysis of mitochondrial proteins in CPP-P-treated human internal thoracic artery endothelial cells (HITAEC) as compared to CPP-S-treated cells, presented as volcano plots. Gray points depict the proteins with $\log_2$ fold change < 1 and FDR-corrected *p* value > 0.05, green points depict the proteins with $\log_2$ fold change > 1 and FDR-corrected *p* value > 0.05, blue points depict the proteins with $\log_2$ fold change < 1 and FDR-corrected *p* value < 0.05, and red points depict the proteins with $\log_2$ fold change > 1 and FDR-corrected *p* value < 0.05 (termed as differentially expressed proteins) in each of the indicated organelles.

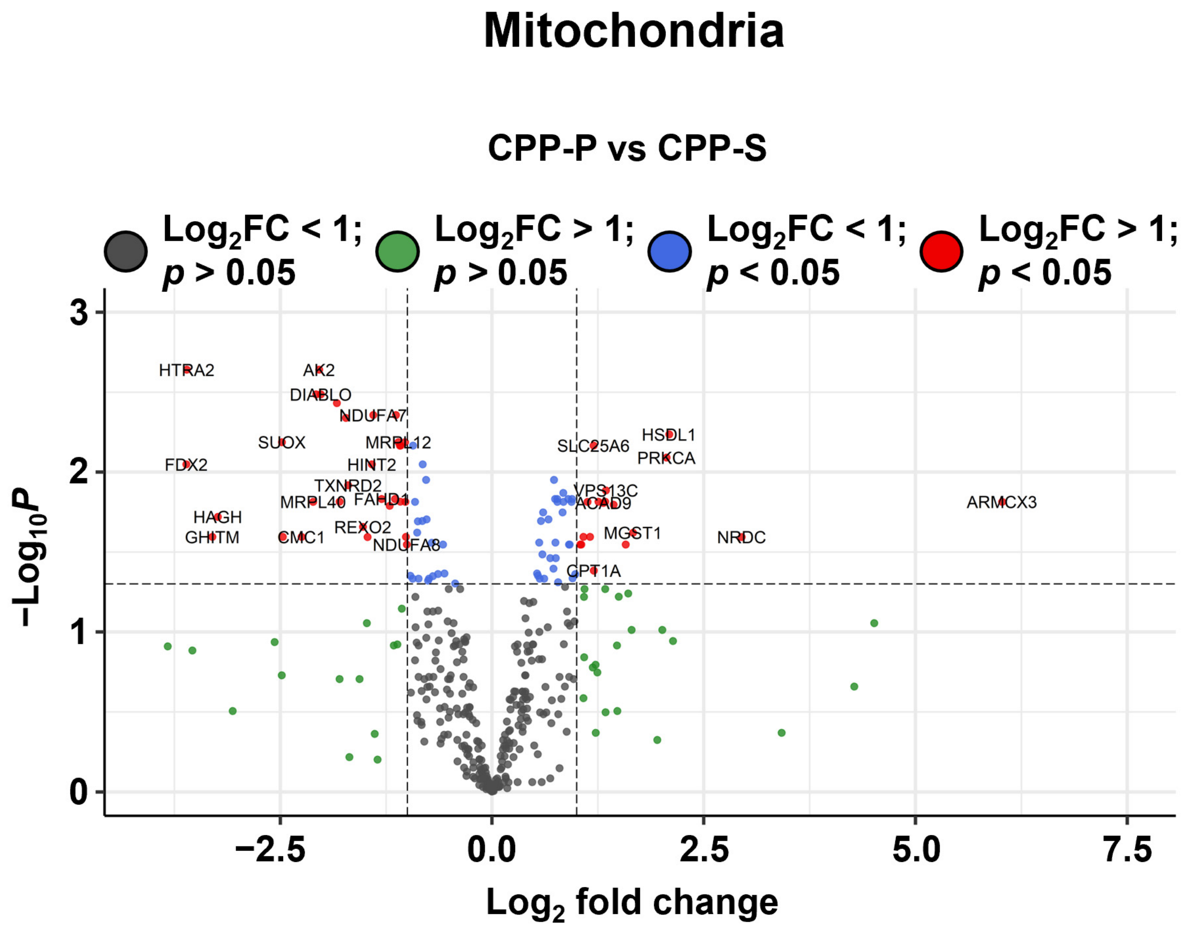

Supplementary Figure S22. Bioinformatic analysis of lysosomal proteins in CPP-P-treated human internal thoracic artery endothelial cells (HITAEC) as compared to CPP-S-treated cells, presented as volcano plots. Gray points depict the proteins with $\log_2$ fold change < 1 and FDR-corrected *p* value > 0.05, green points depict the proteins with $\log_2$ fold change > 1 and FDR-corrected *p* value > 0.05, blue points depict the proteins with $\log_2$ fold change < 1 and FDR-corrected *p* value < 0.05, and red points depict the proteins with $\log_2$ fold change > 1 and FDR-corrected *p* value < 0.05 (termed as differentially expressed proteins) in each of the indicated organelles.

Lysosomes

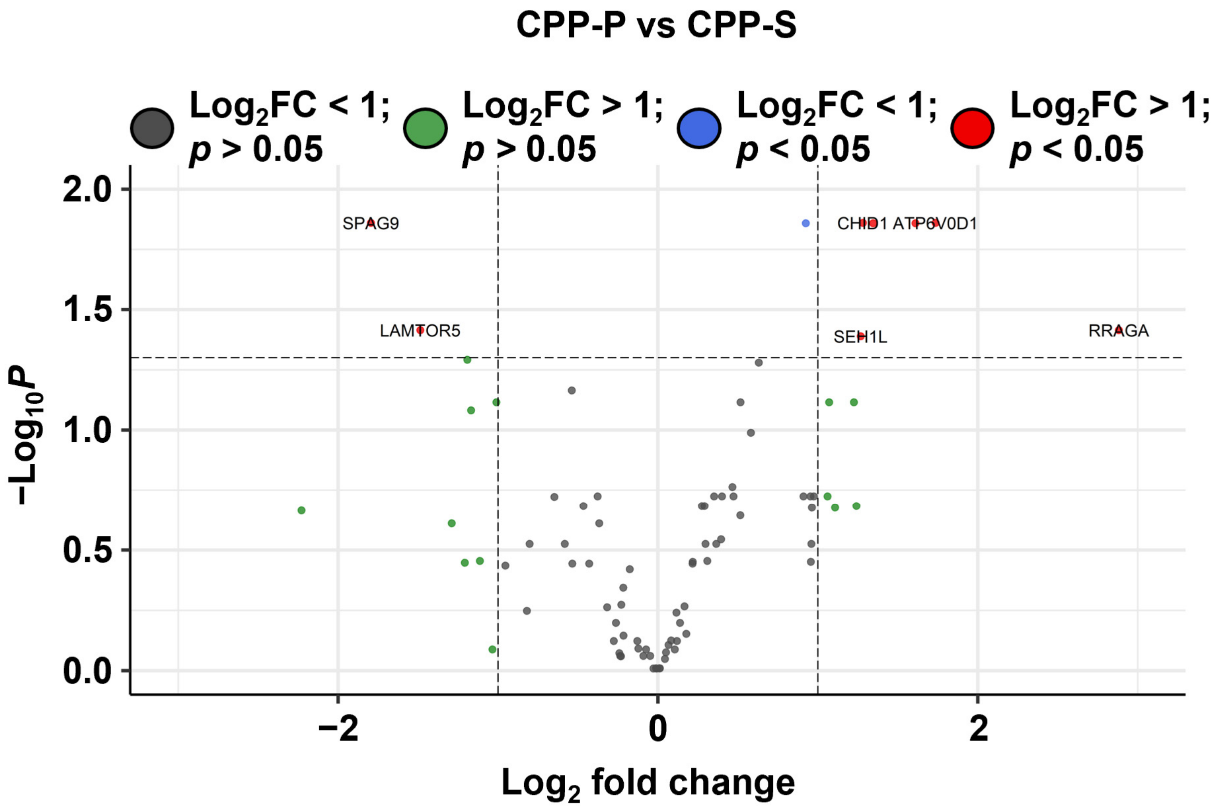

Supplementary Figure S23. Bioinformatic analysis of endoplasmic reticulum proteins in CPP-P-treated human internal thoracic artery endothelial cells (HITAEC) as compared to CPP-S-treated cells, presented as volcano plots. Gray points depict the proteins with $\log_2$ fold change < 1 and FDR-corrected *p* value > 0.05, green points depict the proteins with $\log_2$ fold change > 1 and FDR-corrected *p* value > 0.05, blue points depict the proteins with $\log_2$ fold change < 1 and FDR-corrected *p* value < 0.05, and red points depict the proteins with $\log_2$ fold change > 1 and FDR-corrected *p* value < 0.05 (termed as differentially expressed proteins) in each of the indicated organelles.

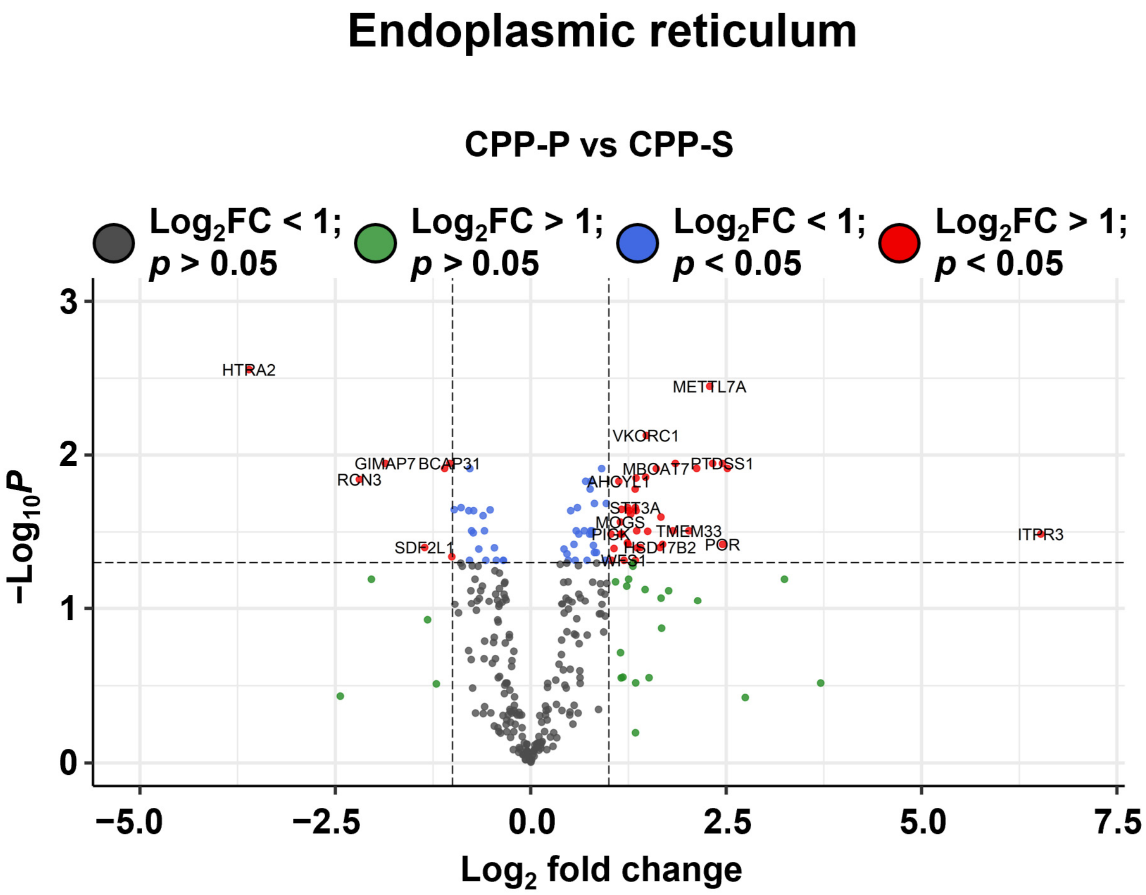

Supplementary Figure S24. Western blotting for nuclear and mitochondrial markers indicating successful fractionation of mitochondrial and nuclear fractions. **A.** Western blotting for nuclear loading control (proliferating cell nuclear antigen, PCNA). **B.** Western blotting for another nuclear loading control (TATA-binding protein, TBP). **C.** Reblot of the blot from B for mitochondrial loading control (voltage-dependent anion-selective channel 1 (VDAC1) / porin). Note the remaining bands for TATA-binding protein above the bands for VDAC1/porin after the reblot. MagicMark XP Western Protein Standard was used as a marker of molecular weight. L: ladder, M: mitochondria lysate, N: nuclear lysate, T – total (i.e., non-fractionated) protein used as a positive control. Shown are uncropped blots.

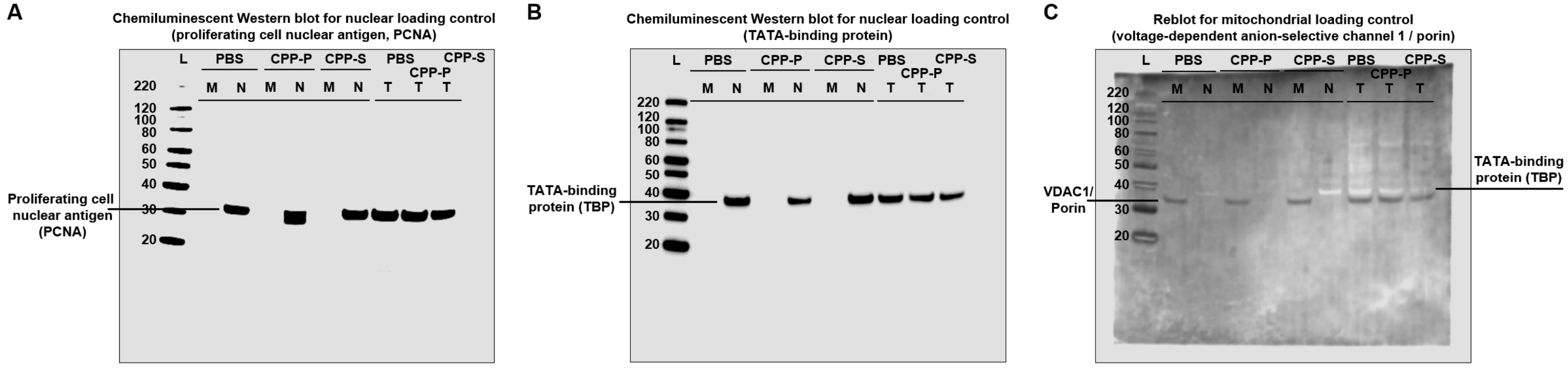

Supplementary Figure S25. Bioinformatic analysis of mitochondrial lysate in CPP-P- and CPP-S-treated human coronary artery endothelial cells (HCAEC) as compared to control (PBS-treated) cells, presented as volcano plots. Gray points depict the proteins with $\log_2$ fold change < 1 and FDR-corrected *p* value > 0.05, green points depict the proteins with $\log_2$ fold change > 1 and FDR-corrected *p* value > 0.05, blue points depict the proteins with $\log_2$ fold change < 1 and FDR-corrected *p* value < 0.05, and red points depict the proteins with $\log_2$ fold change > 1 and FDR-corrected *p* value < 0.05 (termed as differentially expressed proteins) in each of the indicated organelles.

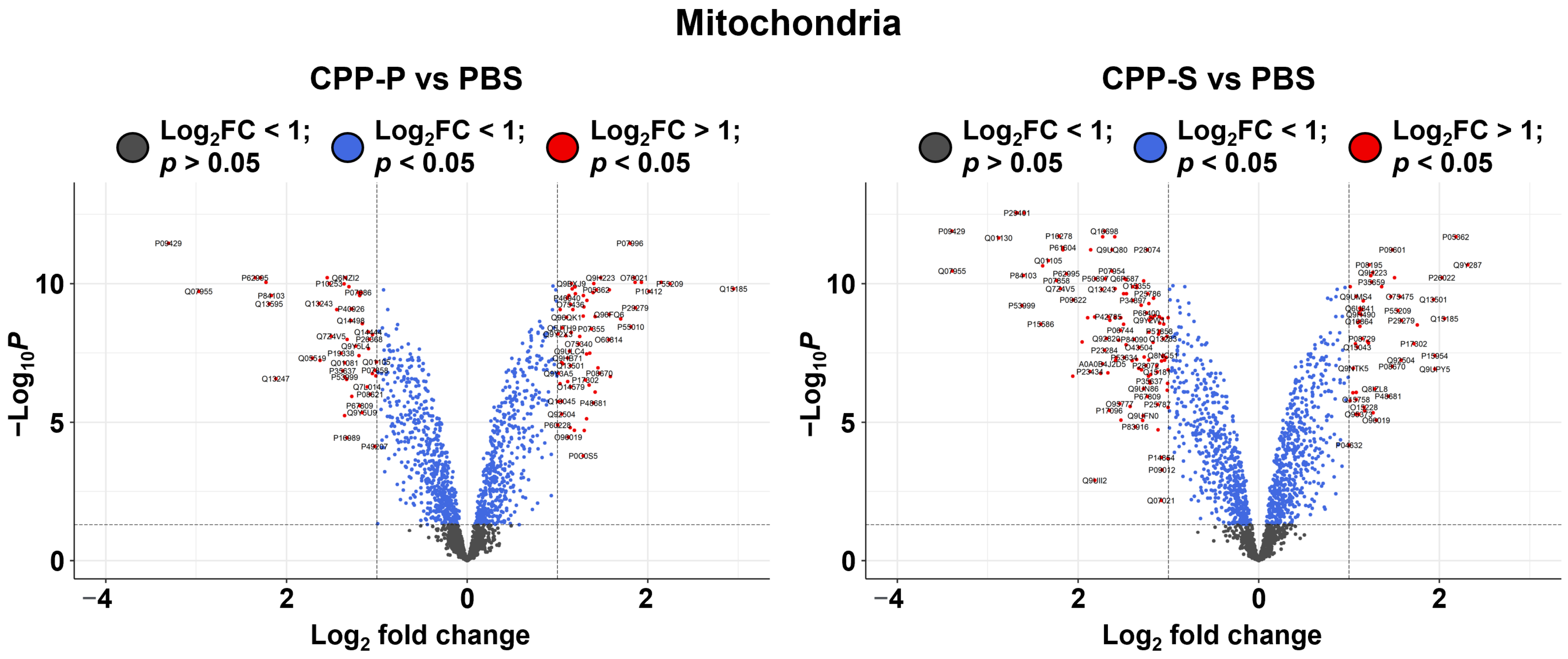

Supplementary Figure S26. Bioinformatic analysis of nuclear lysate in CPP-P- and CPP-S-treated human coronary artery endothelial cells (HCAEC) as compared to control (PBS-treated) cells, presented as volcano plots. Gray points depict the proteins with $\log_2$ fold change < 1 and FDR-corrected *p* value > 0.05, green points depict the proteins with $\log_2$ fold change > 1 and FDR-corrected *p* value > 0.05, blue points depict the proteins with $\log_2$ fold change < 1 and FDR-corrected *p* value < 0.05, and red points depict the proteins with $\log_2$ fold change > 1 and FDR-corrected *p* value < 0.05 (termed as differentially expressed proteins) in each of the indicated organelles.

Nuclei

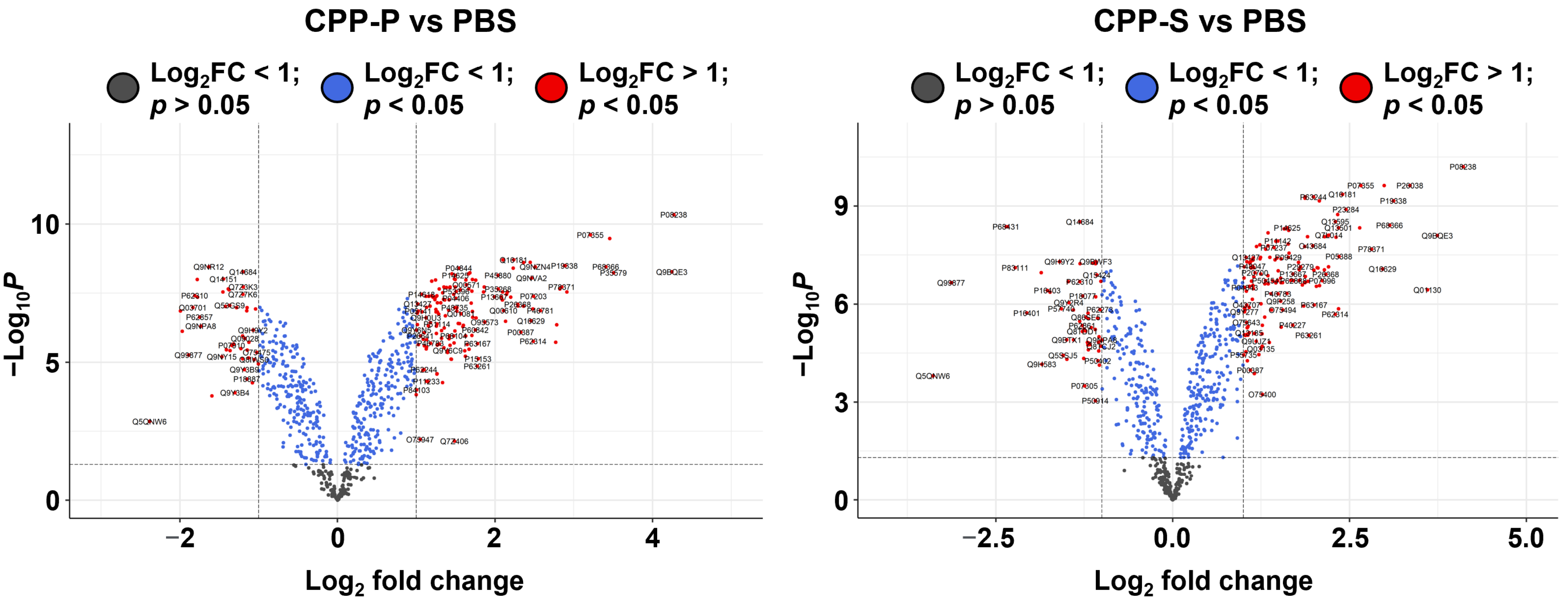

Supplementary Figure S27. Bioinformatic analysis of mitochondrial lysate in CPP-P-treated human coronary artery endothelial cells (HCAEC) as compared to CPP-S-treated cells, presented as volcano plots. Gray points depict the proteins with $\log_2$ fold change < 1 and FDR-corrected p value > 0.05, green points depict the proteins with $\log_2$ fold change > 1 and FDR-corrected p value > 0.05, blue points depict the proteins with $\log_2$ fold change < 1 and FDR-corrected p value < 0.05, and red points depict the proteins with $\log_2$ fold change > 1 and FDR-corrected p value < 0.05 (termed as differentially expressed proteins) in each of the indicated organelles.

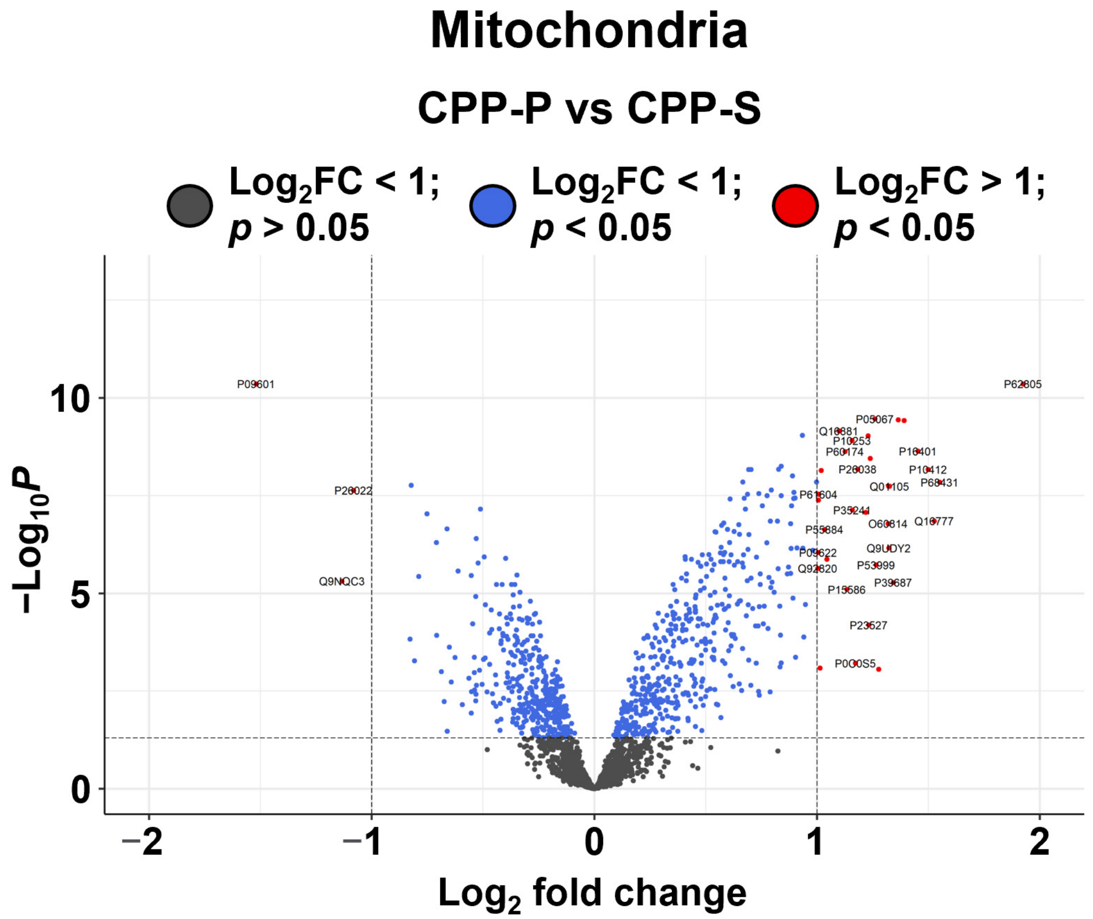

Supplementary Figure S28. Bioinformatic analysis of nuclear lysate in CPP-P-treated human coronary artery endothelial cells (HCAEC) as compared to CPP-S-treated cells, presented as volcano plots. Gray points depict the proteins with $\log_2$ fold change < 1 and FDR-corrected p value > 0.05, green points depict the proteins with $\log_2$ fold change > 1 and FDR-corrected p value > 0.05, blue points depict the proteins with $\log_2$ fold change < 1 and FDR-corrected p value < 0.05, and red points depict the proteins with $\log_2$ fold change > 1 and FDR-corrected p value < 0.05 (termed as differentially expressed proteins) in each of the indicated organelles.

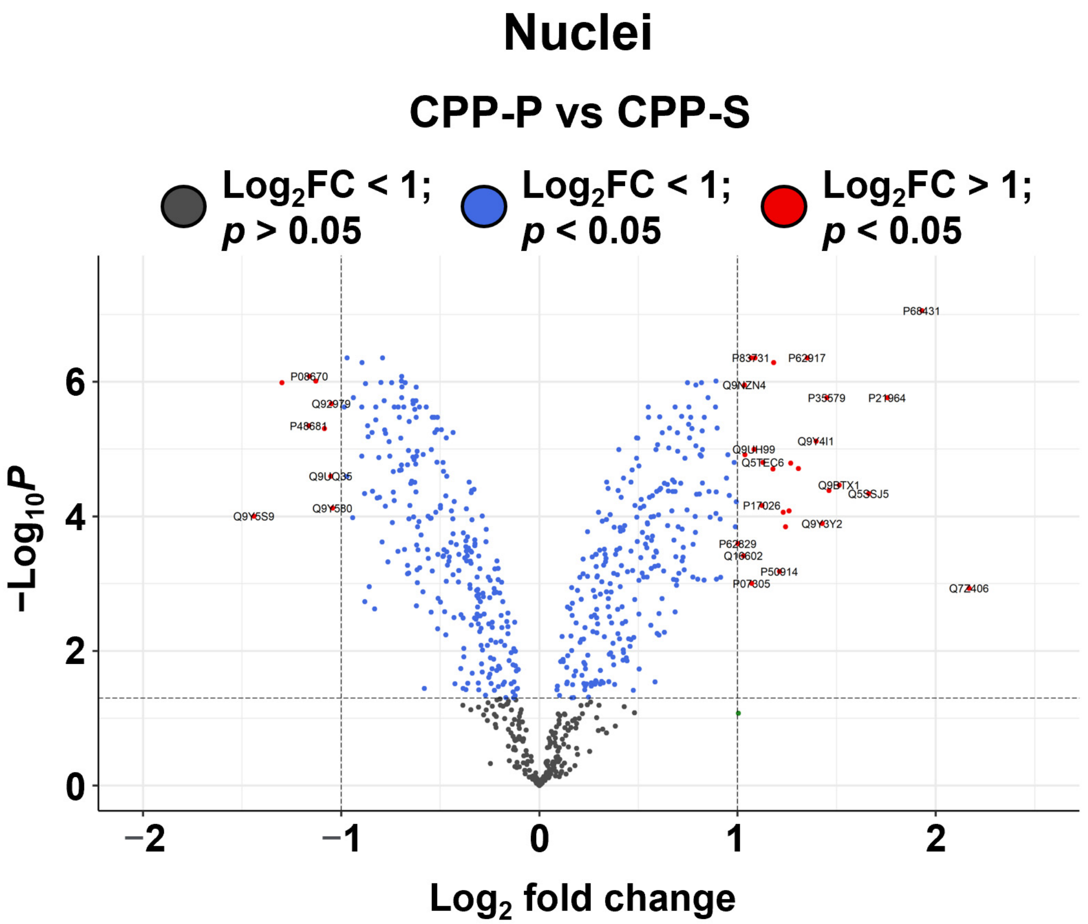

Supplement: Supplementary file 1 [file jcdd-11-00005-s001.zip › Supplementary Figures (S1-S28).pdf]
