# Supplementary material for: Calciprotein Particles Induce Cellular Compartment-Specific Proteome Alterations in Human Arterial Endothelial Cells
Source: J Cardiovasc Dev Dis. 2023 Dec 22;11(1):5. doi: 10.3390/jcdd11010005 (PMC10816121; doi:10.3390/jcdd11010005)
Supplement: Supplementary file 1 [file jcdd-11-00005-s001.zip › Supplementary Tables (S1-S19).pdf]

Supplementary Table S1. Sequences of primers used for RT-qPCR.

Gene	Forward primer	Reverse primer
IL1R1	GGCTGAAAAGCATAGAGGGAAC	CTGGGCTCACAATCACAGG
TNFRSF1A	CCAGGAGAAACAGAACACCGT	AAACCAATGAAGAGGAGGGATAA
TNFRSF1B	GTCCACACGATCCCAACAC	CACACCCACAATCAGTCCAA
IL1B	TGGCTTATTACAGTGGCAATG	GTGGTGGTCGGAGATTCG
IL6	GGCACTGGCAGAAAACAACC	GCAAGTCTCCTCATTGAATCC
CXCL8	CAGAGACAGCAGAGCACAC	AGTTCTTTAGCACTCCTTGGC
CCL2	TTCTGTGCCCTGCTGCTCATAG	AGGTGACTGGGGCATTGATTG
ACTA2	GTGTTGCCCCTGAAGAGCAT	GCTGGGACATTGAAAGTCTCA
SMTN	GGGATCGTGTCCACAAGTTCA	GCTACTCCTCGTTGCTCCTT
VIM	CGCCAGATGCGTGAAATGG	ACCAGAGGGAGTGAATCCAGA
COL1A1	GTCACCCACCGACCAAGAAACC	AAGTCCAGGCTGTCCAGGGATG
COL1A2	CCCCTGGTATGACTGGTTTCCC	GTCACCACGAGGACCACGAA
COL4A1	GGACTACCTGGAACAAAAGGG	GCCAAGTATCTCACCTGGATCA
MMP2	CCGTGTTTGCCATCTGTTTTAG	AGGTTCTCTTGCTGTTTACTTTGGA
RUNX2	AGATGGACCTCGGGAACCCA	TGAGGCGGGACACCTACTCT
SOX9	ACCAGTACCCGCACTTGCACA	CGCTTCTCGCTCTCGTTCAGAAGT

Supplementary Table S2. Fold enrichment (observed-versus-expected) analysis of differentially expressed proteins in cytosol of human coronary artery endothelial cells (HCAEC) treated with either primary CPPs (CPP-P) or control phosphate-buffer saline (PBS).

Molecular term	Total proteins	Percent from differentially expressed proteins	Fold enrichment	FDR-corrected P value
Upregulated after CPP-P treatment				
Hydrolase (UniProtKB Keywords)	17	17.5	1.9	3.8×10^{-1}
Innate immune system (Reactome)	15	15.5	2.0	1.4×10^{-1}
Cytokine signaling in immune system (Reactome)	14	14.4	2.8	4.8×10^{-2}
Ubiquitin-dependent protein catabolic process (GO BP)	9	9.3	6.4	4.4×10^{-2}
Apoptosis (UniProtKB Keywords)	9	9.3	2.6	1.2×10^{-1}
Ubiquitin protein ligase binding (GO MF)	8	8.2	5.1	3.6×10^{-2}
Signaling by interleukins (Reactome)	8	8.2	2.4	2.7×10^{-1}
Protein ubiquitination (GO BP)	7	7.2	3.1	8.7×10^{-1}
Programmed cell death (Reactome)	6	6.2	4.1	1.6×10^{-1}
Signaling by VEGF (Reactome)	5	5.2	6.7	9.5×10^{-2}
Chemokine signaling pathway (KEGG)	5	5.2	3.7	3.1×10^{-1}
Ca-dependent events (Reactome)	4	4.1	14.8	5.2×10^{-2}
VEGFA-VEGFR2 pathway (Reactome)	4	4.1	5.8	2.3×10^{-1}
Positive regulation of cell migration involved in sprouting angiogenesis (GO BP)	3	3.1	17.3	8.7×10^{-1}
Intrinsic apoptotic signaling pathway (GO BP)	3	3.1	16.9	8.7×10^{-1}
Downregulated after CPP-P treatment				
Metabolism of RNA (Reactome)	39	16.6	3.5	8.0×10^{-9}
Cadherin binding (GO MF)	35	14.9	9.0	3.9×10^{-20}
Cell cycle, mitotic (Reactome)	19	8.1	2.2	1.7×10^{-2}
mRNA processing (UniProtKB Keywords)	17	7.2	3.5	3.7×10^{-4}
Negative regulation of apoptotic process (GO BP)	17	7.2	2.8	3.2×10^{-2}
mRNA splicing (UniProtKB Keywords)	15	6.4	4.0	3.7×10^{-4}
mRNA transport (UniProtKB Keywords)	14	6.0	10.0	7.4×10^{-8}
Mitotic metaphase and anaphase (Reactome)	14	6.0	3.8	2.0×10^{-3}
Eukaryotic translation initiation (Reactome)	11	4.7	5.9	7.3×10^{-4}

Eukaryotic translation elongation (Reactome)	8	3.4	5.5	8.6×10^{-3}
Eukaryotic translation termination (Reactome)	8	3.4	5.5	8.6×10^{-3}
Ribosome (KEGG)	8	3.4	3.2	1.8×10^{-1}
Cell-cell adhesion (GO BP)	8	3.4	3.5	2.9×10^{-1}
Tight junction (KEGG)	8	3.4	3.2	1.8×10^{-1}
Cadherin binding involved in cell-cell adhesion (GO MF)	3	1.3	13.6	2.4×10^{-1}

Supplementary Table S3. Fold enrichment (observed-versus-expected) analysis of differentially expressed proteins in cytosol of human coronary artery endothelial cells (HCAEC) treated with either secondary CPPs (CPP-S) or control phosphate-buffer saline (PBS).

Molecular term	Total proteins	Percent from differentially expressed proteins	Fold enrichment	FDR-corrected P value
Upregulated after CPP-S treatment				
Hydrolase (UniProtKB Keywords)	20	18.7	1.9	4.3×10^{-2}
Cytokine signaling in immune system (Reactome)	16	15.0	3.0	2.9×10^{-2}
Signaling by interleukins (Reactome)	10	9.3	2.9	1.5×10^{-1}
Ubiquitin-dependent protein catabolic process (GO BP)	7	6.5	4.4	7.7×10^{-1}
Ubiquitin protein ligase binding (GO MF)	6	5.6	3.4	7.6×10^{-1}
Chemokine signaling pathway (KEGG)	6	5.6	3.6	1.3×10^{-1}
Signaling by VEGF (Reactome)	5	4.7	6.3	1.5×10^{-1}
Toll like receptor 4 (TLR4) cascade (Reactome)	5	4.7	4.6	2.4×10^{-1}
Apoptosis (KEGG)	5	4.7	4.2	1.4×10^{-1}
Ca-dependent events (Reactome)	4	3.7	13.9	1.0×10^{-1}
MyD88 cascade initiated on plasma membrane (Reactome)	4	3.7	5.6	2.5×10^{-1}
VEGFA-VEGFR2 pathway (Reactome)	4	3.7	5.5	2.6×10^{-1}
MyD88 dependent cascade initiated on endosome (Reactome)	4	3.7	5.2	2.7×10^{-1}
Intrinsic apoptotic signaling pathway (GO BP)	3	2.8	15.1	9.5×10^{-1}
Cellular response to reactive oxygen species (GO BP)	3	2.8	10.9	9.7×10^{-1}
Downregulated after CPP-S treatment				
Metabolism of RNA (Reactome)	45	20.7	4.4	9.4×10^{-15}
Cadherin binding (GO MF)	30	13.8	8.4	3.1×10^{-16}
mRNA processing (UniProtKB Keywords)	24	11.1	5.3	3.1×10^{-9}
Negative regulation of apoptotic process (GO BP)	17	7.8	3.0	1.6×10^{-2}
Cell cycle, mitotic (Reactome)	17	7.8	2.1	2.1×10^{-2}
mRNA transport (UniProtKB Keywords)	13	6.0	10.0	1.6×10^{-7}
RNA splicing (GO BP)	13	6.0	5.8	5.4×10^{-4}
rRNA processing (Reactome)	12	5.5	4.1	1.8×10^{-3}
Mitotic metaphase and anaphase (Reactome)	12	5.5	3.6	4.9×10^{-3}
Eukaryotic translation initiation (Reactome)	10	4.6	5.9	8.3×10^{-4}
Eukaryotic translation termination (Reactome)	9	4.1	6.7	8.3×10^{-4}
Eukaryotic translation elongation (Reactome)	9	4.1	6.7	8.3×10^{-4}
Ribosome (KEGG)	9	4.1	4.0	4.2×10^{-2}
Mitotic prophase (Reactome)	7	3.2	3.5	4.3×10^{-2}
Cell-cell adhesion (GO BP)	7	3.2	3.3	5.6×10^{-1}

Supplementary Table S4. Fold enrichment (observed-versus-expected) analysis of differentially expressed proteins in cytosol of human internal thoracic artery endothelial cells (HITAEC) treated with either primary CPPs (CPP-P) or control phosphate-buffer saline (PBS).

Molecular term	Total proteins	Percent from differentially expressed proteins	Fold enrichment	FDR-corrected P value
Upregulated after CPP-P treatment				
Immune system (Reactome)	15	38.5	2.4	2.1×10^{-1}
Innate immune system (Reactome)	10	25.6	3.0	3.0×10^{-1}
Cytokine signaling in immune system (Reactome)	7	17.9	3.1	1.0×10^{-1}
Signaling by VEGF (Reactome)	4	10.3	11.9	3.0×10^{-1}
Angiogenesis (GO BP)	4	10.3	7.8	8.2×10^{-1}
Positive regulation of cell migration (GO BP)	4	10.3	7.6	8.2×10^{-1}
VEGF signaling pathway (KEGG)	3	7.7	21.5	3.9×10^{-1}
Regulation of cell shape (GO BP)	3	7.7	10.0	1.0×10^{-1}
VEGFA-VEGFR2 pathway (Reactome)	3	7.7	9.8	1.0×10^{-1}
Positive regulation of NF-κB transcription factor activity (GO BP)	3	7.7	9.1	1.0×10^{-1}
Vascular endothelial growth factor binding (GO MF)	2	5.1	194.3	2.0×10^{-1}
Neurophilin interactions with VEGF and VEGFR (Reactome)	2	5.1	161.1	7.7×10^{-1}
Vascular endothelial growth factor-activated receptor activity (GO MF)	2	5.1	121.4	2.5×10^{-1}
Substrate-dependent cell migration, cell extension (GO BP)	2	5.1	110.6	8.4×10^{-1}
Cell migration involved in sprouting angiogenesis (GO BP)	2	5.1	47.4	1.0×10^{-1}
Calcium-mediated signaling using intracellular calcium source (GO BP)	2	5.1	43.3	1.0×10^{-1}
Downregulated after CPP-P treatment				
Cadherin binding (GO MF)	18	16.5	10.1	2.1×10^{-10}
Metabolism of RNA (Reactome)	12	11.0	2.6	1.0×10^{-1}
mRNA binding (GO MF)	11	10.1	7.8	7.9×10^{-5}
Actin-binding (UniProtKB Keywords)	10	9.2	5.5	1.3×10^{-3}
mRNA 3'-UTR binding (GO MF)	9	8.3	6.5	3.2×10^{-3}
Negative regulation of apoptotic process (GO BP)	9	8.3	3.2	8.5×10^{-1}
Cell adhesion (UniProtKB Keywords)	9	8.3	3.0	1.7×10^{-1}
mRNA transport (UniProtKB Keywords)	6	5.5	8.6	3.4×10^{-2}
Transcription cofactor activity (GO MF)	6	5.5	7.6	4.6×10^{-2}
Cell-cell adhesion (GO BP)	6	5.5	5.6	6.6×10^{-1}
Transcription, DNA-templated (GO BP)	6	5.5	5.6	6.6×10^{-1}
Negative regulation of translation (GO BP)	5	4.6	10.3	5.0×10^{-1}
Actin filament organization (GO BP)	5	4.6	5.9	8.5×10^{-1}
RNA splicing (GO BP)	5	4.6	4.4	1.0×10^{-1}
Regulation of mRNA stability (GO BP)	4	3.7	11.8	6.6×10^{-1}

Supplementary Table S5. Fold enrichment (observed-versus-expected) analysis of differentially expressed proteins in cytosol of human internal thoracic artery endothelial cells (HITAEC) treated with either secondary CPPs (CPP-S) or control phosphate-buffer saline (PBS).

Molecular term	Total proteins	Percent from differentially expressed proteins	Fold enrichment	FDR-corrected P value
Upregulated after CPP-S treatment				
Hydrolase (GO MF)	10	21.3	2.5	2.6×10^{-1}
Cytokine signaling in immune system (Reactome)	8	17.0	3.3	1.0×10^{-1}

Necroptosis (KEGG)	4	8.5	10.6	3.4×10^{-1}
Programmed cell death (Reactome)	4	8.5	5.7	1.0×10^{-1}
Positive regulation of apoptotic process (GO BP)	4	8.5	5.3	9.1×10^{-1}
VEGF signaling pathway (KEGG)	3	6.4	21.5	3.4×10^{-1}
Macroautophagy (GO BP)	3	6.4	18.8	9.1×10^{-1}
Signaling by VEGF (Reactome)	3	6.4	8.5	1.0×10^{-1}
Regulation of early endosome to late endosome transport (GO BP)	2	4.3	95.9	9.1×10^{-1}
Substrate-dependent cell migration, cell extension (GO BP)	2	4.3	95.9	9.1×10^{-1}
Downregulated after CPP-S treatment				
RNA binding (GO MF)	32	34.4	4.4	2.0×10^{-10}
Metabolism of proteins (Reactome)	21	22.6	1.5	8.4×10^{-1}
Cadherin binding (GO MF)	14	15.1	9.0	4.5×10^{-7}
Metabolism of RNA (Reactome)	14	15.1	2.8	1.5×10^{-1}
Cell cycle (UniProtKB Keywords)	10	10.8	2.2	2.8×10^{-1}
Cell adhesion (UniProtKB Keywords)	9	9.7	2.7	2.5×10^{-1}
Positive regulation of transcription, DNA-templated (GO BP)	9	9.7	2.6	1.0×10^{-1}
Apoptosis (UniProtKB Keywords)	9	9.7	2.5	2.5×10^{-1}
Negative regulation of apoptotic process (GO BP)	8	8.6	3.2	1.0×10^{-1}
Mitosis (UniProtKB Keywords)	6	6.5	3.1	2.8×10^{-1}
Protein stabilization (GO BP)	5	5.4	4.6	1.0×10^{-1}
Transcription coactivator activity (GO MF)	5	5.4	3.8	5.7×10^{-1}
ER-Golgi transport (UniProtKB Keywords)	4	4.3	6.3	2.5×10^{-1}
Mitotic cell cycle (GO BP)	4	4.3	5.5	1.0×10^{-1}
Stem cell division (GO BP)	2	2.2	59.6	1.0×10^{-1}

Supplementary Table S6. Fold enrichment (observed-versus-expected) analysis of differentially expressed proteins in cytosol of human internal thoracic artery endothelial cells (HITAEC) treated with either primary CPPs (CPP-P) or secondary CPPs (CPP-S).

Molecular term	Total proteins	Percent from differentially expressed proteins	Fold enrichment	FDR-corrected P value
Upregulated after CPP-P treatment				
Hydrolase (UniProtKB Keywords)	14	24.6	2.2	1.2×10^{-1}
Vesicle-mediated transport (Reactome)	10	17.5	3.5	6.9×10^{-2}
Ubiquitin-dependent protein catabolic process (GO BP)	6	10.5	7.1	1.6×10^{-1}
Deubiquitination (Reactome)	6	10.5	4.7	2.1×10^{-1}
Protease (UniProtKB Keywords)	6	10.5	3.0	3.4×10^{-1}
Ub-specific processing proteases (Reactome)	5	8.8	5.3	2.4×10^{-1}
Protein ubiquitination (GO BP)	5	8.8	3.7	9.3×10^{-1}
Cysteine-type endopeptidase activity (GO MF)	4	7.0	13.3	9.6×10^{-2}
ER to Golgi anterograde transport (Reactome)	4	7.0	6.1	3.7×10^{-1}
Transport to the Golgi and subsequent modification (Reactome)	4	7.0	5.0	4.7×10^{-1}
Monoubiquitinated protein deubiquitination (GO BP)	3	5.3	127.7	1.2×10^{-1}
Ubiquitinyl hydrolase activity (GO MF)	3	5.3	76.7	3.9×10^{-2}
Lys48-specific deubiquitinase activity (GO MF)	3	5.3	55.4	5.7×10^{-2}
Golgi associated vesicle biogenesis (Reactome)	3	5.3	12.5	3.3×10^{-1}
trans-Golgi network vesicle budding (Reactome)	3	5.3	9.7	4.7×10^{-1}
Upregulated after CPP-S treatment				
Metabolism of RNA (Reactome)	9	13.6	2.9	1.0×10^{-1}
mRNA splicing (UniProtKB Keywords)	6	9.1	6.6	6.5×10^{-2}
mRNA processing (UniProtKB Keywords)	6	9.1	5.1	9.8×10^{-2}
mRNA 3'-UTR binding (GO MF)	5	7.6	5.9	2.7×10^{-1}

mRNA binding (GO MF)	5	7.6	5.8	2.7×10^{-1}
Programmed cell death (Reactome)	5	7.6	5.5	1.0×10^{-1}
Positive regulation of apoptotic process (GO BP)	5	7.6	4.7	1.0×10^{-1}
Negative regulation of translation (GO BP)	4	6.1	13.8	5.5×10^{-1}
Apoptosis (Reactome)	4	6.1	5.2	1.0×10^{-1}
mRNA transport (UniProtKB Keywords)	3	4.5	10.7	1.0×10^{-1}

Supplementary Table S7. Fold enrichment (observed-versus-expected) analysis of differentially expressed proteins in nuclei of human coronary artery endothelial cells (HCAEC) treated with either primary CPPs (CPP-P) or control phosphate-buffer saline (PBS).

**Molecular term**	**Total proteins**	**Percent from differentially expressed proteins**	**Fold enrichment**	**FDR-corrected P value**
Upregulated after CPP-P treatment				
Cytokine signaling in immune system (Reactome)	12	15.4	2.9	6.0×10^{-2}
DNA repair (Reactome)	10	12.8	5.4	1.6×10^{-2}
Ubiquitin-dependent protein catabolic process (GO BP)	9	11.5	7.9	7.6×10^{-3}
Ubiquitin protein ligase binding (GO MF)	9	11.5	7.0	1.7×10^{-3}
Protein ubiquitination (GO BP)	8	10.3	4.3	2.4×10^{-1}
Apoptosis (UniProtKB Keywords)	8	10.3	2.6	1.8×10^{-1}
DNA damage (UniProtKB Keywords)	7	9.0	3.0	1.8×10^{-1}
Apoptotic process (GO BP)	7	9.0	2.9	9.2×10^{-1}
Signaling by interleukins (Reactome)	7	9.0	2.7	3.7×10^{-1}
Chemokine signaling pathway (KEGG)	5	6.4	4.2	1.5×10^{-1}
Ubiquitin mediated proteolysis (KEGG)	5	6.4	5.6	9.2×10^{-2}
Ca-dependent events (Reactome)	4	5.1	18.4	5.0×10^{-2}
VEGFA-VEGFR2 pathway (Reactome)	4	5.1	7.3	2.0×10^{-1}
Signaling by VEGF (Reactome)	4	5.1	6.7	2.2×10^{-1}
Intrinsic apoptotic signaling pathway (GO BP)	3	3.8	21.0	4.6×10^{-1}
Downregulated after CPP-P treatment				
Metabolism of RNA (Reactome)	69	37.3	7.4	1.6×10^{-40}
mRNA processing (UniProtKB Keywords)	52	28.1	10.9	5.3×10^{-38}
mRNA splicing (UniProtKB Keywords)	46	24.9	12.5	5.3×10^{-36}
Gene expression (Transcription) (Reactome)	37	20.0	1.8	4.0×10^{-3}
Cell cycle, mitotic (Reactome)	18	9.7	2.5	1.1×10^{-2}
mRNA transport (UniProtKB Keywords)	17	9.2	12.3	7.4×10^{-12}
Positive regulation of transcription, DNA-templated (GO BP)	17	9.2	2.5	4.9×10^{-2}
Cadherin binding (GO MF)	13	7.0	4.2	2.9×10^{-3}
Negative regulation of apoptotic process (GO BP)	13	7.0	2.7	1.1×10^{-1}
Mitotic metaphase and anaphase (Reactome)	12	6.5	3.9	4.1×10^{-3}
mRNA surveillance pathway (KEGG)	11	5.9	9.8	4.8×10^{-6}
Mitotic prophase (Reactome)	9	4.9	4.9	6.7×10^{-3}
Cell cycle checkpoints (Reactome)	9	4.9	2.4	1.7×10^{-1}
Assembly of the pre-replicative complex (Reactome)	8	4.3	4.3	2.3×10^{-2}
DNA replication (Reactome)	8	4.3	3.3	7.7×10^{-2}

Supplementary Table S8. Fold enrichment (observed-versus-expected) analysis of differentially expressed proteins in nuclei of human coronary artery endothelial cells (HCAEC) treated with either secondary CPPs (CPP-S) or control phosphate-buffer saline (PBS).

Molecular term	Total proteins	Percent from differentially expressed proteins	Fold enrichment	FDR-corrected P value
Upregulated after CPP-S treatment				
Hydrolase (UniProtKB Keywords)	13	17.1	1.8	7.0×10^{-1}
Cytokine signaling in immune system (Reactome)	12	15.8	3.1	4.7×10^{-2}
Ubiquitin-dependent protein catabolic process (GO BP)	8	10.5	7.1	2.2×10^{-2}
Protein ubiquitination (GO BP)	8	10.5	4.4	1.8×10^{-1}
Signaling by interleukins (Reactome)	8	10.5	3.2	1.2×10^{-1}
Apoptosis (UniProtKB Keywords)	8	10.5	2.8	1.6×10^{-1}
Apoptotic process (GO BP)	7	9.2	2.9	8.5×10^{-1}
Chemokine signaling pathway (KEGG)	6	7.9	5.3	2.0×10^{-2}
Toll like receptor 4 (TLR4) cascade (Reactome)	5	6.6	6.5	1.0×10^{-1}
Positive regulation of apoptotic process (GO BP)	5	6.6	3.9	8.5×10^{-1}
Intrinsic apoptotic signaling pathway (GO BP)	4	5.3	28.4	4.9×10^{-2}
Ca-dependent events (Reactome)	4	5.3	19.4	4.5×10^{-2}
Signaling by VEGF (Reactome)	4	5.3	7.0	1.5×10^{-1}
Cellular response to reactive oxygen species (GO BP)	3	3.9	15.3	6.0×10^{-1}
Cellular response to hydrogen peroxide (GO BP)	3	3.9	11.8	7.9×10^{-1}
Downregulated after CPP-S treatment				
Metabolism of RNA (Reactome)	86	44.3	8.8	1.8×10^{-59}
mRNA processing (UniProtKB Keywords)	63	32.5	12.1	4.7×10^{-50}
mRNA splicing (UniProtKB Keywords)	55	28.4	13.6	4.5×10^{-46}
Gene expression (Transcription) (Reactome)	44	22.7	2.1	2.6×10^{-5}
Cell cycle, mitotic (Reactome)	21	10.8	2.7	5.5×10^{-4}
mRNA transport (UniProtKB Keywords)	17	8.8	11.2	2.2×10^{-11}
Mitotic metaphase and anaphase (Reactome)	14	7.2	4.3	1.9×10^{-4}
Cadherin binding (GO MF)	14	7.2	4.3	7.0×10^{-4}
Mitotic prophase (Reactome)	12	6.2	6.2	3.8×10^{-5}
Negative regulation of apoptotic process (GO BP)	12	6.2	2.3	2.9×10^{-1}
Cell cycle checkpoints (Reactome)	11	5.7	2.7	2.7×10^{-2}
mRNA surveillance pathway (KEGG)	10	5.2	8.0	1.0×10^{-4}
Assembly of the pre-replicative complex (Reactome)	7	3.6	3.6	4.2×10^{-2}
DNA replication (Reactome)	7	3.6	2.7	1.1×10^{-1}
mRNA export from nucleus (GO BP)	6	3.1	10.2	1.2×10^{-2}

Supplementary Table S9. Fold enrichment (observed-versus-expected) analysis of differentially expressed proteins in nuclei of human internal thoracic artery endothelial cells (HITAEC) treated with either primary CPPs (CPP-P) or control phosphate-buffer saline (PBS).

Molecular term	Total proteins	Percent from differentially expressed proteins	Fold enrichment	FDR-corrected P value
Upregulated after CPP-P treatment				
Metabolism of RNA (Reactome)	7	20.0	3.7	8.5×10^{-1}
mRNA splicing (UniProtKB Keywords)	5	14.3	7.7	1.2×10^{-1}
mRNA processing (UniProtKB Keywords)	5	14.3	6.0	1.3×10^{-1}
Nucleocytoplasmic transport (KEGG)	4	11.4	18.4	1.0×10^{-1}
Spliceosome (KEGG)	4	11.4	9.2	3.7×10^{-1}
Regulation of mRNA processing (GO BP)	3	8.6	107.1	5.9×10^{-2}

mRNA export from nucleus (GO BP)	3	8.6	28.6	3.0×10^{-1}
Positive regulation of epithelial to mesenchymal transition (GO BP)	3	8.6	31.7	3.0×10^{-1}
mRNA transport (GO BP)	3	8.6	19.9	3.7×10^{-1}
mRNA 3′-end processing (Reactome)	3	8.6	19.2	8.5×10^{-1}
RNA polymerase II transcription termination (Reactome)	3	8.6	16.7	8.5×10^{-1}
mRNA surveillance pathway (KEGG)	3	8.6	15.4	4.8×10^{-1}
Positive regulation of NF-κB transcription factor activity (GO BP)	3	8.6	10.4	9.5×10^{-1}
Response to elevated platelet cytosolic Ca^{2+} (Reactome)	3	8.6	8.5	1.0×10^{-1}
Calcium-mediated signaling using intracellular calcium source (GO BP)	2	5.7	49.7	9.9×10^{-1}
Downregulated after CPP-P treatment				
Metabolism of RNA (Reactome)	21	25.0	5.2	2.7×10^{-7}
mRNA processing (UniProtKB Keywords)	18	21.4	7.8	2.1×10^{-9}
mRNA splicing (UniProtKB Keywords)	16	19.0	9.0	2.8×10^{-9}
Gene expression (Transcription) (Reactome)	15	17.9	1.7	8.1×10^{-1}
Transcription cofactor activity (GO MF)	8	9.5	12.7	1.2×10^{-4}
mRNA transport (UniProtKB Keywords)	8	9.5	12.0	6.0×10^{-5}
Transcription, DNA-templated (GO BP)	8	9.5	9.5	2.8×10^{-3}
Nuclear envelope reassembly (Reactome)	5	6.0	11.8	4.6×10^{-2}
Nucleocytoplasmic transport (KEGG)	5	6.0	10.9	2.7×10^{-2}
Mitotic metaphase and anaphase (Reactome)	5	6.0	3.7	8.1×10^{-1}
mRNA export from nucleus (GO BP)	4	4.8	15.6	1.9×10^{-1}
Nuclear envelope breakdown (Reactome)	4	4.8	13.6	1.2×10^{-1}
mRNA surveillance pathway (KEGG)	4	4.8	9.7	1.1×10^{-1}
Mitotic prophase (Reactome)	4	4.8	5.0	8.1×10^{-1}
Stem cell division (GO BP)	2	2.4	66.8	7.6×10^{-1}

Supplementary Table S10. Fold enrichment (observed-versus-expected) analysis of differentially expressed proteins in nuclei of human internal thoracic artery endothelial cells (HITAEC) treated with either secondary CPPs (CPP-S) or control phosphate-buffer saline (PBS).

Molecular term	Total proteins	Percent from differentially expressed proteins	Fold enrichment	FDR-corrected P value
Upregulated after CPP-S treatment				
Metabolism of RNA (Reactome)	10	33.3	6.7	1.7 × 10-3
mRNA splicing (UniProtKB Keywords)	7	23.3	11.8	4.7 × 10-4
mRNA processing (UniProtKB Keywords)	7	23.3	9.1	1.0 × 10-3
RNA polymerase II transcription (Reactome)	7	23.3	2.4	8.0 × 10-1
Calcium ion binding (GO MF)	5	16.7	4.2	3.6 × 10-1
Nucleocytoplasmic transport (KEGG)	4	13.3	24.1	1.9 × 10-2
Regulation of mRNA processing (GO BP)	3	10.0	121.3	4.6 × 10-2
mRNA export from nucleus (GO BP)	3	10.0	32.4	3.3 × 10-1
mRNA transport (GO BP)	3	10.0	22.6	3.7 × 10-1
Regulation of translation (GO BP)	3	10.0	22.3	3.7 × 10-1
mRNA surveillance pathway (KEGG)	3	10.0	20.1	1.8 × 10-1
RNA polymerase II transcription termination (Reactome)	3	10.0	21.0	3.6 × 10-1
tRNA processing (Reactome)	3	10.0	13.4	4.5 × 10-1
Response to elevated platelet cytosolic Ca^{2+} (Reactome)	3	10.0	10.7	4.9 × 10-1
Spliceosome (KEGG)	3	10.0	9.0	5.6 × 10-1
Downregulated after CPP-S treatment				
Metabolism of RNA (Reactome)	24	36.4	6.8	1.6×10^{-11}

Gene expression (Transcription) (Reactome)	16	24.2	2.1	4.4×10^{-2}
mRNA processing (UniProtKB Keywords)	11	16.7	5.6	2.9×10^{-4}
mRNA splicing (UniProtKB Keywords)	10	15.2	6.6	2.9×10^{-4}
Negative regulation of apoptotic process (GO BP)	8	12.1	4.5	9.2×10^{-2}
Nucleocytoplasmic transport (KEGG)	7	10.6	13.1	2.0×10^{-3}
Cadherin binding (GO MF)	7	10.6	6.3	2.9×10^{-2}
Cell adhesion (UniProtKB Keywords)	7	10.6	2.9	1.0×10^{-1}
tRNA processing (Reactome)	6	9.1	11.4	3.2×10^{-3}
mRNA transport (UniProtKB Keywords)	6	9.1	10.6	2.6×10^{-3}
DNA repair (GO BP)	6	9.1	5.9	1.3×10^{-1}
mRNA export from nucleus (GO BP)	4	6.1	19.6	6.9×10^{-2}
rRNA processing (GO BP)	4	6.1	8.7	3.3×10^{-1}
Ribosome biogenesis (UniProtKB Keywords)	4	6.1	8.3	4.4×10^{-2}
Positive regulation of translation (GO BP)	3	4.5	10.5	6.5×10^{-1}

Supplementary Table S11. Fold enrichment (observed-versus-expected) analysis of differentially expressed proteins in nuclei of human internal thoracic artery endothelial cells (HITAEC) treated with either primary CPPs (CPP-P) or secondary CPPs (CPP-S).

Molecular term	Total proteins	Percent from differentially expressed proteins	Fold enrichment	FDR-corrected P value
Upregulated after CPP-P treatment				
Metabolism of proteins (Reactome)	17	32.7	2.4	2.7×10^{-2}
Metabolism of RNA (Reactome)	16	30.8	6.1	2.8×10^{-6}
Gene expression (Transcription) (Reactome)	13	25.0	2.3	6.9×10^{-2}
Hydrolase (UniProtKB Keywords)	12	23.1	2.1	1.6×10^{-1}
DNA repair (GO BP)	7	13.5	9.0	2.9×10^{-2}
mRNA processing (UniProtKB Keywords)	7	13.5	4.9	2.1×10^{-2}
Protein transport (UniProtKB Keywords)	7	13.5	3.0	1.1×10^{-1}
Nucleocytoplasmic transport (KEGG)	6	11.5	13.8	6.8×10^{-3}
mRNA splicing (Reactome)	6	11.5	7.6	2.7×10^{-2}
DNA damage (UniProtKB Keywords)	6	11.5	3.9	7.9×10^{-2}
mRNA transport (UniProtKB Keywords)	5	9.6	12.0	1.2×10^{-2}
Cellular response to DNA damage stimulus (GO BP)	5	9.6	6.5	3.2×10^{-1}
tRNA processing in the nucleus (Reactome)	4	7.7	19.2	2.7×10^{-2}
Ribosome biogenesis (UniProtKB Keywords)	3	5.8	8.5	1.6×10^{-1}
rRNA processing (GO BP)	3	5.8	8.4	7.9×10^{-1}
Upregulated after CPP-S treatment				
Metabolism of RNA (Reactome)	15	34.1	7.2	5.8×10^{-7}
mRNA splicing (Reactome)	12	27.3	13.1	1.2×10^{-8}
mRNA processing (Reactome)	12	27.3	10.1	8.8×10^{-8}
Gene expression (Transcription) (Reactome)	12	27.3	2.7	9.2×10^{-2}
RNA polymerase II Transcription (Reactome)	10	22.7	2.5	2.9×10^{-1}
Regulation of transcription, DNA-templated (GO BP)	7	15.9	3.1	6.1×10^{-1}
Spliceosome (KEGG)	6	13.6	11.7	2.7×10^{-3}
mRNA transport (UniProtKB Keywords)	5	11.4	14.5	2.5×10^{-3}
mRNA export from nucleus (GO BP)	3	6.8	22.6	2.9×10^{-1}
Nucleocytoplasmic transport (KEGG)	3	6.8	11.8	2.4×10^{-1}

Supplementary Table S12. Fold enrichment (observed-versus-expected) analysis of differentially expressed proteins in mitochondria of human internal thoracic artery endothelial cells (HITAEC) treated with either primary CPPs (CPP-P) or control phosphate-buffer saline (PBS).

**Molecular term**	**Total proteins**	**Percent from differentially expressed proteins**	**Fold enrichment**	**FDR-corrected P value**
Upregulated after CPP-P treatment				
Transmembrane transporter activity (GO MF)	3	15.0	19.1	3.4×10^{-1}
Ubiquitin protein ligase binding (GO MF)	3	15.0	9.6	6.8×10^{-1}
Mitochondrial transmembrane transport (GO BP)	2	10.0	204.4	1.0×10^{-1}
Ion transmembrane transporter activity (GO MF)	2	10.0	142.4	3.4×10^{-1}
Inner mitochondrial membrane organization (GO BP)	2	10.0	97.3	1.0×10^{-1}
Downregulated after CPP-P treatment				
Metabolism (Reactome)	10	55.6	3.7	4.2×10^{-3}
Oxidoreductase (UniProtKB Keywords)	6	33.3	7.7	7.5×10^{-3}
Metabolism of amino acids and derivatives (Reactome)	4	22.2	8.3	2.0×10^{-1}
Amino-acid biosynthesis (UniProtKB Keywords)	3	16.7	92.8	4.6×10^{-3}
Cellular response to oxidative stress (GO BP)	3	16.7	34.8	1.7×10^{-1}

Supplementary Table S13. Fold enrichment (observed-versus-expected) analysis of differentially expressed proteins in lysosomes of human coronary artery endothelial cells (HCAEC) treated with either primary CPPs (CPP-P) or control phosphate-buffer saline (PBS).

**Molecular term**	**Total proteins**	**Percent from differentially expressed proteins**	**Fold enrichment**	**FDR-corrected P value**
Upregulated after CPP-P treatment				
Hydrolase (UniProtKB Keywords)	9	45.0	4.2	9.5×10^{-4}
Innate immune system (Reactome)	8	40.0	5.1	5.0×10^{-3}
Glycosidase (UniProtKB Keywords)	5	25.0	50.6	1.7×10^{-5}
Dipeptidyl-peptidase activity (GO MF)	2	10.0	199.4	2.2×10^{-1}
Keratan sulfate degradation (Reactome)	2	10.0	105.4	1.0×10^{-1}
Downregulated after CPP-P treatment				
Hydrolase (UniProtKB Keywords)	5	50.0	6.6	4.2×10^{-3}
Innate immune system (Reactome)	4	40.0	4.1	7.2×10^{-1}
Glycosaminoglycan degradation (KEGG)	2	20.0	127.3	1.0×10^{-1}
Autophagosome maturation (GO BP)	2	20.0	100.3	7.8×10^{-1}
Endosome to lysosome transport (GO BP)	2	20.0	86.3	7.8×10^{-1}

Supplementary Table S14. Fold enrichment (observed-versus-expected) analysis of differentially expressed proteins in lysosomes of human coronary artery endothelial cells (HCAEC) treated with either secondary CPPs (CPP-S) or control phosphate-buffer saline (PBS).

**Molecular term**	**Total proteins**	**Percent from differentially expressed proteins**	**Fold enrichment**	**FDR-corrected P value**
Upregulated after CPP-S treatment				
Hydrolase (UniProtKB Keywords)	4	36.4	3.8	1.9×10^{-1}
Hydrolase activity (GO MF)	3	27.3	20.9	2.6×10^{-1}
Glycosaminoglycan degradation (KEGG)	2	18.2	99.0	2.2×10^{-1}
Glycosidase (UniProtKB Keywords)	2	18.2	40.4	1.9×10^{-1}
Protein processing (GO BP)	2	18.2	41.5	1.0×10^{-1}
Downregulated after CPP-S treatment				

None

Supplementary Table S15. Fold enrichment (observed-versus-expected) analysis of differentially expressed proteins in endoplasmic reticulum of human internal thoracic artery endothelial cells (HITAEC) treated with either primary CPPs (CPP-P) or secondary CPPs (CPP-S).

Molecular term	Total proteins	Percent from differentially expressed proteins	Fold enrichment	FDR-corrected P value
Upregulated after CPP-P treatment				
Protein processing in endoplasmic reticulum (GO BP)	6	15.0	11.7	5.1×10^{-3}
Ubiquitin-mediated endoplasmic-reticulum-associated protein degradation pathway (GO BP)	4	10.0	24.0	1.0×10^{-1}
Response to calcium ion (GO BP)	3	7.5	27.0	3.5×10^{-1}
Response to endoplasmic reticulum stress (GO BP)	3	7.5	17.3	6.2×10^{-1}
Metalloexopeptidase activity (GO MF)	2	5.0	48.6	9.5×10^{-1}
Upregulated after CPP-S treatment				
None				

Supplementary Table S16. Fold enrichment (observed-versus-expected) analysis of differentially expressed proteins in mitochondrial lysate of human coronary artery endothelial cells (HCAEC) treated with either primary CPPs (CPP-P) or secondary CPPs (CPP-S).

Molecular term	Total proteins	Percent from differentially expressed proteins	Fold enrichment	FDR-corrected P value
Upregulated after CPP-P treatment				
Cellular responses to stress (Reactome)	31	55.4	8.3	1.8×10^{-21}
Cellular responses to stimuli (Reactome)	31	55.4	8.1	3.0×10^{-21}
Cellular senescence (Reactome)	29	51.8	31.2	9.4×10^{-36}
Diseases of programmed cell death (Reactome)	28	50.0	56.7	1.7×10^{-41}
Senescence-associated secretory phenotype (Reactome)	27	48.2	51.3	7.5×10^{-39}
Oxidative stress-induced senescence (Reactome)	27	48.2	45.9	1.5×10^{-37}
DNA damage/Telomere stress-induced senescence (Reactome)	19	33.9	50.7	2.5×10^{-26}
Depurination (Reactome)	17	30.4	65.1	2.7×10^{-25}
Depyrimidination (Reactome)	17	30.4	59.7	1.2×10^{-24}
Apoptotic execution phase (Reactome)	3	5.4	12.2	3.8×10^{-2}
Upregulated after CPP-S treatment				
None				

Supplementary Table S17. Fold enrichment (observed-versus-expected) analysis of differentially expressed proteins in nuclear lysate of human coronary artery endothelial cells (HCAEC) treated with either primary CPPs (CPP-P) or control phosphate-buffer saline (PBS).

Molecular term	Total proteins	Percent from differentially expressed proteins	Fold enrichment	FDR-corrected P value
Upregulated after CPP-P treatment				
Cellular responses to stress (Reactome)	43	26.9	4.1	2.9×10^{-14}
Protein folding (GO BP)	13	8.1	8.7	9.7×10^{-6}
Unfolded protein binding (GO MF)	12	7.5	11.0	5.5×10^{-7}
Chaperone (UniProtKB Keywords)	10	6.2	4.0	5.3×10^{-3}
Chaperonin-mediated protein folding (Reactome)	10	6.2	8.2	2.6×10^{-5}
Angiogenesis (GO BP)	9	5.6	4.3	5.2×10^{-2}

Chemical carcinogenesis - reactive oxygen species (KEGG)	9	5.6	2.5	2.1×10^{-1}
Nuclear envelope reassembly (Reactome)	7	4.4	7.0	2.1×10^{-3}
Cellular senescence (KEGG)	7	4.4	2.8	2.2×10^{-1}
Necroptosis (KEGG)	7	4.4	2.8	2.3×10^{-1}
Sealing of the nuclear envelope (NE) by ESCRT-III (Reactome)	6	3.8	14.5	3.1×10^{-4}
Chaperone-mediated protein folding (GO BP)	6	3.8	18.9	1.6×10^{-3}
Response to oxidative stress (GO BP)	5	3.1	5.0	3.8×10^{-1}
Positive regulation of cysteine-type endopeptidase activity involved in apoptotic process (GO BP)	4	2.5	9.6	2.3×10^{-1}
Response to unfolded protein (GO BP)	4	2.5	8.5	2.8×10^{-1}
Downregulated after CPP-P treatment				
Transcription regulation (UniProtKB Keywords)	15	33.3	2.0	7.6×10^{-2}
mRNA processing (UniProtKB Keywords)	12	26.7	9.6	2.3×10^{-7}
mRNA splicing (UniProtKB Keywords)	11	24.4	11.4	2.3×10^{-7}
mRNA splicing, via spliceosome (GO BP)	9	20.0	20.3	2.1×10^{-6}
Cell cycle, mitotic (Reactome)	6	13.3	4.0	1.2×10^{-1}
Positive regulation of transcription, DNA-templated (GO BP)	6	13.3	3.7	5.8×10^{-1}
mRNA binding (GO MF)	5	11.1	8.6	1.0×10^{-1}
Chromatic modifying enzymes (Reactome)	5	11.1	6.9	6.7×10^{-2}
Homology directed repair (Reactome)	4	8.9	11.0	6.7×10^{-2}
DNA double-strand break repair (Reactome)	4	8.9	9.0	8.9×10^{-2}
Transcription coactivator activity (GO MF)	4	8.9	6.5	3.1×10^{-1}
Cell cycle checkpoints (Reactome)	4	8.9	5.2	2.0×10^{-1}
Base excision repair (Reactome)	3	6.7	12.5	1.5×10^{-1}
Transcription cofactor activity (GO MF)	3	6.7	9.1	4.3×10^{-1}
mRNA transport (UniProtKB Keywords)	3	6.7	8.2	1.5×10^{-1}

Supplementary Table S18. Fold enrichment (observed-versus-expected) analysis of differentially expressed proteins in nuclear lysate of human coronary artery endothelial cells (HCAEC) treated with either secondary CPPs (CPP-S) or control phosphate-buffer saline (PBS).

**Molecular term**	**Total proteins**	**Percent from differentially expressed proteins**	**Fold enrichment**	**FDR-corrected P value**
Upregulated after CPP-S treatment				
Protein binding involved in protein folding (GO MF)	8	5.6	20.6	3.2×10^{-6}
Cellular responses to stress (Reactome)	41	28.5	4.2	4.0×10^{-13}
Unfolded protein binding (GO MF)	14	9.7	14.2	1.1×10^{-9}
Chaperone (UniProtKB Keywords)	12	8.3	5.4	1.3×10^{-4}
Response to elevated cytosolic Ca^{2+} (Reactome)	10	6.9	6.0	2.5×10^{-4}
Angiogenesis (GO BP)	10	6.9	5.3	8.3×10^{-3}
Protein folding (Reactome)	9	6.2	7.5	1.7×10^{-4}
Chaperonin-mediated protein folding (Reactome)	9	6.2	8.0	1.1×10^{-4}
Apoptosis (Reactome)	9	6.2	4.0	7.6×10^{-3}
Programmed cell death (Reactome)	9	6.2	3.4	1.9×10^{-2}
Nuclear envelope reassembly (Reactome)	9	6.2	9.7	3.0×10^{-5}
Cellular response to heat stress (Reactome)	7	4.9	6.3	3.9×10^{-3}
Fluid shear stress and atherosclerosis (KEGG)	7	4.9	3.4	9.3×10^{-2}
Sealing of the nuclear envelope by ESCRT-III (Reactome)	6	4.2	15.6	2.2×10^{-4}
Stress response (UniProtKB Keywords)	6	4.2	6.7	2.2×10^{-2}
Chaperone-mediated protein folding (GO BP)	5	3.5	17.3	1.2×10^{-2}
Response to unfolded protein (GO BP)	5	3.5	11.6	3.7×10^{-2}
Apoptotic execution phase (Reactome)	5	3.5	7.7	1.6×10^{-2}
Apoptotic cleavage of cellular proteins (Reactome)	4	2.8	8.5	4.2×10^{-2}

Cellular response to unfolded protein (GO BP)	3	2.1	14.4	3.4×10^{-4}
Downregulated after CPP-S treatment				
Gene expression (Transcription) (Reactome)	22	33.8	3.3	4.2×10^{-7}
Cell cycle, mitotic (Reactome)	19	29.2	7.9	7.4×10^{-12}
Transcriptional regulation by small RNAs (Reactome)	17	26.2	37.7	5.0×10^{-20}
Chromatic modifying enzymes (Reactome)	17	26.2	14.6	2.6×10^{-14}
mRNA transport (UniProtKB Keywords)	7	10.8	22.3	1.7×10^{-5}
Transport of mature mRNA derived from an intron-containing transcript (Reactome)	6	9.2	18.7	2.3×10^{-5}
Transport of mature transcript to cytoplasm (Reactome)	6	9.2	16.7	3.8×10^{-5}
Transport of mature mRNA derived from an intronless transcript (Reactome)	4	6.2	22.7	7.2×10^{-4}
mRNA export from nucleus (GO BP)	4	6.2	20.5	1.8×10^{-2}
snRNP assembly (Reactome)	4	6.2	17.6	1.4×10^{-3}
Metabolism of non-coding RNA (Reactome)	4	6.2	17.6	1.4×10^{-3}
tRNA processing in the nucleus (Reactome)	4	6.2	17.6	1.7×10^{-3}
Base excision repair (Reactome)	4	6.2	10.2	6.5×10^{-3}
Homology directed repair (Reactome)	4	6.2	6.8	2.0×10^{-2}
DNA double-strand break repair (Reactome)	4	6.2	5.6	3.3×10^{-2}

Supplementary Table S19. Fold enrichment (observed-versus-expected) analysis of differentially expressed proteins in nuclear lysate of human coronary artery endothelial cells (HCAEC) treated with either primary CPPs (CPP-P) or secondary CPPs (CPP-S).

Molecular term	Total proteins	Percent from differentially expressed proteins	Fold enrichment	FDR-corrected P value
Upregulated after CPP-P treatment				
Cellular responses to stress (Reactome)	34	68.0	10.3	3.4×10^{-28}
Gene expression (Transcription) (Reactome)	28	56.0	4.3	5.4×10^{-12}
Cellular senescence (Reactome)	28	56.0	34.0	5.4×10^{-36}
Transcriptional regulation by small RNAs (Reactome)	27	54.0	61.3	3.7×10^{-41}
Cell cycle, mitotic (Reactome)	27	54.0	11.5	3.1×10^{-22}
Diseases of programmed cell death (Reactome)	25	50.0	57.3	2.9×10^{-37}
Senescence-associated secretory phenotype (SASP) (Reactome)	25	50.0	53.6	1.3×10^{-36}
Oxidative stress induced senescence (Reactome)	25	50.0	48.0	2.1×10^{-35}
Assembly of the pre-replicative complex (Reactome)	25	50.0	41.9	6.6×10^{-34}
DNA replication (Reactome)	25	50.0	31.8	7.1×10^{-31}
DNA damage/Telomere stress induced senescence (Reactome)	18	36.0	54.3	1.4×10^{-25}
Base excision repair (Reactome)	15	30.0	39.3	6.1×10^{-19}
Homology directed repair (Reactome)	15	30.0	25.9	2.5×10^{-16}
DNA double-strand break repair (Reactome)	15	30.0	21.3	4.0×10^{-15}
Cell cycle checkpoints (Reactome)	15	30.0	12.2	8.3×10^{-12}
Eukaryotic translation termination (Reactome)	4	8.0	10.1	8.1×10^{-3}
Eukaryotic translation elongation (Reactome)	4	8.0	10.1	8.1×10^{-3}
Eukaryotic translation initiation (Reactome)	4	8.0	7.9	1.4×10^{-2}
Apoptosis induced DNA fragmentation (Reactome)	3	6.0	55.0	1.7×10^{-3}
Apoptotic execution phase (Reactome)	3	6.0	13.7	2.1×10^{-2}
Upregulated after CPP-S treatment				
mRNA binding (GO MF)	3	33.3	25.2	8.8×10^{-2}
mRNA splicing (Reactome)	3	33.3	21.8	1.1×10^{-1}
Processing of capped intron-containing pre-mRNA (Reactome)	3	33.3	16.5	1.5×10^{-1}

Regulation of alternative mRNA splicing, via spliceosome (GO BP)	2	22.2	67.4	1.0×10^{-1}
Double-stranded RNA binding (GO MF)	2	22.2	56.9	3.7×10^{-1}
